# Liquid Biopsy Instrument for Ultra-Fast and Label-Free Detection of Circulating Tumor Cells

**DOI:** 10.34133/research.0431

**Published:** 2024-07-24

**Authors:** Shu Zhu, Zhixian Zhu, Chen Ni, Zheng Zhou, Yao Chen, Dezhi Tang, Kefan Guo, Shuai Yang, Kang Liu, Zhonghua Ni, Nan Xiang

**Affiliations:** ^1^ School of Mechanical Engineering, and Jiangsu Key Laboratory for Design and Manufacture of Micro-Nano Biomedical Instruments, Southeast University, Nanjing 211189, China.; ^2^ School of Electrical and Automation Engineering, and Jiangsu Key Laboratory of 3D Printing Equipment and Manufacturing, Nanjing Normal University, Nanjing 210023, China.

## Abstract

Rapid diagnosis and real-time monitoring are of great important in the fight against cancer. However, most available diagnostic technologies are time-consuming and labor-intensive and are commonly invasive. Here, we describe CytoExam, an automatic liquid biopsy instrument designed based on inertial microfluidics and impedance cytometry, which uses a deep learning algorithm for the analysis of circulating tumor cells (CTCs). In silico and in vitro experiments demonstrated that CytoExam could achieve label-free detection of CTCs in the peripheral blood of cancer patients within 15 min. The clinical applicability of CytoExam was also verified using peripheral blood samples from 10 healthy donors and >50 patients with breast, colorectal, or lung cancer. Significant differences in the number of collected cells and predicted CTCs were observed between the 2 groups, with variations in the dielectric properties of the collected cells from cancer patients also being observed. The ultra-fast and minimally invasive features of CytoExam may pave the way for new paths for cancer diagnosis and scientific research.

## Introduction

Circulating tumor cells (CTCs) that escape from the primary tumor and enter the blood circulation are considered the culprits of cancer metastasis [1,2]. Owing to CTC origin, it is expected that they provide valuable information on tumor composition [3], invasiveness [4], drug susceptibility [5], and treatment resistance [6]. As a promising biomarker of tumor liquid biopsies, CTCs can be obtained from peripheral blood through less invasive procedures than tumor tissue biopsies [7,8]. Therefore, achieving rapid detection and assessment of CTCs is meaningful for the diagnosis and monitoring of metastatic carcinomas [9].

Given CTC scarcity in the peripheral blood and their similarity with healthy blood cells, direct detection and acquisition of CTCs face huge technical challenges [10]. Specific staining based on the immune-affinity method is currently the main approach for CTC enrichment and detection after label-free sorting [11], with several instruments being available, such as CellSearch (Menarini Silicon Biosystems, Italy) [12,13], CytoSorter (Watson Biotech, China) [14,15], and EasySep (Stemcell Technologies, Canada) [16], and with the epithelial cell adhesion molecule (EpCAM) being the most widely used biomarker [17]. However, the fundamental limitation is that a subpopulation of metastatic tumor cells undergoing epithelial-to-mesenchymal transition will lose EpCAM expression [18], while some leukocytes may be activated for EpCAM [19], causing inaccurate detection using this approach. Additionally, immune-affinity techniques commonly require expensive biochemical reagents and are labor-intensive and time-consuming (2 to 3 days) [20,21]. Furthermore, death and irreversible harm to cells could be induced using immune-affinity techniques, preventing their downstream biochemical use [22].

Different from immune-affinity techniques, label-free cell detection and sorting methods commonly depend on the cell physical features [23,24] and require low-cost, simple operation with high cell viability [25]. Although many instruments for CTC label-free sorting have been developed, such as Parsortix (ANGLE, UK) [26] and ClearCell FX (Biolidics, Singapore) [27], the identification of collected CTCs still relies on immune staining techniques. The first challenge for CTC label-free detection is its low detection throughput [28]. Even if the throughput of label-free detection techniques could reach hundred to thousand cells per second, which is similar to that of image and impedance flow cytometry [7,29], it would still be far from meeting the requirements for their application to cancer clinical samples with cells over 10 million orders of magnitudes [30]. The second challenge is their low detection accuracy [31]. It is reported that some white blood cells (WBCs) share similar physical features with CTCs (e.g., size and deformability) [32], which may prevent the identification of rare CTCs from high background WBCs. The third challenge is the inefficiency of the cell label-free detection process [33]. Existing cell label-free detection technologies are limited to the chip level [34], in which a lot of manual operation, such as sample transfer [35], cannot be avoided and consequently causes a tedious detection process [36].

**Fig. 1.**
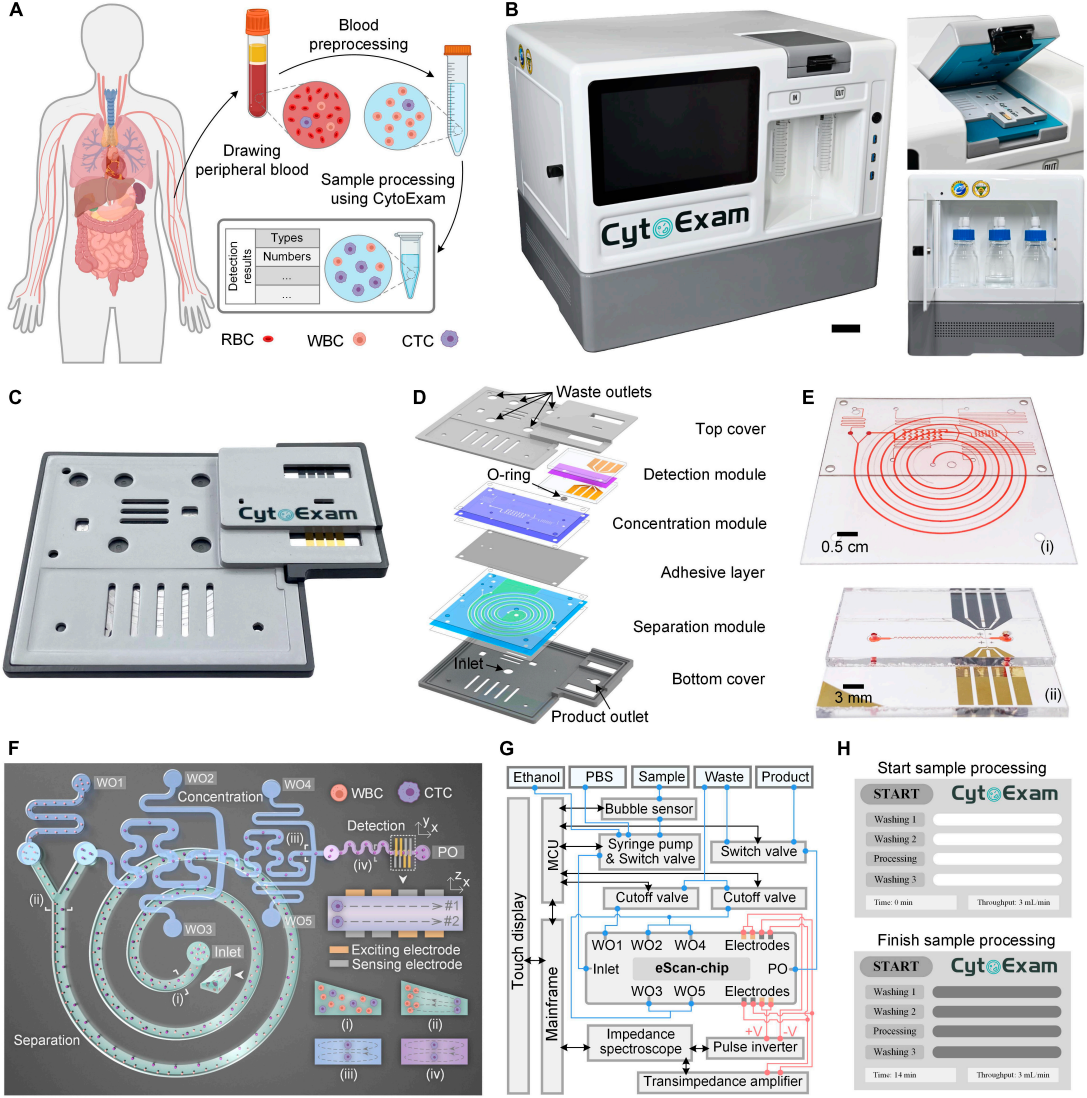
Novel instrumentation for label-free detection of CTCs in liquid biopsies. (A) Processes of label-free detection of CTCs from clinical peripheral blood using CytoExam. (B) CytoExam instrument highlighting the chip fixture (right, top) and liquid storage compartment (right, bottom). Scale bar, 5 cm. (C and D) eScan-chip, which is disposable and was designed with a lightweight lamellar structure. (E) Separation and concentration modules (i) and detection module (ii). Each module is filled with red ink for clear visualization. (F) Schematic of the mode of action of eScan-chip. (i) Inlet of the chip and outlets of the separation (ii), concentration (iii), and detection (iv) modules. (G) Block diagram of the control (black line), injection (blue line), and detection (red line) systems. (H) Custom software for instrument control.

Benefiting from the advantages of inertial microfluidics and impedance cytometry in label-free cell sorting and detection [37,38], as well as the huge potential of machine learning in cell discrimination [39,40], a microfluidic-based multistage strategy was designed to achieve label-free CTC detection with high throughput and accuracy, which is inspired by the works of well-known research groups in fields of inertial microfluidics and impedance cytometry [28,41]. Based on this strategy, CytoExam, a portable and automatic instrument that can rapidly and automatically process clinical peripheral blood samples undergoing simple red blood cell (RBC) removal steps, was developed (Fig. 1A). This new instrument can detect different cell types at different quantities in a sample using a pretrained cell discrimination algorithm based on deep learning. A series of relevant numerical simulations, as well as particle and cell experiments, were conducted to evaluate this cell detection strategy, and the clinical applicability of CytoExam was validated. Significant differences in the quantities of predicted WBCs and CTCs were observed between patients with breast or colorectal cancer and healthy donors. Thus, cells predicted using CytoExam may be potential indicators for the diagnosis and real-time monitoring of cancer.

## Results

### Device design and working principle

CytoExam was developed as an automatic and compact instrument for label-free CTC detection (Fig. 1B and Fig. S1), with overall dimensions of 50 × 40 × 40 cm (*L* × *W* × *H*). A multistage microfluidic chip (eScan-chip), mainly composed of separation, concentration, and detection modules, was clamped to the fixture on top of the instrument, and the adhesive layer, O-ring, and top and bottom covers of the chip were used for module connection and chip package (Fig. 1C to E and Fig. S2). The separation, concentration, and detection modules were designed as a spiral channel with a trapezoidal cross-section and 2-stage and asymmetric serpentine channels coupled with face-to-face electrodes (Fig. 1F).

The sample (containing WBCs and CTCs) was injected into eScan-chip through the inlet; CTCs were collected in the product outlet (PO), whereas WBCs and waste liquid were collected in waste outlets (WO) 1 to 5. This design allowed the removal of most WBCs and the enrichment of most CTCs and then improved the throughput and accuracy of CTC detection. Additionally, numerical simulations, previous experiments, and systematic research of famous scholars proved that the trapezoid design of the cross-section of the spiral channel offered a better separation accuracy than a rectangular cross-section (Fig. S3) [28,42], thereby offering a higher efficiency when separating WBCs and CTCs. Theoretically, cells flowing in these curved channels experience a combined action of inertial lift force (*F_L_*) and dean drag force (*F_D_*) [43,44], and gather at different positions in the curved channels according to their diameters [45]. Thus, the vast majority of WBCs and CTCs in the sample could be separated in the separation module (positions i and ii), with CTCs being concentrated in the concentration module (position iii) and then entering the detection module in an appropriate flow rate; ultimately, the CTCs could be focused on specific trajectories (position iv) to then be accurately detected via face-to-face electrodes placed in the detection module.

The instrument (Fig. 1G and Fig. S4) was composed of control, injection, and detection systems. Under the command of the control system, the instrument automatically completed the instrument washing, sample processing, and data recording functions (Figs. S5 and S6), with the entire process taking approximately 15 min. In addition, custom software was developed to assist in instrument control and display the sample processing steps (Fig. 1H).

### Experiments and numerical simulations verify the feasibility of channels

**Fig. 2.**
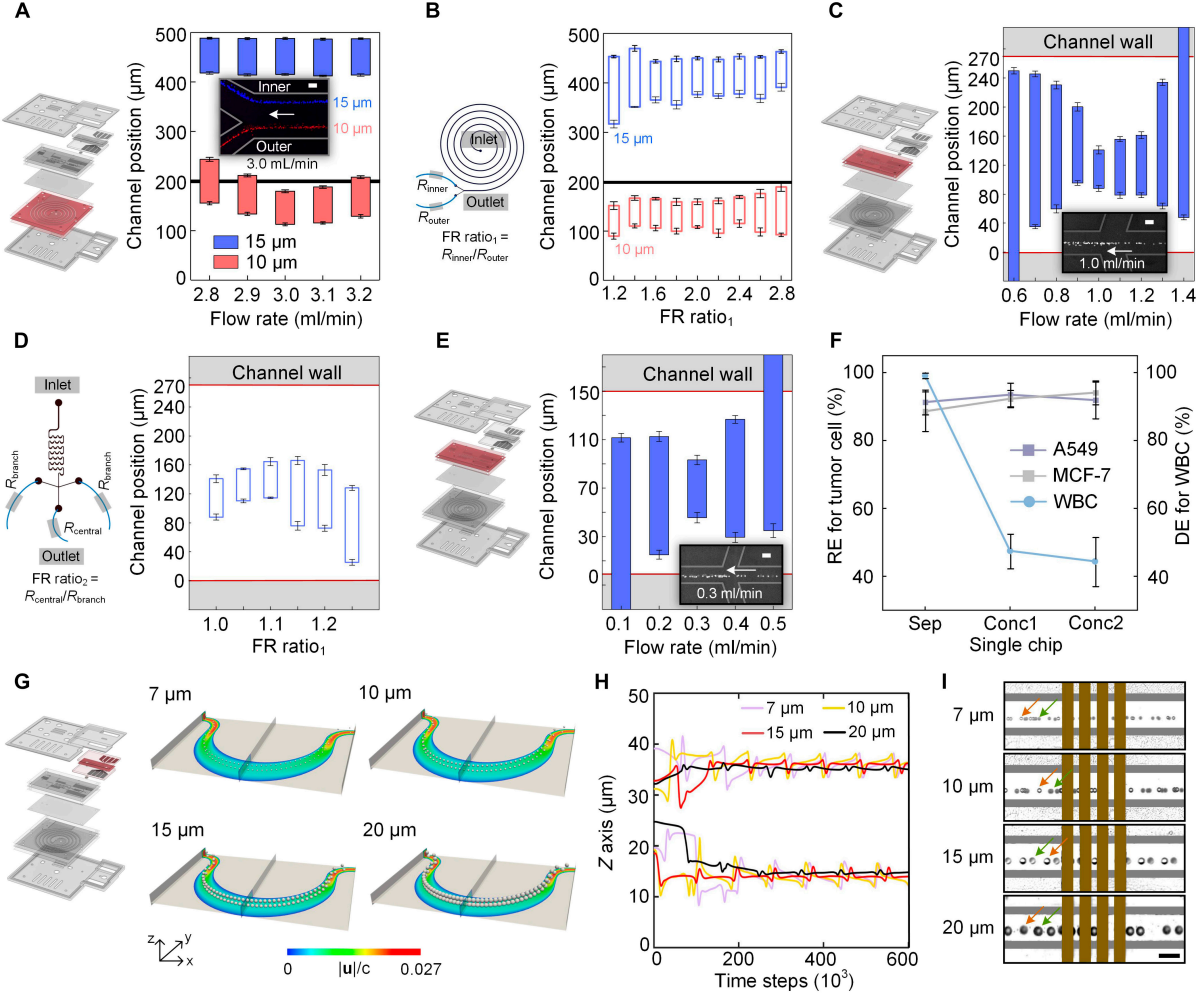
Schematics and characterization of each channel used in eScan-chip. (A and B) Influence of flow rate (A) and FR (B) on the particle focusing behaviors in the spiral channel with a trapezoidal cross-section. (C and D) Influence of flow rate (C) and FR (D) on the particle focusing behaviors in the first-stage serpentine channels. (E) Influence of flow rate on the particle focusing behaviors in the second-stage serpentine channel. (F) REs for tumor cells and DE for WBCs using the designed separation and first- and second-stage concentration channels. (G and H) Simulation data of particle migration trajectories (G) and vertical focusing trajectories (H) during the focusing process at a flow rate of 100 μl/min in the asymmetric serpentine channel. (I) Illustration of the particle vertical focusing trajectories. Particles marked with yellow and green arrows are focused on different planes. Scale bar, 50 μm.

Particle experiments were performed to explore the separation performance of the spiral channel of eScan-chip; 10- and 15-μm particles were used to simulate WBCs and CTCs. According to the basic theory of inertial microfluidics, the flow rate of fluids is vital for particle focusing. The separation between 10- and 15-μm particles was affected as the flow rate ranged from 2.8 to 3.2 ml/min, and an optimal separation between these 2 types of particles was achieved at a flow rate of 3.0 ml/min (Fig. 2A and Fig. S7). Additionally, the flow resistance ratio between the inner and outer outlets (FR ratio_1_) affects the particle focusing behavior. Under a flow rate of 3.0 ml/min, particle focusing and separation achieved the best output when the FR ratio_1_ was in the 1.4 to 2.2 range (Fig. 2B and Fig. S8). Considering that the lower inner outlet flow rate helps reduce the design limitations of the concentration and detection modules, the best FR ratio_1_ and the ideal flow rate for identifying CTCs or 15-μm particles were determined to be 2.0 and 1 ml/min, respectively.

To integrate the separation and detection modules, a 2-stage serpentine channel was designed. In the first stage, the serpentine channels with a height (*h*) of 100 μm and widths (*w*) of 300, 270, and 250 μm were tested (Fig. 2C and Fig. S9). With the increase of the flow rate, the focusing behavior of 15-μm particles becomes better in these channels, whereas the concentration effect was impaired as the flow rate was too high. Considering the sequential performance of the separation and concentration modules, the width of the first-stage serpentine channel was set at 270 μm, with an optimal flow rate of 1,000 μl/min. Moreover, the influence of the outlet flow resistance ratio (FR ratio_2_) on particle focusing and concentration fold was also explored (Fig. 2D and Fig. S10). To increase the concentration fold without impairing particle focusing, the FR ratio_2_ was set to 1.10 and the ideal flow rate of CTCs or 15-μm particles moving from the central outlet was determined to be 312.5 μl/min. According to the experiments in the first-stage serpentine channel, the optimal working flow rate of the serpentine channel increased with the channel width. Therefore, the width of the second-stage serpentine channel was set at 150 μm (Fig. 2E and Fig. S11), with the optimal flow rate of the inlet and outlet being approximately 300 and 100 μl/min, respectively.

To evaluate the cell-focusing performance of the spiral and serpentine channels, WBC or tumor cell samples (1 × 10^5^ cells/ml) were injected into the corresponding channels at optimal flow rates. The tumor cell recovery efficiency (RE) for the separation, as well as for the first- and second-stage concentration steps was approximately 90% (Fig. 2F), whereas the WBC depletion efficiency (DE) for the spiral channel was approximately 98%. Additionally, the influence of the cell concentration on the focusing behavior was also tested (Figs. S12 and S13). When the separation and 2-stage concentration steps were integrated, it worked well at a total cell concentration of 0.9 × 10^6^ cells/ml and the maximum cell throughput could reach approximately 46,000 cells/s.

Precise particle focusing in the asymmetric serpentine channel of the detection module is important for improving detection accuracy. A good focusing for 7- to 20-μm particles was achieved at flow rates of over 90 μl/min (Fig. S14); thus, the WBC and tumor cells that entered the asymmetric serpentine channel were expected to be well focused. As excessive flow rate reduces the signal-to-noise ratio of subsequent impedance signals, the optimal flow rate for the detection module was determined to be 90 to 100 μl/min, which met the requirements for integration with the upstream modules. Moreover, numerical simulation and experimental data demonstrated that there were 2 symmetric equilibrium trajectories in the cross-section of the channel when 7- to 20-μm particles were focused at this flow rate range (Fig. 2G to I). To achieve the precise detection of cells at any equilibrium trajectory, 4 pairs of face-to-face electrodes were set at the end of the asymmetric serpentine channel. Based on these 2 structures, the optimal cell concentration for the detection module to process the samples was determined to be <1.0 × 10^6^ cells/ml (Fig. S15), thereby meeting the module integration requirement.

### Module integration achieves WBC depletion and tumor cell enrichment

**Fig. 3.**
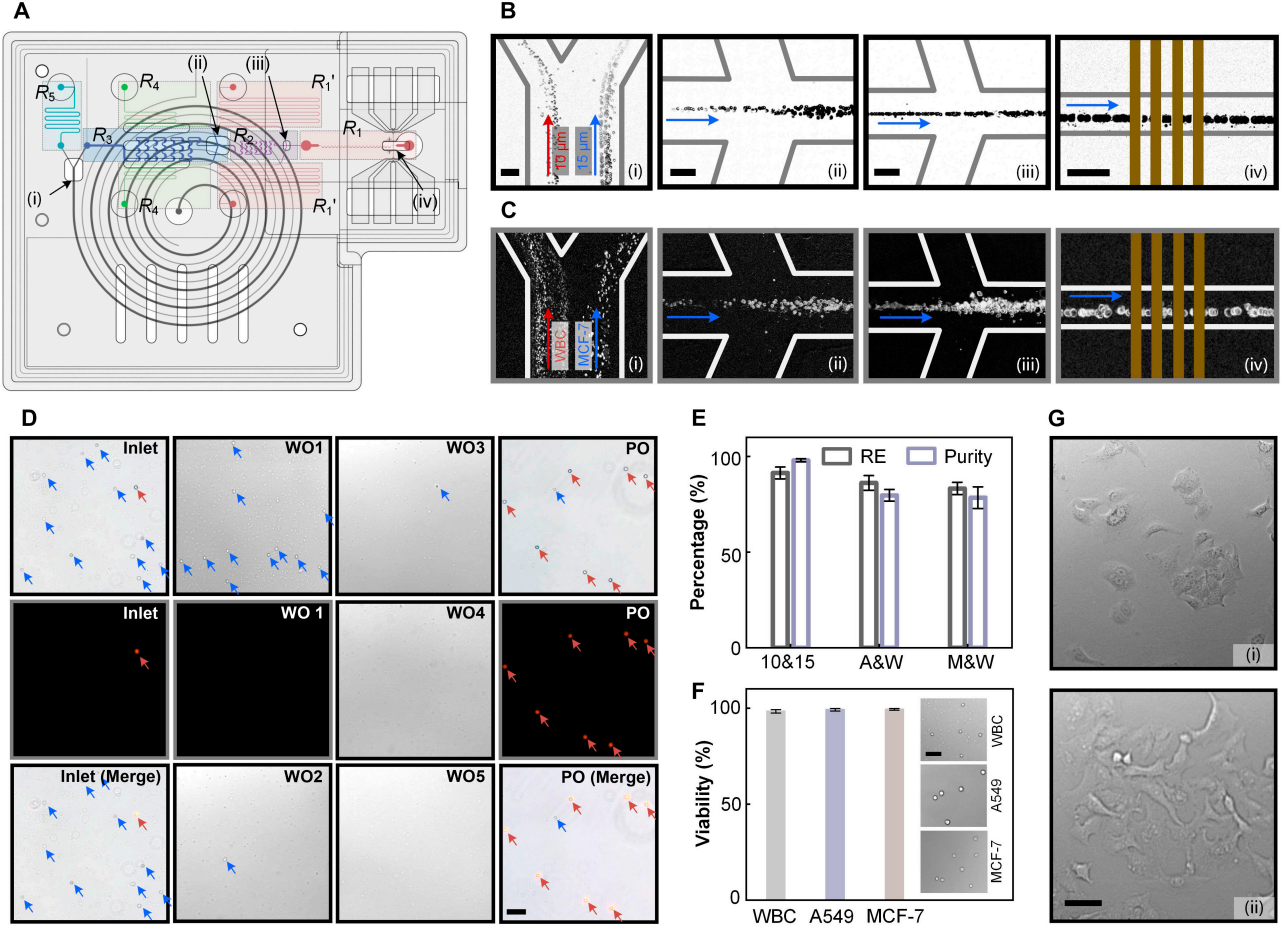
Characterization and performance of the 3 integrated modules of eScan-chip. (A) Schematic diagram of the integrated modules. Positions i to iv show the end of the channel in the separation, concentration, and detection modules. (B and C) Illustration of the distributions of the particles (B) and cells (C) at positions i to iv. (D) Brightfield and fluorescence microscopic images of samples collected from the inlet, WO1 to WO5, and PO of eScan-chip. Images with black background were captured using a fluorescence microscope. (E) REs and purity of the collected 15-μm particles, and A549 and MCF-7 tumor cells. (F) Viability of collected WBC, A549, and MCF-7 cells. The insets show cells after the trypan blue exclusion test. (G) MCF-7 cells recultured for 24 h (i) and 48 h (ii). All scale bars, 50 μm.

Flow resistance matching among the different channels of the separation, concentration, and detection modules was essential to maintain a good particle-focusing performance. Indeed, setting appropriate S-shaped channels (*R*_1_’, *R*_4_, *R*_5_) in the modules allowed to balance any changes in flow resistance during the integration process (*R*_1_, *R*_2_, *R*_3_) (Fig. 3A). Specific flow resistance calculations and matching processes were performed through numerical simulations and pressure-flow experiments (Fig. S16).

After complete integration, eScan-chip was evaluated through particle experiments to characterize its performance. According to the optimal flow rate of each channel, the mixed sample of 10- and 15-μm particles was injected into the inlet of eScan-chip at a flow rate of 3 ml/min. Overall, the 10- and 15-μm particles were separated with good efficiency in the separation module, and the 15-μm particles could then be concentrated in the concentration module and focused at specific trajectories in the detection module (Fig. 3B and Fig. S17). Considering the cells’ natural heterogeneity, a mixed sample of WBC (5 × 10^5^ cells/ml) and tumor cells (1 × 10^4^ cells/ml) was then applied to further evaluate the performance of eScan-chip (Fig. 3C and Fig. S18); the obtained results reflected excellent cell separation, concentration, and focusing performance. Furthermore, imaging analysis of the inlet, WO1 to WO5, and PO confirmed that most 15-μm particles or tumor cells were enriched and collected from the original samples, whereas most 10-μm particles or WBCs were depleted (Fig. 3D and Fig. S19). To quantify the performance of eScan-chip, REs and purities of the collected 15-μm particles and tumor cells were calculated. RE and purity of over 91% and 98% were achieved for 15-μm particles, whereas 2 types of tumor cells achieved REs and purities of over 83% and 78%, respectively (Fig. 3E). These results showcase the expected ideal performance of eScan-chip and suggest that it can be used to remove most of the useless background produced by WBC in clinical cancer samples, as well as to capture and concentrate CTCs (and some WBCs) for subsequent analysis.

Because eScan-chip achieves label-free separation, concentration, and detection of cells under microfluidic and microelectric fields, the collected tumor cells suffer less damage. A trypan blue exclusion test confirmed that over 98% of cells maintained good viability after passing the eScan-chip sorting process. Besides, in vitro culture of the collected tumor cells maintained a high 24-h multiplication rate (Fig. 3F and Fig. S20). Furthermore, successful reculture experiments of tumor cells in malignant pleural effusion also verified the superiority of eScan-chip in maintaining cell activity (Fig. S21).

### Unique electrode configuration realizes accurate cell detection

**Fig. 4.**
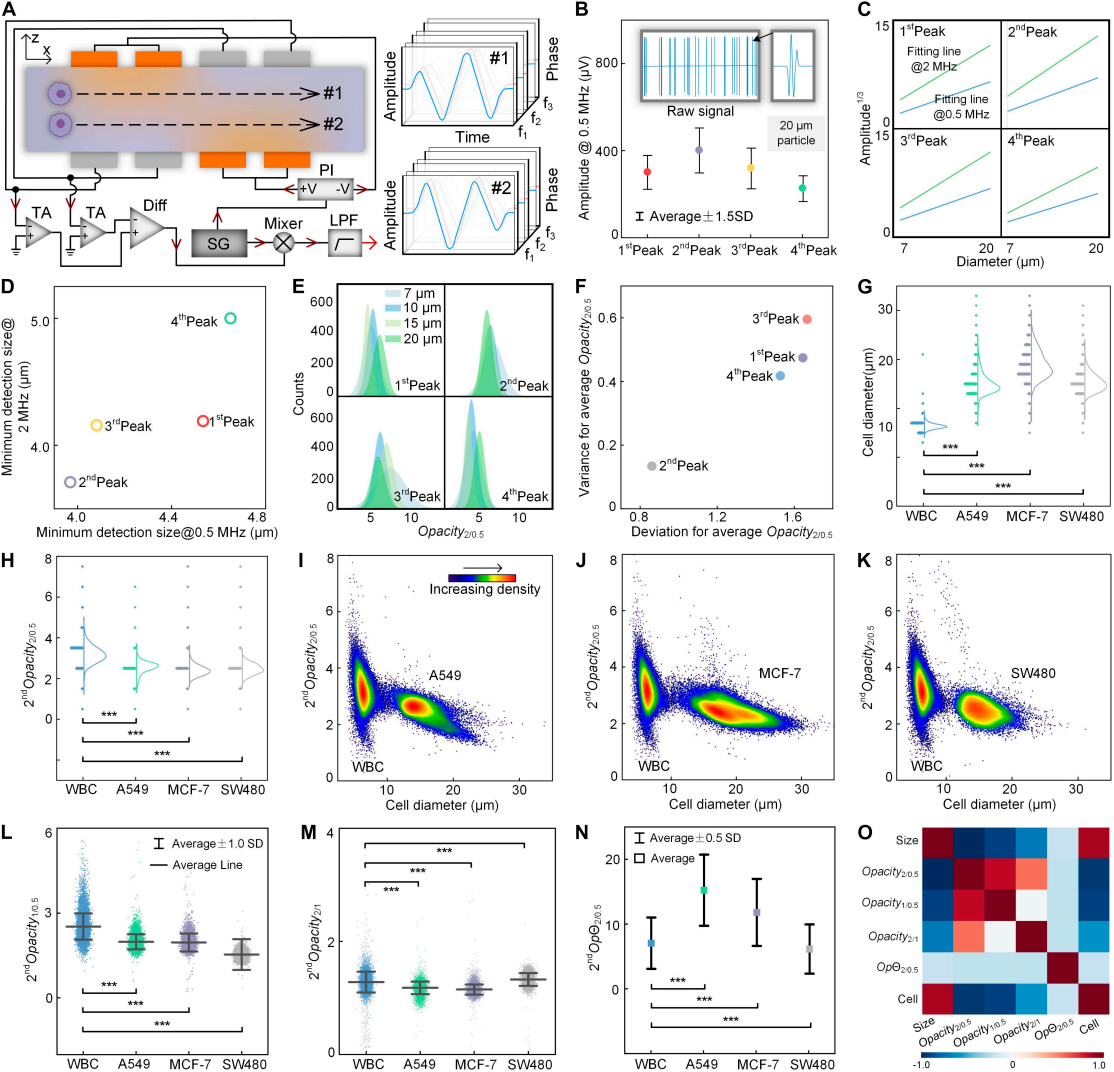
Electrode configuration design and characterization of impedance detection. (A) Schematic diagram illustrating the working principle of impedance detection. The same particles passing through trajectories #1 and #2 generate central symmetric impedance signals. (B) Raw signals and signal peak amplitude statistics of 20-μm particles at 0.5 MHz. (C) Linear relationship between signal peak amplitude^1/3^ and particle size at 0.5 and 2 MHz. (D) Minimum detection size of each signal peak at 0.5 and 2 MHz. (E) *Opacity*_2/0.5_ statistics for each type of particle and signal peak. (F) Evaluation of *Opacity*_2/0.5_ for each type of signal peak. (G and H) Comparison of cell diameter (G) and 2^nd^*Opacity*_2/0.5_ (H) distribution between different types of cells. (I to K) Density maps of cell diameter versus 2^nd^*Opacity*_2/0.5_ for A549 (I), MCF-7 (J), and SW480 tumor cells mixed with WBCs (K). (L to N) Comparison of 2^nd^*Opacity*_1/0.5_ (L), 2^nd^*Opacity*_2/1_ (M), and *Op*𝛩_2/0.5_ (N) distribution between different types of cells. (O) Correlation matrix of multiple electrical parameters between MCF-7 tumor cells and WBCs. ***P < 0.001, based on Student’s *t* test.

To achieve precise impedance detection of cells at 2 equilibrium trajectories during inertial focusing in an asymmetric serpentine channel, 4 pairs of face-to-face electrodes were placed using the 2-current differential method. Multi-frequency signals were generated by the signal generator (SG) and transported to the phase inverter (PI); 2 signals with the same voltage but a phase shift of 180° were then applied to the corresponding exciting electrodes (orange color) (Fig. 4A). When the focused cells passed through the electrode region, the corresponding current response signals were sensed in the sensing electrodes (gray color) due to the perturbation of the electric field by the cells. The sensed signals were amplified, differentiated twice, and demodulated at each frequency using a transimpedance amplifier (TA), differentiator (Diff), and lock-in amplifier composed of an SG, a mixer, and a low-pass filter (LPF). Finally, impedance signals were recorded and processed for cell detection and analysis. According to the numerical simulations and theoretical analysis (Figs. S22 and S23), the impedance signals of the cell showed 4 alternating peaks, whereas the signal shapes at 2 equilibrium trajectories (#1 and #2) exhibited equality and central symmetry. Therefore, cells focused at any equilibrium trajectory could be accurately detected using a detection module, effectively avoiding detection errors caused by positional deviations.

When particles passed through the detection region at a flow rate of 100 μl/min, raw signals of particles with the same diameter exhibited the same shape and size, giving rise to 4 alternating peaks, which was consistent with the aforementioned theoretical derivation (Fig. 4B and Fig. S24). In impedance cytometry, low (0.5 MHz) and high (1 to 2 MHz) electric field frequencies are commonly used to detect the size and internal dielectric properties of cells, respectively. Theoretically, the amplitude of impedance signals is proportional to the particle volume. Noteworthy, the particle diameter exhibited a linear relationship with each impedance peak amplitude (Fig. 4C). According to the signal-to-noise ratio of the impedance signals, the minimum detection size for all peak amplitudes was ≤5 μm and the minimum detection size for the second peak was the smallest at 0.5 and 2 MHz (3.97 and 3.71 μm, respectively), indicating that the detection module was capable of detecting WBCs and tumor cells. Generally, the internal dielectric properties of cells are characterized using opacity and *Op*𝛩 [25], with *Opacity*_h/l_ representing the ratio of impedance amplitudes between high and low frequencies (MHz) and *Op*𝛩_h/l_ representing the ratio of impedance phases between high and low frequencies (MHz). The average *Opacity*_2/0.5_ of the second peak had minimal variance and deviation when detecting polystyrene particles with the same material but different sizes (Fig. 4E and F), indicating that the second peak had the best stability for impedance detection.

Precise impedance sensing for cells was then performed (Fig. S25), and the diameter and *Opacity*_2/0.5_ of the cells were accurately calculated according to data obtained from the second peak of impedance signals (Fig. 4G and H). Generally, most tumor cells are larger than WBCs, whereas the *Opacity*_2/0.5_ of tumor cells is smaller than that of WBCs. Hence, density maps of WBC and tumor cells were plotted (Fig. 4I to K). Clear aggregation regions occurred for WBC and tumor cells, whereas a slight overlap between these 2 regions was observed, causing inaccurate cell discrimination. To further estimate the performance of cell impedance sensing, the other cellular electrical parameters, including *Opacity*_1/0.5_, *Opacity*_2/1_, and *Op*𝛩_2/0.5_, were extracted based on the second peak of the impedance signals (Fig. 4L to N), and significant differences between WBCs and tumor cells were observed. Moreover, the correlation of multiple electrical parameters between WBC and tumor cells was also analyzed (Fig. 4O and Fig. S26). All the tested parameters are confirmed to be independent of each other and to reflect unique cellular dielectric properties, thereby verifying the feasibility of the detection module for cell detection.

### Performance with clinical samples

**Fig. 5.**
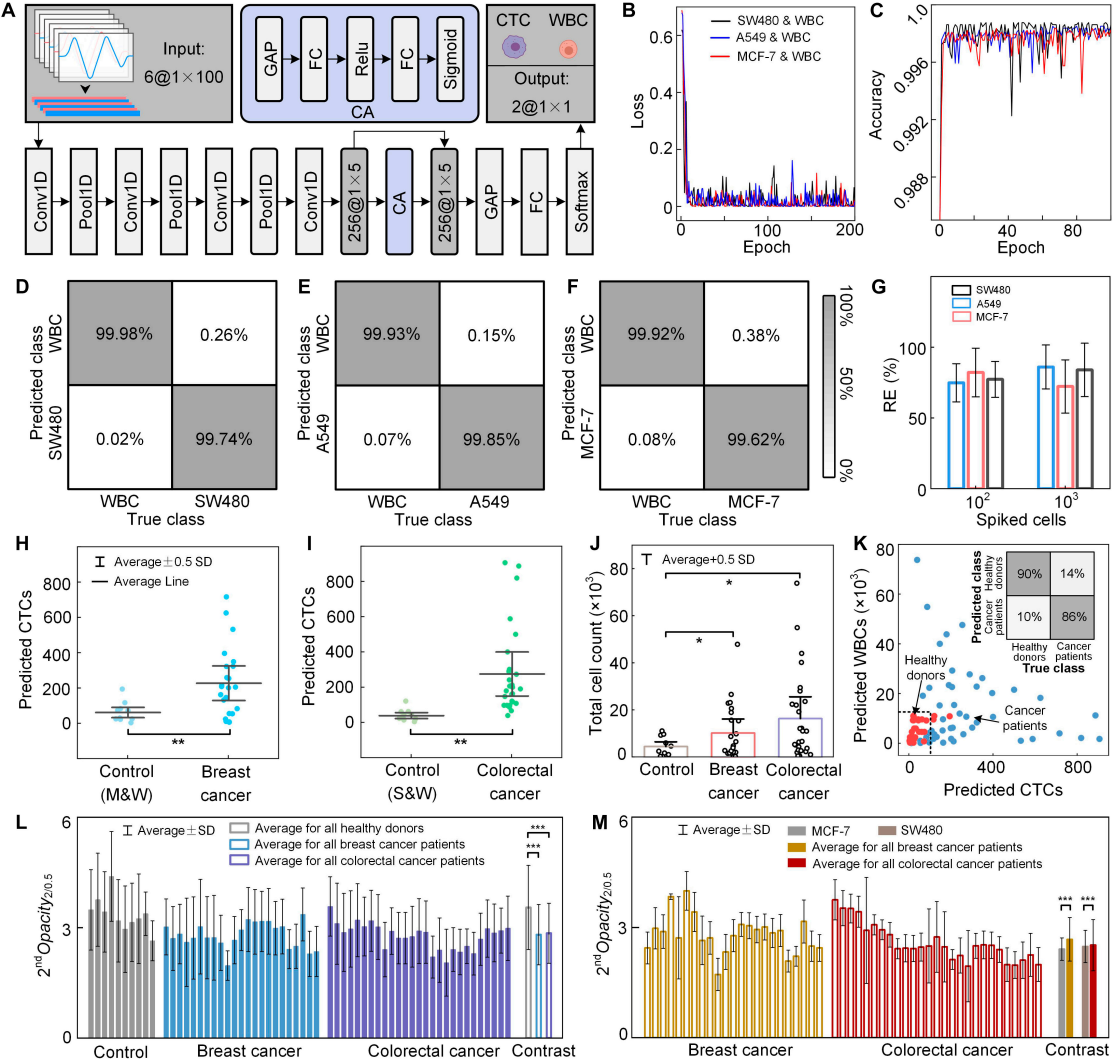
Clinical application and data analysis. (A) Architecture of the cell discrimination algorithm developed based on 1D CNN. (B and C) Loss curves (B) for training sets and accuracy curves (C) for testing sets during the training process. (D to F) Confusion matrixes of cell discrimination results for SW480 (D), A549 (E), and MCF-7 (F) tumor cells mixed with WBCs. (G) REs for detecting tumor cells spiked in samples with high WBC concentration. (H and I) Comparison of predicted CTCs between healthy donors (*n* = 10) and patients with breast (*n* = 23) (H) and colorectal (*n* = 27) (I) cancers. (J) Comparison of total detected cells between healthy donors (*n* = 20, where the 20 data points were obtained through CytoNets pretrained by M&W and S&W datasets) and breast (*n* = 23) and colorectal (*n* = 27) cancer patients. (K) Scatter plot of predicted CTCs versus predicted WBCs for healthy and cancer samples. The inset illustrates the confusion matrix of cancer diagnosis results. (L) Comparison of the 2^nd^*Opacity*_2/0.5_ distribution between healthy donors and cancer patients. (M) Comparison of the 2^nd^*Opacity*_2/0.5_ distribution between tumor cell lines and the CTCs predicted in cancer patients. *P < 0.05, **P < 0.01, and ***P < 0.001, based on Student’s *t* test.

Due to the scarcity of CTCs circulating in the blood of patients with cancer, precise discrimination between WBCs and CTCs based on their diameter and opacity is challenging. Therefore, a cell discrimination algorithm based on deep learning one-dimensional (1D) convolutional neural network (1D CNN), named CytoNet, was developed. Compared to 2D CNN, which is commonly used for processing image data, 1D CNN is more suitable for processing sequence data containing temporal information, such as electrical signals [46]. CNN usage helps extract hidden features of cells, which may benefit cell discrimination. CytoNet comprised input layer, 4 convolutional (Conv1D) layers, 3 pooling (Pool1D) layers, a global average pooling (GAP) layer, a fully connected (FC) layer, and a classification (softmax) layer (Fig. 5A). In addition, a channel attention (CA) mechanism was imported into this network before the GAP layer. This mechanism aimed to assign weights to different feature channels and strengthen key feature channels, thereby further increasing discrimination accuracy. Six signal responses, including the amplitude and phase for each frequency, were generated as the electric field containing 3 frequencies of 0.5, 1, and 2, and the response signals were processed as tensor data with a size of 1 × 100 × 6 before being fed into the network.

For application to other cancers, 3 groups of mixed tumor cell:WBC (1:1 ratio) datasets, including the SW480 (S&W), A549 (A&W), and MCF-7 (M&W) tumor cells, were created. After training for approximately 100 epochs, the loss for the training sets and the accuracy for the testing sets tended to converge and stabilize at an excellent level (Fig. 5B and C). Noteworthily, the true positive rates (TPRs) for WBC and tumor cells in each dataset were over 99.9% and 99.6%, respectively (Fig. 5D to F), illustrating a significant enhancement compared with the discrimination method that extracts cell features subjectively (Fig. S27). Furthermore, using the pretrained neural network, eScan-chip was able to successfully predict the number of tumor cells in WBC suspensions (with a volume of 20 ml and a concentration of 5 × 10^5^ cells/ml) that were spiked with tumor cells (10^2^ and 10^3^ orders of magnitude) (Fig. 5G).

Last, the clinical applicability of CytoExam was validated. In total, 50 and 10 clinical blood samples were collected from patients with breast or colorectal cancer and healthy donors. Each sample contained 1 ml of blood; RBCs were removed, and the residual cells were suspended in 20 ml of phosphate-buffered saline (PBS) before being injected into the instrument or chip. The results of the healthy donors were predicted using CytoNet upon training with each dataset (Fig. S28), whereas the predicted results of cancer patients were obtained using CytoNet upon training with the corresponding datasets (Fig. 5H and I and Fig. S29). A significant difference was observed in the number of predicted CTCs between patients and healthy donors. Significant differences were also observed in the number of total cells and predicted WBCs between patients and healthy donors (Fig. 5J and K), indicating that cancer may cause an increase in the frequency and volume of WBCs in patients. By allocating appropriate quantity thresholds for the predicted cells, the accuracy of cancer diagnosis was calculated to be 87.5%. Moreover, the cellular dielectric properties of cells from healthy donors and cancer patients, as well as of tumor cell lines and CTCs predicted in cancer patients were analyzed (Fig. 5L and M). Generally, healthy donors exhibited larger 2^nd^*Opacity*_2/0.5_ than cancer patients, indicating that the cells collected from cancer patients had larger capacitive reactance than those of healthy donors. CTCs predicted in cancer patients exhibited larger 2^nd^*Opacity*_2/0.5_ than tumor cell lines, indicating that the CTCs predicted in cancer patients had smaller capacitive reactance than the cells from tumor cell lines. Additionally, to verify the multi-applicability of our instrument for cancer, clinical blood samples from lung cancer and pleural effusion from breast cancer patients were also detected successfully (Figs. S30 and S31). Related clinical and pathological data of all patients can be found in Table S1. Overall, the aforementioned results verify the feasibility of our instrument for cancer diagnosis and research on cellular dielectric properties.

## Discussion

It has been emphasized that label-free detection of rare CTCs is urgently needed for the clinical diagnosis, real-time monitoring, and prognosis of patients with cancer. Here, CytoExam, a liquid biopsy instrument for the rapid, automatic, and label-free detection of rare tumor cells, is described. Peripheral blood samples subjected to RBC removal were processed within 15 min using CytoExam, and information on cell types, quantities, and dielectric properties was obtained by analyzing impedance signals using a pretrained neural network.

Compared with the commonly used immune affinity-based method for CTC detection (Table S2), CytoExam has many significant advantages. In terms of cost, no expensive biochemical reagents (e.g., antibody or magnetic beads) were used, but a cheap eScan-chip was designed. Therefore, the material cost and time consumption for each sample were only approximately ^1^/_40_ and ^1^/_60_ of the immune-affinity-based methods, respectively, which indicates significant application values and enormous commercialization prospects. In terms of cell viability, compared with dead cells after immunostaining, the cells processed using CytoExam maintained a high viability, holding great potential for further single-cell analysis. Immune affinity-based methods classify cells by screening each image according to the experience of the expert technician, being susceptible to misjudgment and poor detection stability, and requiring labor-intensive operations. In contrast, automatic, ultra-fast, and efficient cell classification was achieved in our work using CytoExam.

After conducting the relevant numerical and experimental verifications, the clinical applicability of CytoExam was evaluated. Significant differences in the number of predicted WBCs and CTCs between breast and colorectal cancer patients and healthy donors were observed. Even if most WBCs were removed from the separation module, more predicted WBCs could still be obtained from patients than from healthy donors. According to the difference in the predicted cells, the discrimination accuracy between patients and healthy individuals reached 87.5% by allocating an appropriate quantity threshold. Compared with the results reported in some previous studies, an excessive quantity of predicted tumor cells was achieved in this study [28]. It is assumed that there are differences in some of the hidden features between cell lines and clinical samples, even for the same cell type, thereby causing cell discrimination misjudgment of pretrained neural networks. Importantly, cells collected from cancer patients showed to have greater capacitive reactance than healthy donor cells, whereas the CTCs predicted in cancer patients exhibited smaller capacitive reactance than tumor cell lines. These findings provide new physical indicators for clinically analyzing cells obtained from patients with cancer.

Since the label-free manipulation and detection of cells using CytoExam were achieved through the physical features of the cells, the applicability of this instrument is expected to be extended to more types of cancers as compared with the traditional cell-specific staining-based immune-affinity method. By accumulating more clinical data on various types of cancers and further training cell discrimination algorithms based on these data, a more accurate cancer diagnosis is expected. In conclusion, unlike existing instruments that detect tumor cells using expensive, time-consuming, and labor-intensive immune-affinity methods, CytoExam offers a fast, automatic, affordable, and label-free approach. It is envisioned that the use of this instrument will open avenues for rare tumor cell research in the life sciences and clinical cancer studies.

## Methods

### CytoExam construction

CytoExam appearance and structure were designed using SOLIDWORKS 2018 software. The bearing and connecting parts inside the instrument were made of machined aluminum alloy and 3D-printed nylon, respectively, whereas the shells of the instrument were made of 3D-printed photosensitive resin. Electrical components inside the instrument belong to the injection, detection, and control systems. The injection system was composed of a syringe pump equipped with a 6-way switch valve (Pump XLP 6 K, TECAN), a bubble sensor (OCB350L, ALEPH), 2 cutoff valves (XIN0502SV, Xinxin Electronics), and a 3-way switch valve (Mrv 01B, Runze Fluid). The detection system was composed of an impedance spectroscope (HF2IS, Zurich Instruments), TA (HF2TA, Zurich Instruments), pulse inverter (INV-0026, Marki Microwave), and a customed small mainframe. In addition, a customized mainframe, touch screen (DM101, TouchWo), and customized microcontrollers with an advanced RISC (reduced instruction set computer) machines (ARM) program in the control system were used to achieve electrical component control and parameter setting.

#### eScan-chip fabrication

eScan-chip consisted of separation, concentration, and detection modules. A film-chip manufacturing technology that was previously developed was used to fabricate the separation and concentration modules (Fig. S32) [42]. Briefly, silicon and polymer films were cut using an ultraviolet laser machine (TH-UV2000A, Tianhong Laser) to fabricate the channels and covers, and the channels were then enclosed by bonding corresponding coves on both sides through plasma treatment using a plasma cleaner (PDC-002, Harrick Plasma). Notably, when both sides of the channel were fabricated using silicon films with different thicknesses, a spiral channel with a trapezoidal cross section in the separation module could be easily fabricated. Additionally, this unique technology was used to fabricate impedance cytometry using face-to-face electrodes. The channel was cut from silicon film using a laser, while the electrodes were fabricated from gold-plated glasses using photolithography (MJB4, SUSS MicroTec) (Fig. S33). The fabricated gold-plated glasses were then bonded to both sides of the channel through plasma treatment. Specific images of the fabricated separation, concentration, and detection modules are shown in Fig. S34.

#### Sample preparation

To characterize the performances of the separation, concentration, and detection modules, polystyrene microspheres (7 to 20 μm, Thermo Fisher Scientific) and commercially available human breast cell (MCF-7), non-small cell (A549), and colorectal adenocarcinoma (SW480) cell lines were used. Polystyrene microspheres with 1% solid content were diluted to an appropriate particle concentration using PBS (Sigma-Aldrich). Cancer cell lines were cultured in a carbon dioxide incubator (Forma 381, Thermo Fisher Scientific) in high-glucose Dulbecco’s modified Eagle’s medium (Thermo Fisher Scientific) mixed with 10% fetal bovine serum (Thermo Fisher Scientific) and 1% penicillin-streptomycin (Thermo Fisher Scientific). Before the cell experiments, the cultured cancer cells were dissociated using a 0.25% trypsin-EDTA solution (Thermo Fisher Scientific) and resuspended in PBS at appropriate concentrations. To determine the cell types in the mixed cell samples, target cells were prestained using a calcein-AM solution (Thermo Fisher Scientific).

Clinical blood samples from patients and healthy donors were collected into vacutainer collection tubes (BD Biosciences) containing K_2_EDTA. Before use, RBCs in the samples were lysed using ammonium chloride-potassium lysis buffer (Thermo Fisher Scientific) and were then removed using standard centrifugation steps. This study was approved by the institutional committee of the Institutional Ethical Committee (IEC) for Clinical Research of Zhongda Hospital of Southeast University (2020ZDSYLL043), and informed consent was obtained from all volunteers. All experiments were performed in compliance with respective government laws and institutional guidelines.

#### Experimental setup

In the inertial microfluidic experiments, a syringe pump (Legato 100, KD Scientific) was employed to provide stable and accurate fluid injection at various flow rates. The related modules were placed on an inverted microscope (IX 71, Olympus) equipped with a charge-coupled device (CCD) camera (Exi Blue, Qimaging) to capture images of the particle motion status. An upright fluorescence microscope (80i, Nikon) equipped with a color camera (DS-Ri1, Nikon) was used to capture the stained cells under fluorescent light illumination. All captured images were processed using ImageJ software (https://imagej.net/ij/). In the impedance detection experiments, the electric field voltages for each frequency were set to 3.3 V through the impedance spectroscope, the bandwidth of the modulator was set to 1% for each frequency, the sample rate for each frequency was set to 115 kSa/s, and the gain of the trans-impedance amplifier was set to 10^3^ V/A.

The automatic operation of CytoExam included 4 steps: precleaning steps 1 and 2, sample injection, and postcleaning steps (Fig. S35). Precleaning steps 1 and 2 promoted bubble removal and tube cleaning in the instrument through ethanol and PBS, respectively. The sample injection step is aimed at pumping the sample at a preset flow rate. The postcleaning step achieved the removal of residual cells in the tubes of the instrument. The operational process of the instrument is shown in Movie S1.

#### Impedance data processing

The recorded impedance signal data were processed using algorithms written in Python (version 3.9). Briefly, raw signals were detrended and filtered to improve the signal-to-noise ratios, and the peaks and complete signal fragments of each cell were extracted and stored. The peak amplitude and phase could be easily obtained for the subsequent calculation of the cell diameter and opacity. Complete signal fragments of target cells were stored as groups of datasets to train and test the neural network, and each group of datasets was partitioned into training and testing sets in a 7:3 ratio. An i7 9700 CPU (Intel Core)-equipped processor and a GTX 1660Ti GPU (NVIDIA GeForce)-equipped processor were used for data analysis and neural network training.

#### Numerical simulation

Numerical simulation of the flow field in spiral channels with rectangular and trapezoidal cross-sections was performed by solving the steady continuity, Navier–Stokes equation, and convection–diffusion equations using COMSOL Multiphysics 5.6 software. The fluid dynamic boundary conditions were set as no-slip at all channel walls, and the pressure at the channel outlet was set to 0 Pa. A numerical simulation of the impedance signal response was performed using the COMSOL software. Notably, the boundary conditions of the detection region were defined using an equivalent circuit model, in which a single-shell model was used to mimic the cell, the medium of the flow field was set as PBS, and the electric field was set as alternating current.

The inertial focusing behavior of the cells in an asymmetric serpentine channel was simulated using the lattice Boltzmann method (LBM), finite element method (FEM), and immersion boundary method (IBM). Compared to the numerical simulation using COMSOL software, these methods can simulate particle migration and focusing behavior in microchannel more precisely. The LBM was used to evolve the flow field in the channel, the FEM was used to build finite element models of cells, the IBM was used to transfer the velocity on the fluid node to the particle node, and the restoring force on the particle node was transmitted back to the fluid node, ultimately achieving the interaction between the particles and fluid. A detailed discussion of the numerical simulation can be found in a previous report [47].

#### Statistical analysis

Statistical comparisons of different cell types were performed using Student’s *t* test, with *P* < 0.05 deemed statistically significant. The experimental *n* that appeared in Figs. 4 and 5 was all over 5,000 unless stated otherwise.
