# Supplementary material for: Liquid Biopsy Instrument for Ultra-Fast and Label-Free Detection of Circulating Tumor Cells
Source: Research (Wash D C). 2024 Jul 24;7:0431. doi: 10.34133/research.0431 (PMC11266806; doi:10.34133/research.0431)
Supplement: Supplementary 1 — Figs. S1 to S35 Table S1 and S2 [file research.0431.f1.zip › Supporting Information.docx]

**Supporting information**

**Liquid Biopsy Instrument for Ultra-fast and Label-free Detection of Circulating Tumor Cells**

**Shu Zhu,^1,2^ Zhixian Zhu,^1,2^ Chen Ni,^1,2^ Zheng Zhou,^1^ Yao Chen,^1^ Dezhi Tang,^1^ Kefan Guo,^1^ Shuai Yang,^1^ Kang Liu,^1^ Zhonghua Ni^1^*****, and Nan Xiang^1^***

*1. School of Mechanical Engineering, and Jiangsu Key Laboratory for Design and Manufacture of Micro-Nano Biomedical Instruments, Southeast University, Nanjing, 211189, China.*

*2. These authors contributed equally to this work.*

**E-mails: nzh2003@seu.edu.cn; nan.xiang@seu.edu.cn.*


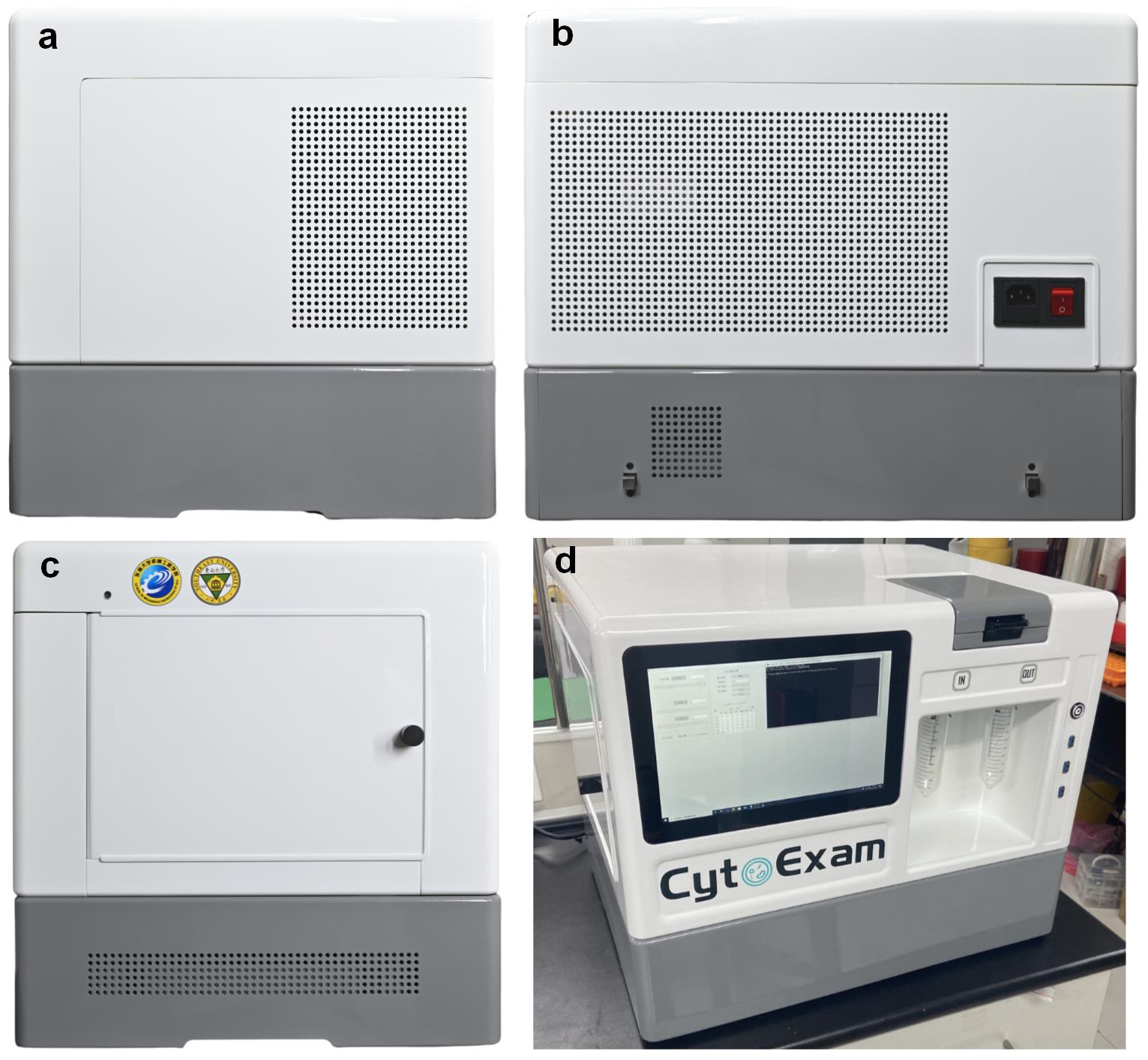


**Fig. S1: Images from different perspectives of the instrument.** **a,** Right view. **b,** Back view. **c,** Left view. **d,** Image of the instrument after turning on.


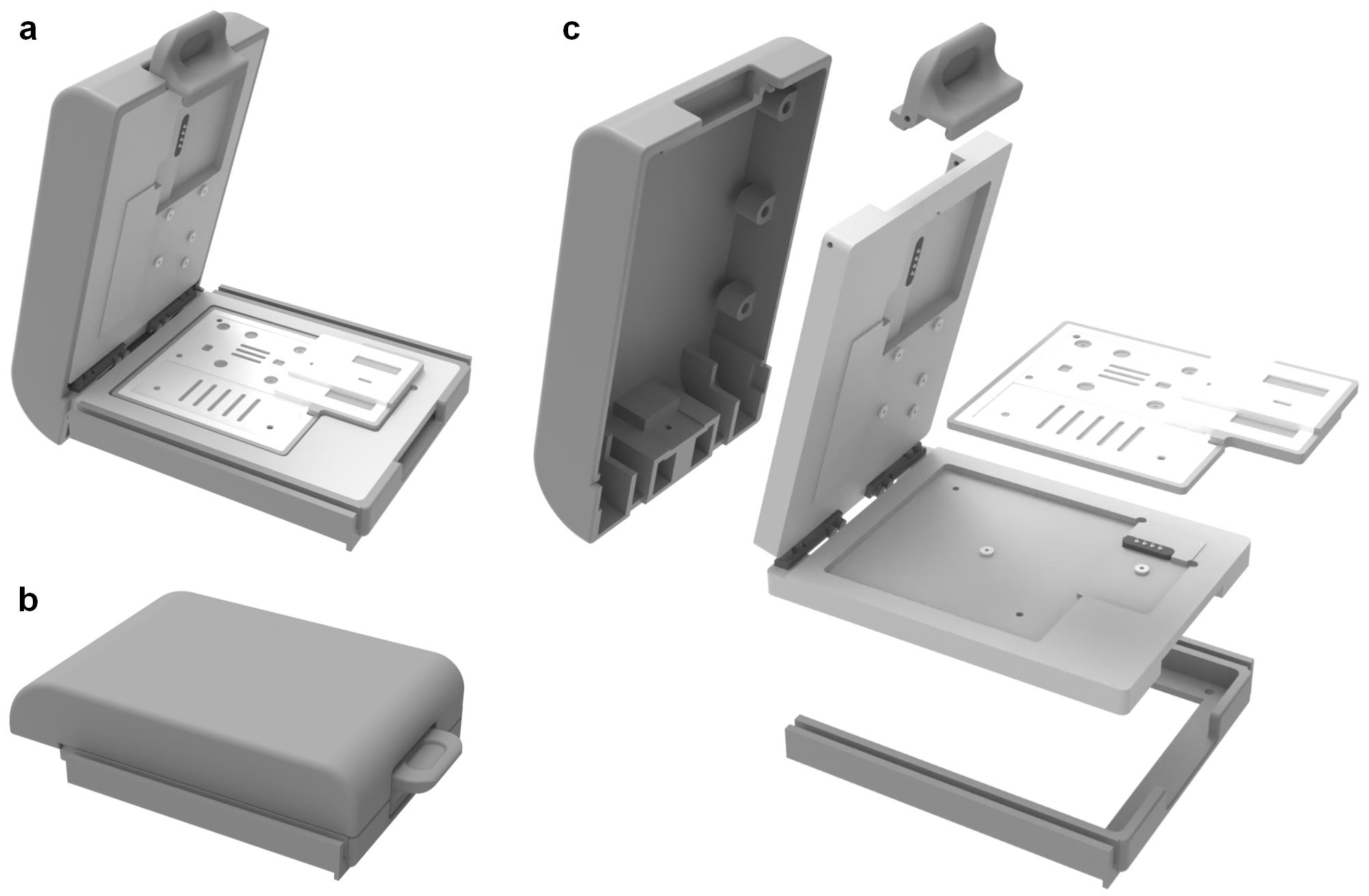


**Fig. S2: Illustration of the chip fixture. a,** Open status of the chip fixture. **a,** Close status of the chip fixture. **c,** Explosive view of the chip fixture.


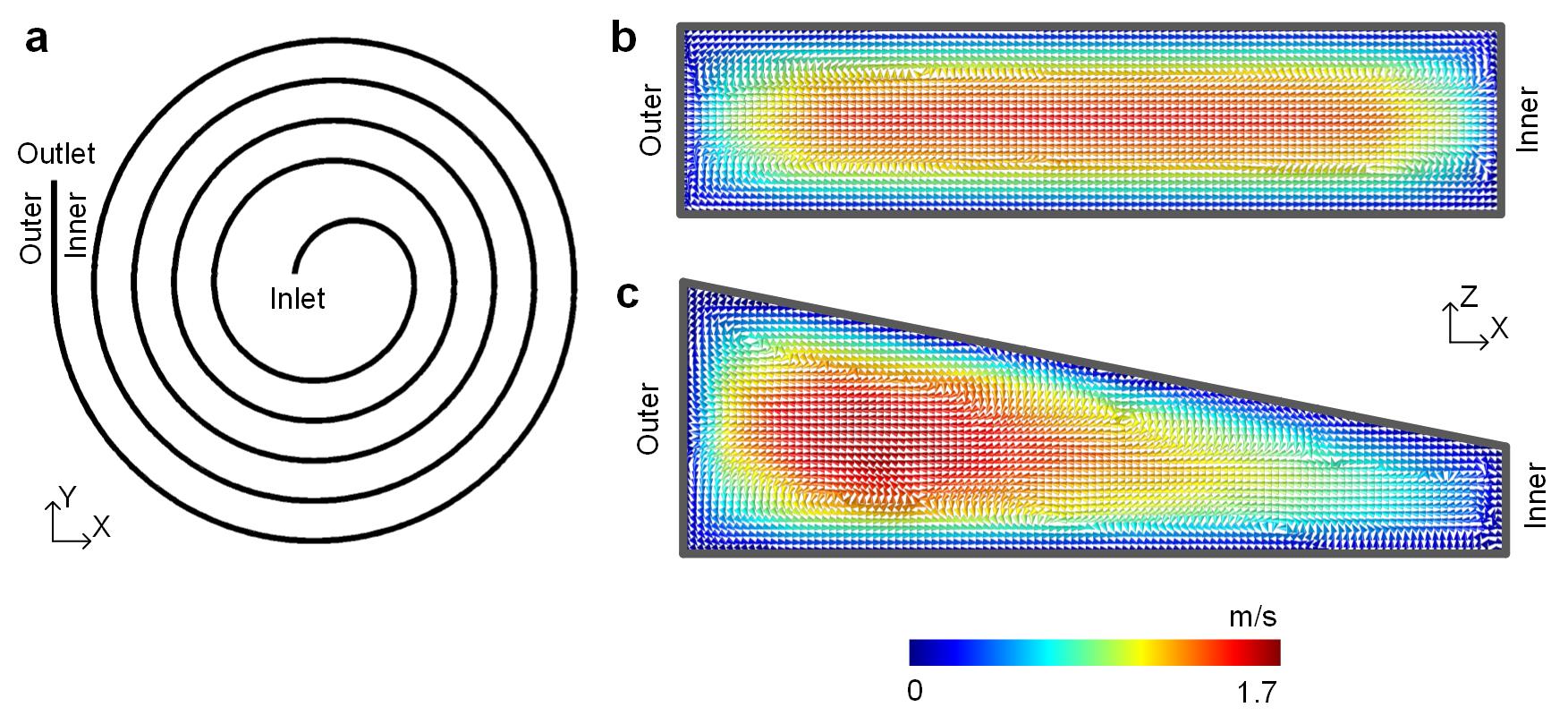


**Fig. S3: Cross-sectional flow field distribution of the spiral channel in a flow rate of 3 mL/min. a,** Structure and dimension of the spiral channel in the XY plane. **b,** Cross-sectional flow field distribution in spiral channel with rectangle cross-section. **c,** Cross-sectional flow field distribution in spiral channel with trapezoid cross-section. Simulation results display the core of the Dean vortex in the rectangle and trapezoid cross-section clearly (highlighted in red), and the Dean vortex in trapezoid cross-section is closer to the outer wall of the channel.^1^ In the spiral channel with rectangle cross-section, under the competence of inertial lift force *F*_L_ and Dean drag force *F*_D_,^2^ large particles tend to be balanced and focused near the inner wall of the channel,^3^ while small particles move with Dean vortex at the center of the channel.^4^ Therefore, the distance between large and small particles is the key to determining the capability of particle separation. For the spiral channel with trapezoid cross-section, as the Dean vortex moving towards the outer wall of the channel, the distance between large and small particles is enlarged subsequently, thereby causing high particle separation accuracy than that of spiral channel with rectangle cross-section.


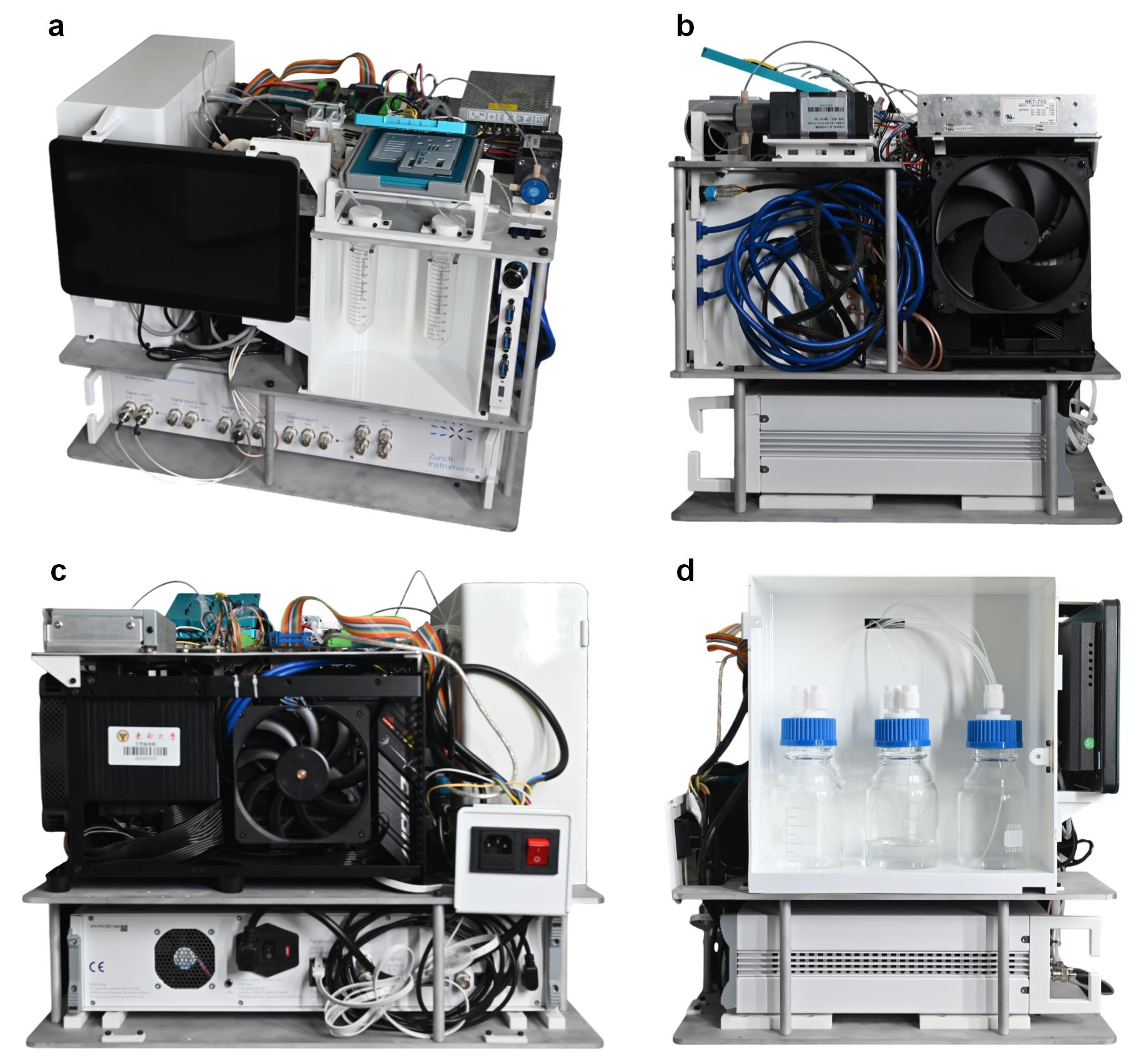


**Fig. S4: Internal photographs of the instrument. a,** Front view. **b,** Right view. **c,** Back view. **d,** Left view.


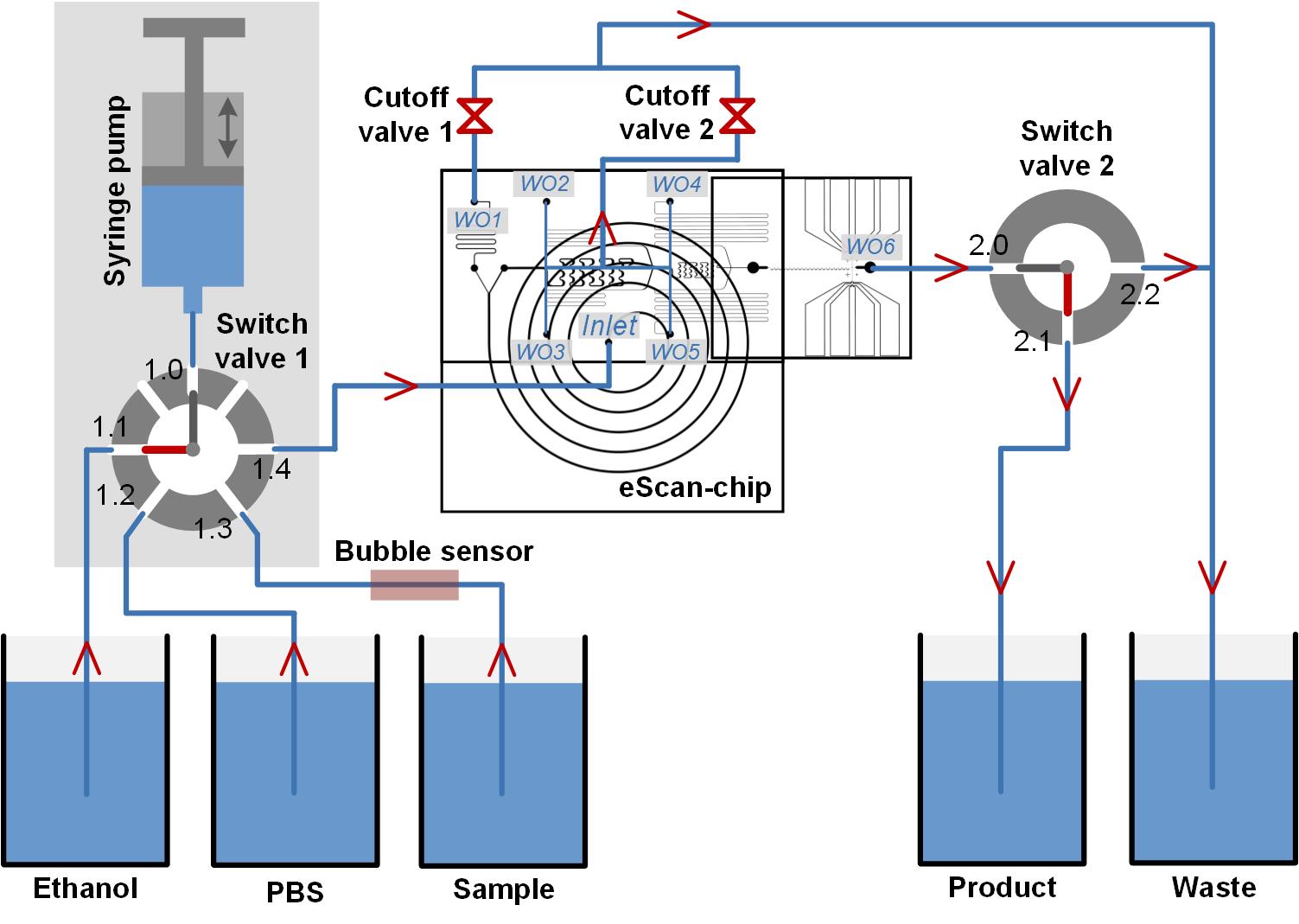


**Fig. S5:** **Schematic diagram of the injection system.** Four steps are performed using this system, containing pre-cleaning 1 and 2, sample injecting, and post-cleaning steps. Two cutoff valves are set open when performing the above steps, and they are set close as the above steps finished. In pre-cleaning 1 step, the switch valve 1 is in the position 1.1, the switch valve 2 is in the position 2.2; and the syringe pump extracting ethanol at this time. The switch valve 1 then turn to the position 1.4, and the syringe pump injects the ethanol out. In pre-cleaning 2 step, the switch valve 1 turn to the position 1.2, the switch valve 2 is in the position 2.2; and the syringe pump extracting PBS at this time. The switch valve 1 then turn to the position 1.4, and the syringe pump injects the PBS out. In sample injecting step, the switch valve 1 is in the position 1.3, the switch valve 2 turn to the position 2.1; and the syringe pump extracting sample at this time. When the sample is drawn out, the bubble sensor senses the bubble in tube and generates stop command to the syringe pump. The switch valve 1 then turn to the position 1.4, and the syringe pump injects the sample out. In post-cleaning step, the switch valve 1 turn to the position 1.2, the switch valve 2 turn to the position 2.1; and the syringe pump extracting PBS at this time. The switch valve 1 then turn to the position 1.4, and the syringe pump injects the PBS out.


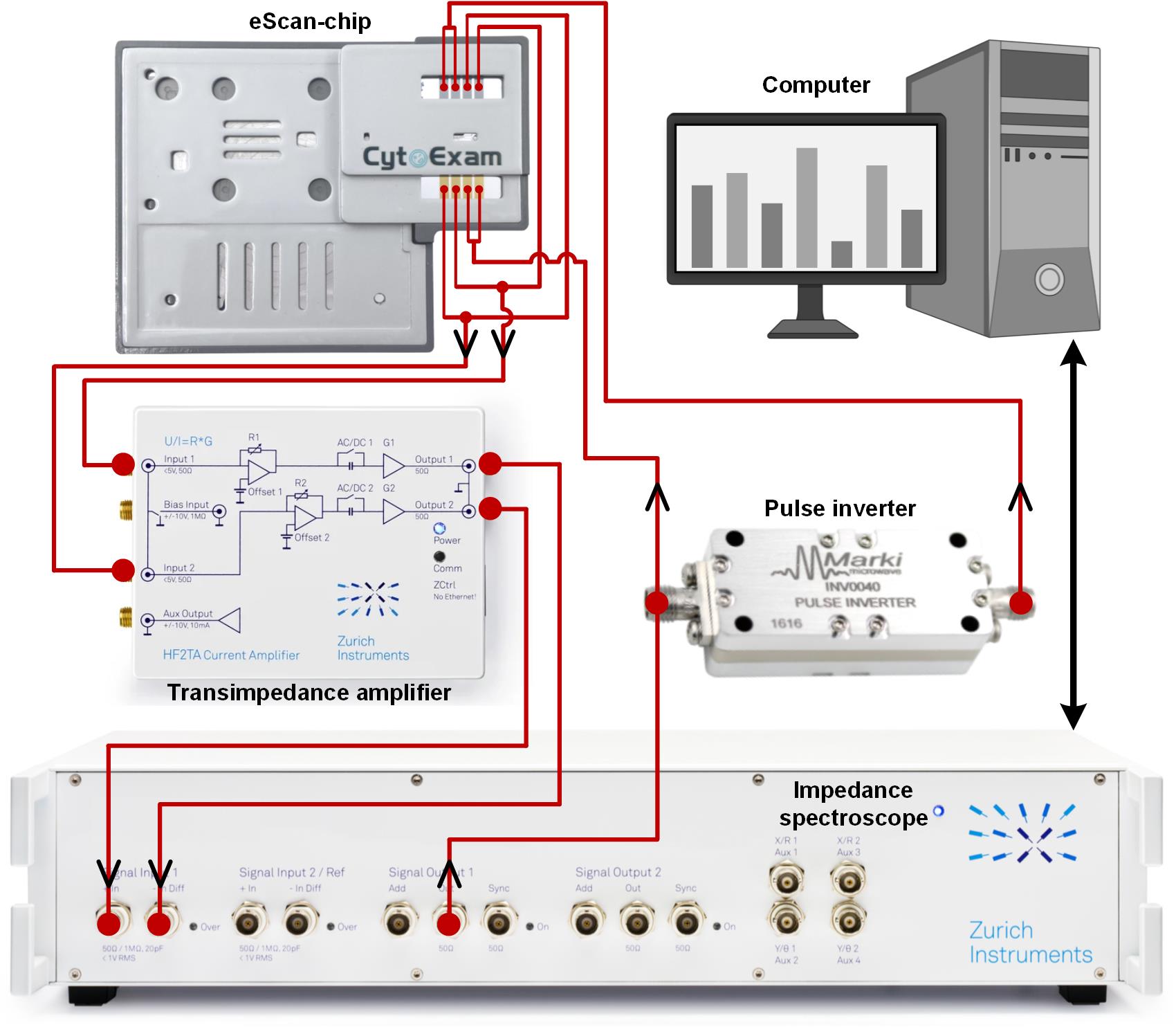


**Fig. S6: Schematic diagram of the detection system.** Red line represents the communication of detection signal between different component; Black line represents the communication of control and data transmission signal between the computer and the impedance spectroscope.


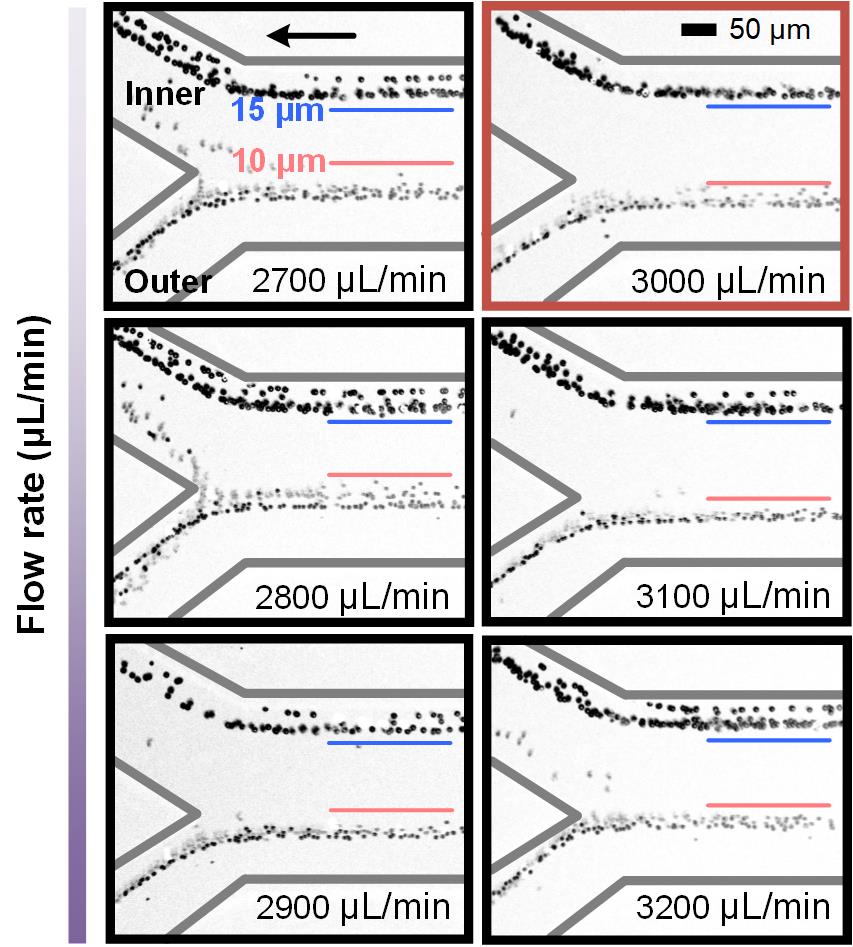


**Fig. S7: Characterization of the spiral channel with trapezoid cross-section.** Images illustrating the particle distributions at different flow rate in the spiral channel with trapezoid cross-section.


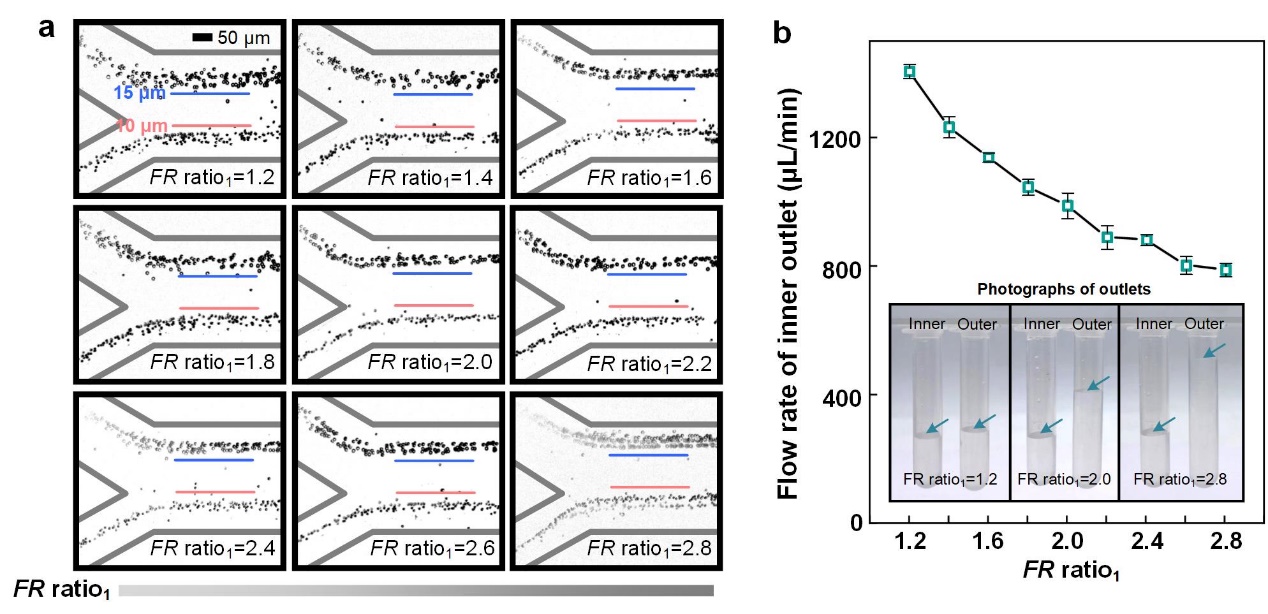


**Fig. S8: Characterization of the spiral channel with trapezoid cross-section. a,** Images illustrating the influence of flow resistance (*FR*) on particle focusing behavior. **b,** The influence of *FR* on the flow rate of the inner outlet.


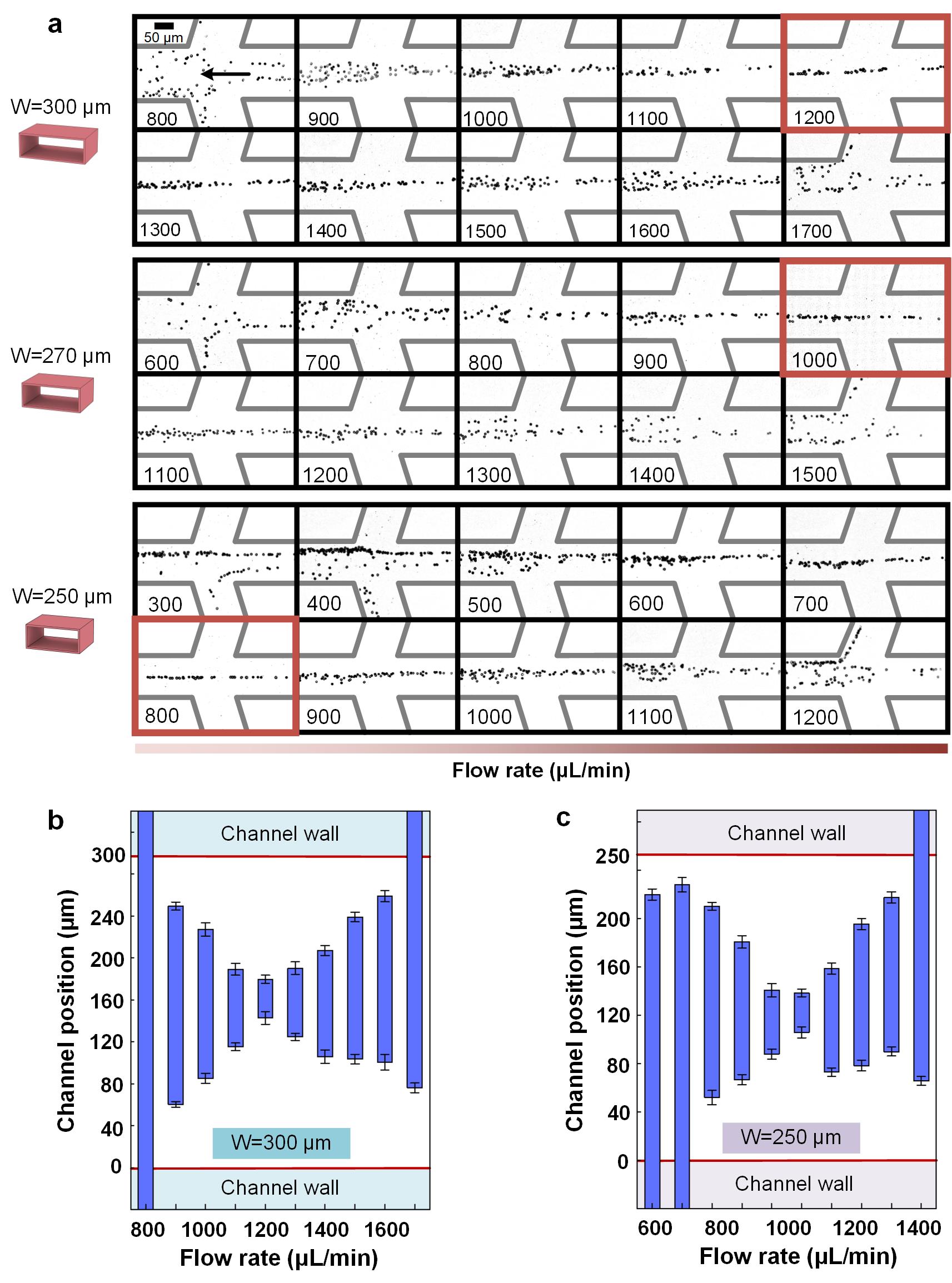


**Fig. S9:** **Characterization of the first stage serpentine channel. a,** Images illustrating the 15 μm particle distributions at different flow rate in the serpentine channels with widths of 250 μm, 270 μm, and 300 μm. **b,c,** Specific position distributions of the 15 μm particle in serpentine channels with widths of 300 μm (**b**) and 250 μm (**c**).


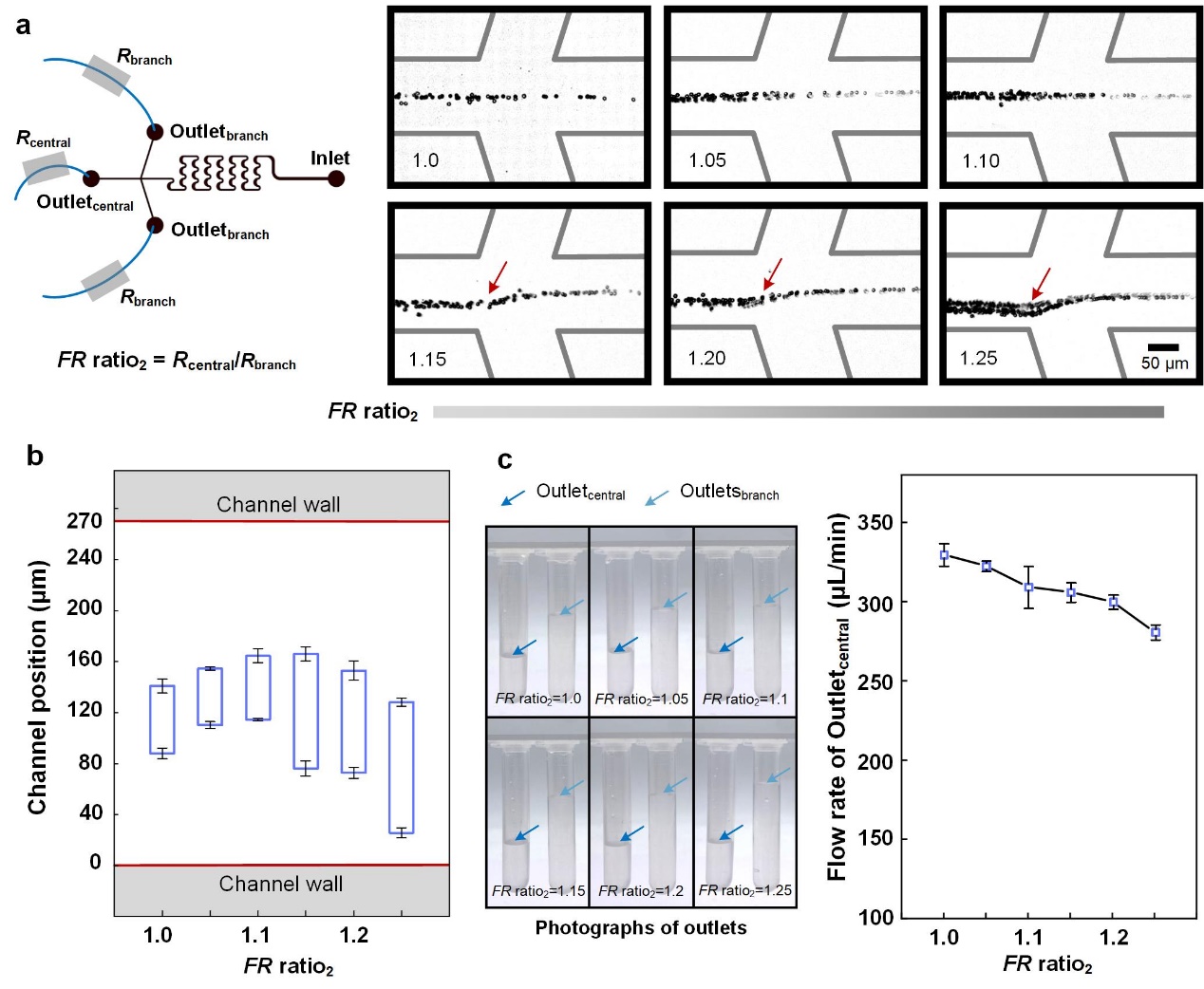


**Fig. S10: Characterization of the first stage serpentine channel. a,** Images illustrating the influence of flow resistance (*FR*) on particle focusing behavior. **b,** The influence of *FR* on the flow rate of the central outlet.


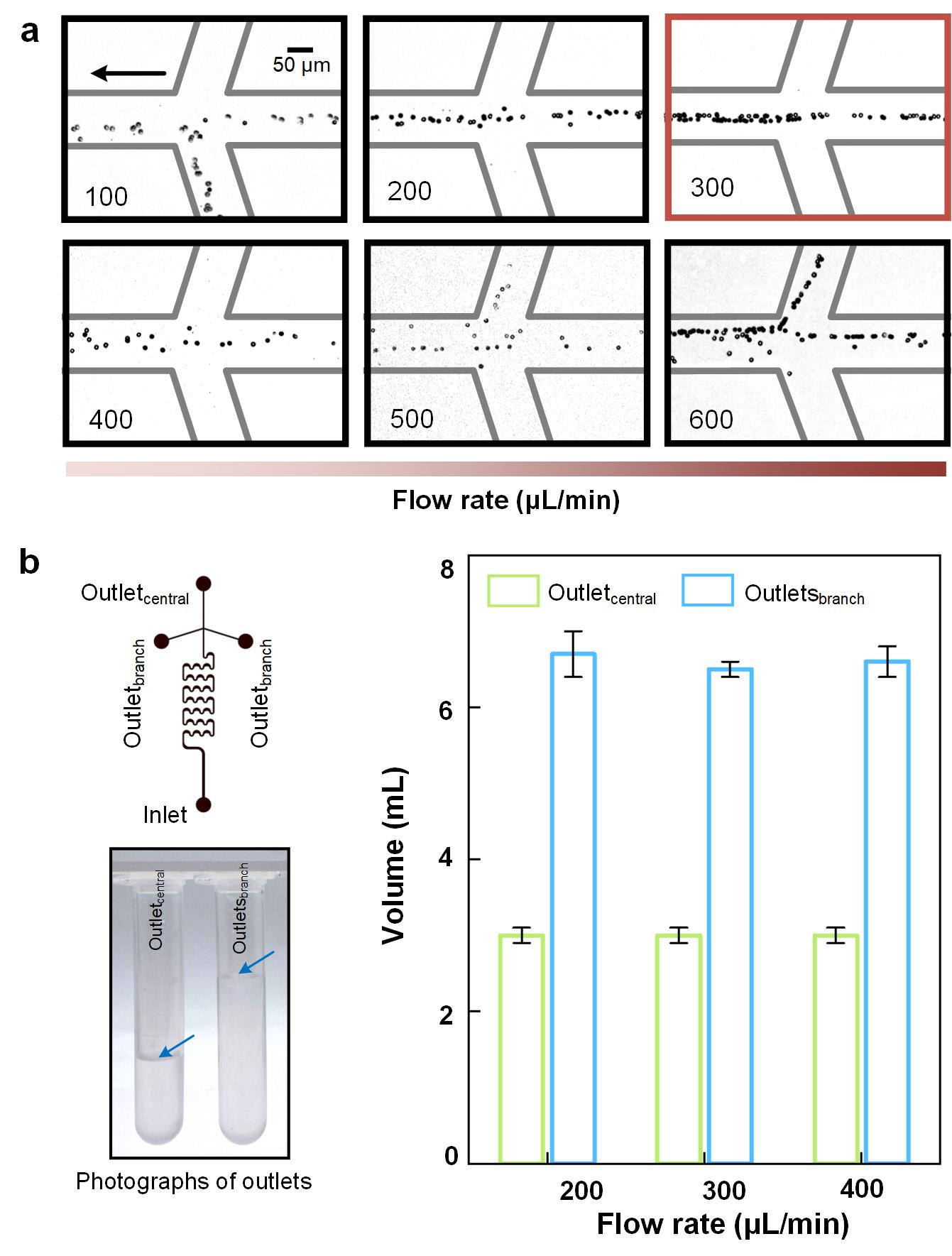


**Fig. S11: Characterization of the second stage serpentine channel. a,** Images illustrating the 15 μm particle distributions at different flow rate in the serpentine channels with width of 150 μm. **b,** The influence of flow rate on the liquid volume of central and branch outlets.


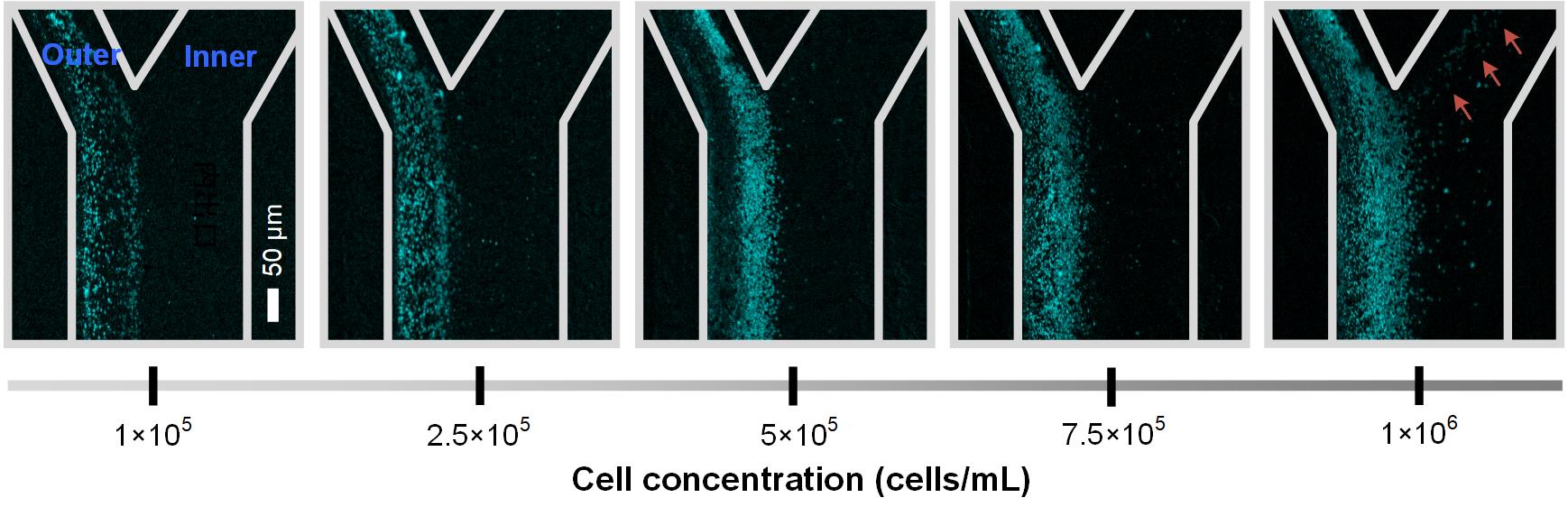


**Fig. S12: Characterization of the spiral channel with trapezoid cross-section.** Images illustrating the influence of WBC concentration on WBC separation performance.


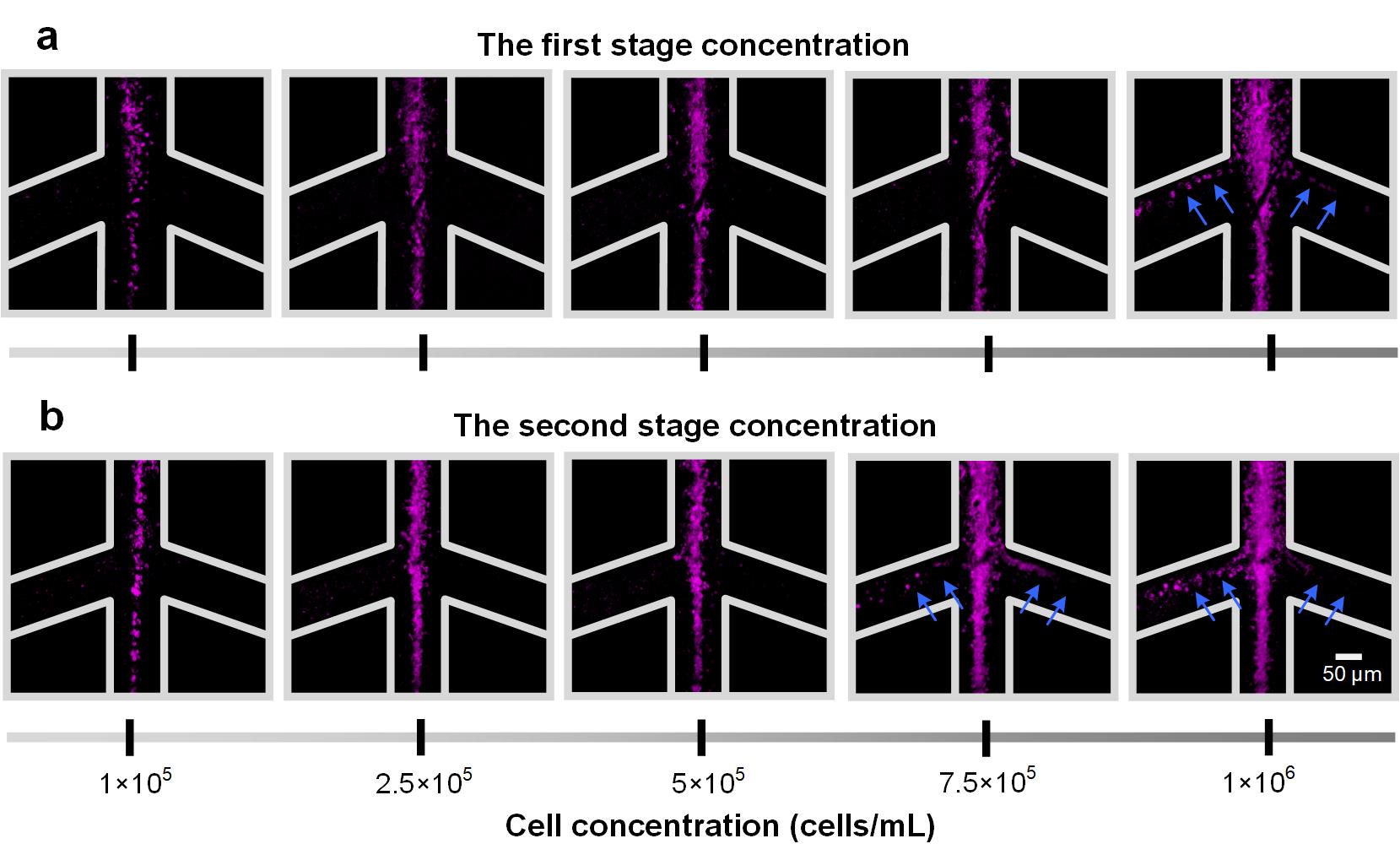


**Fig. S13: Characterization of the first and second stage serpentine channels. a,b,** Images illustrating the influence of tumor cell concentration on its concentration performance in first (**a**) and second (**b**) stage serpentine channels.


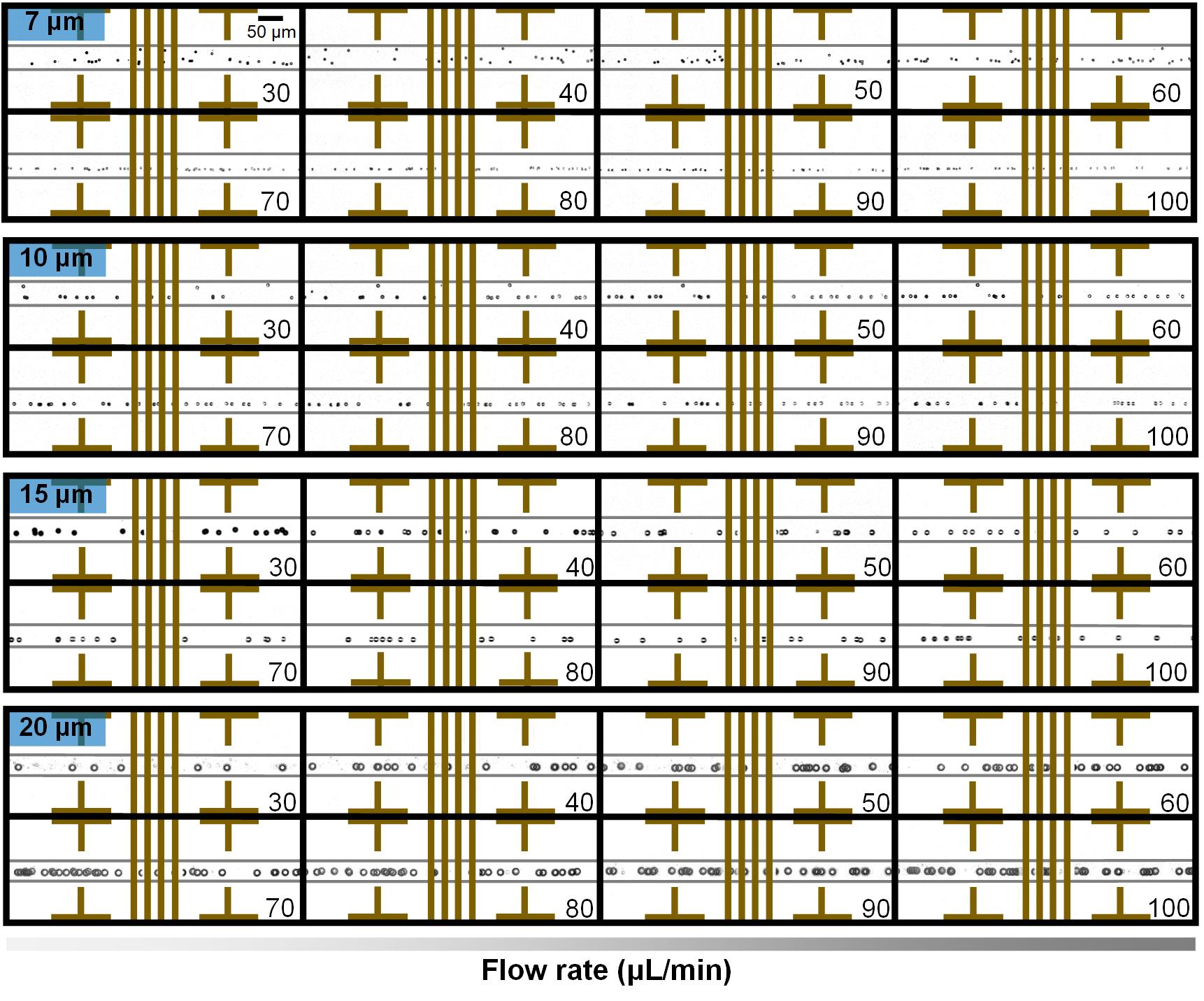


**Fig. S14: Characterization of the asymmetric serpentine channel in the detection module.** Images illustrating the distributions of 7 μm, 10 μm, 15 μm, 20 μm in the asymmetric serpentine channel at different flow rates.


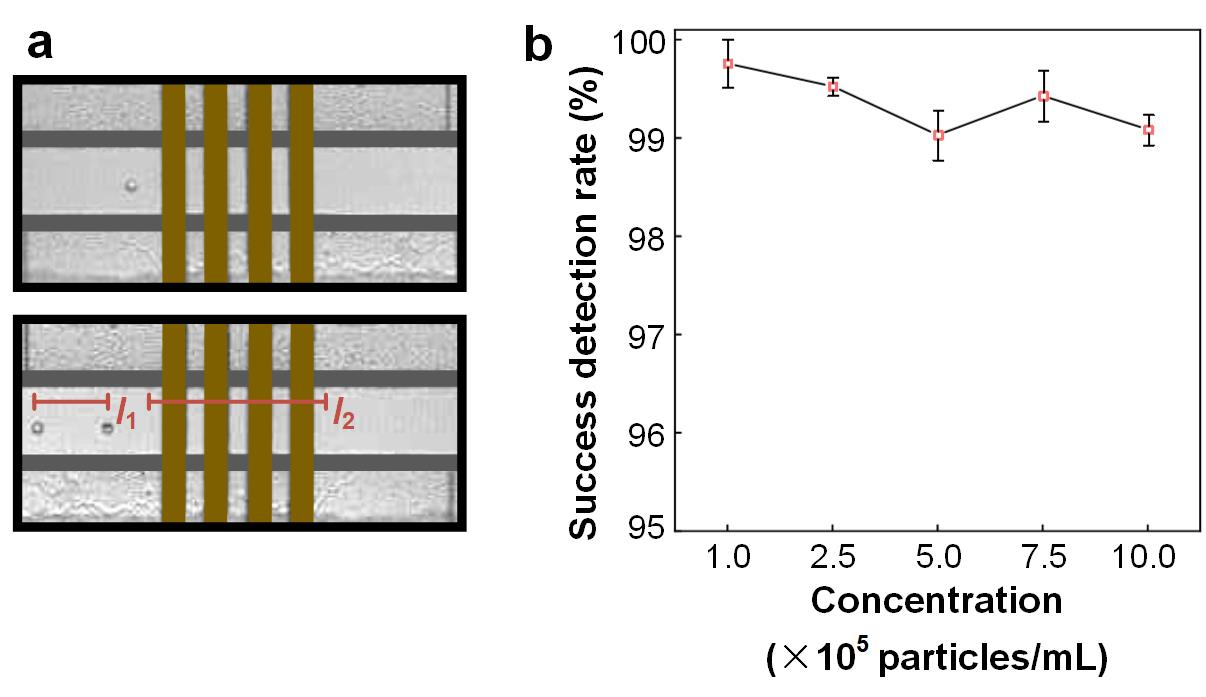


**Fig. S15: Characterization of the asymmetric serpentine channel in the detection module. a,** Images illustrating success or unsuccess detection of particle. As the *l*_1_ < *l*_2_, it can be considered as an unsuccess detection, where *l*_1_ is the distance between adjacent particles, *l*_2_ is the length of the detection region. **b,** Relationship between the particle concentration and particle success detection rate.


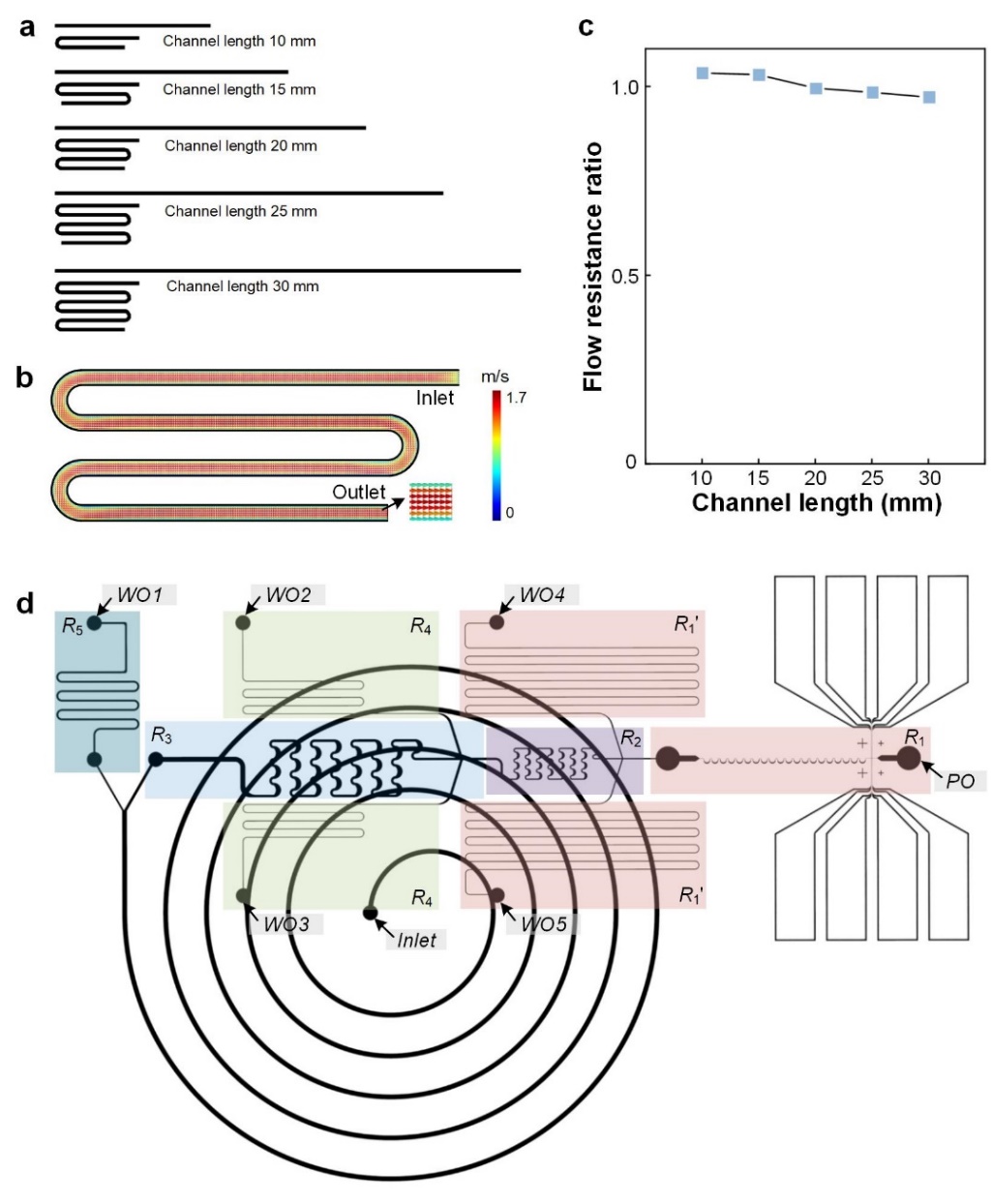


**Fig. S16:** **Design of the S-shaped channel for flow resistance matching. a,** Dimensions of the conventional straight channels and S-shaped channels in this work. **b,** Planar flow velocity distribution in the S-shaped channels. **c,** The flow resistance ratio between straight channels and S-shaped channels under the same channel length. It was found that the difference in flow resistance between these two types of channels with the same length is not significant. In process of the flow resistance matching, the required flow resistance was calculated according to straight channel owing to its ease calculation; a S-shaped channel with the same length as the straight channel was then designed and fabricated; the pressure-flow experiments were then carried out for correcting the flow resistance changes caused by changes in channel shape.^5^ Compared to the straight channel, the S-shaped channel is more conducive to subsequent module integration. **d,** Schematics of the eScan-chip after module integration and flow resistance matching. The flow resistance $R_{1}'$, $R_{4}$, and $R_{5}$ are calculate according to the $R_{1}$, $R_{2}$, and $R_{3}$, where $R_{1}'=R_{1}/1.1$, $R_{4}=\frac{10}{11}R_{2}+\frac{25}{88}R_{1}$, and $R_{5}=\frac{1}{2}R_{3}+\frac{5}{32}R_{2}+\frac{25}{512}R_{1}$.


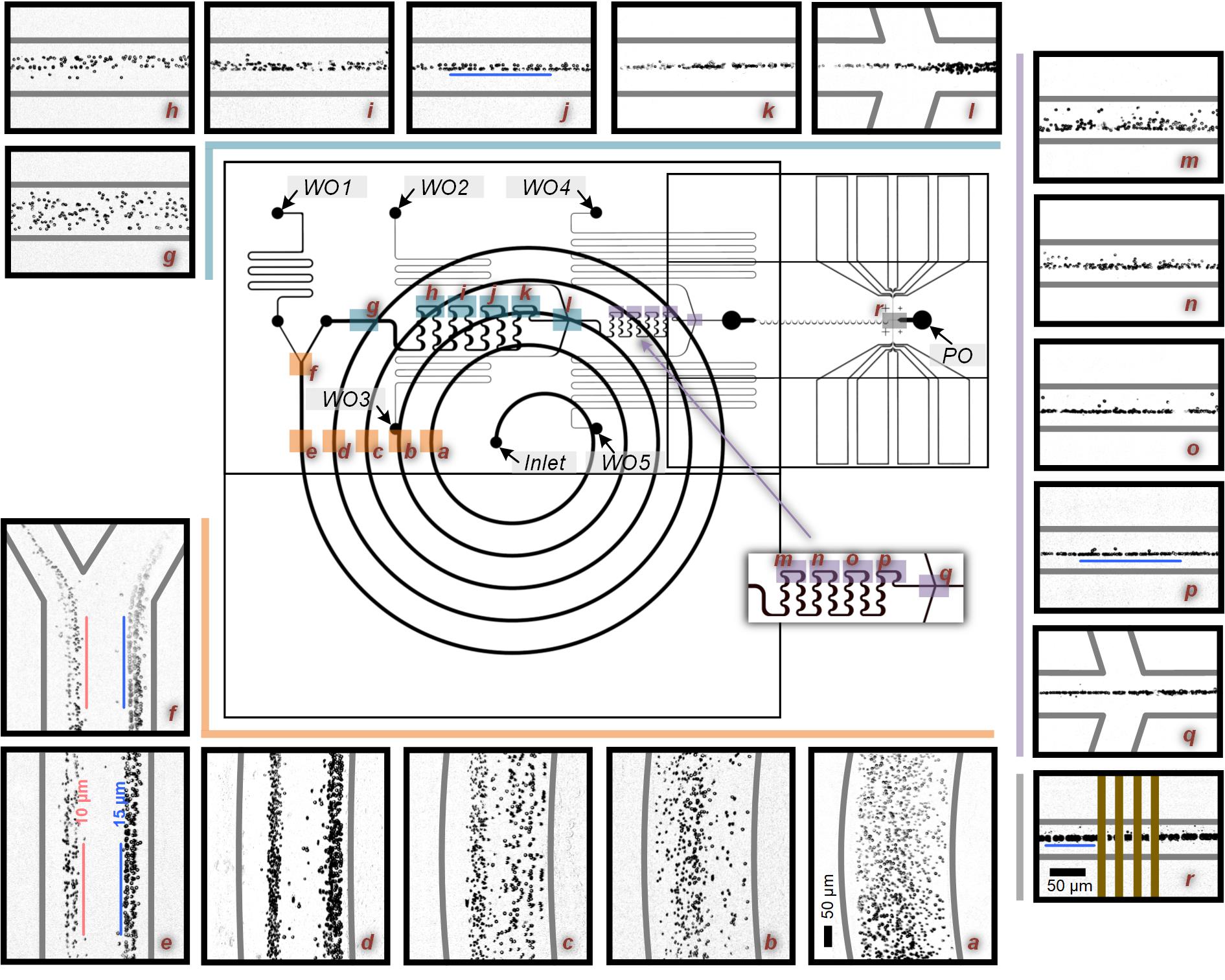


**Fig. S17: Characterization of the eScan-chip.** Particle distributions in the separation module (*a*–*f*), concentration module (*g*–*q*), and detection module (*r*) when the mixed particle sample pump into the chip through the inlet.


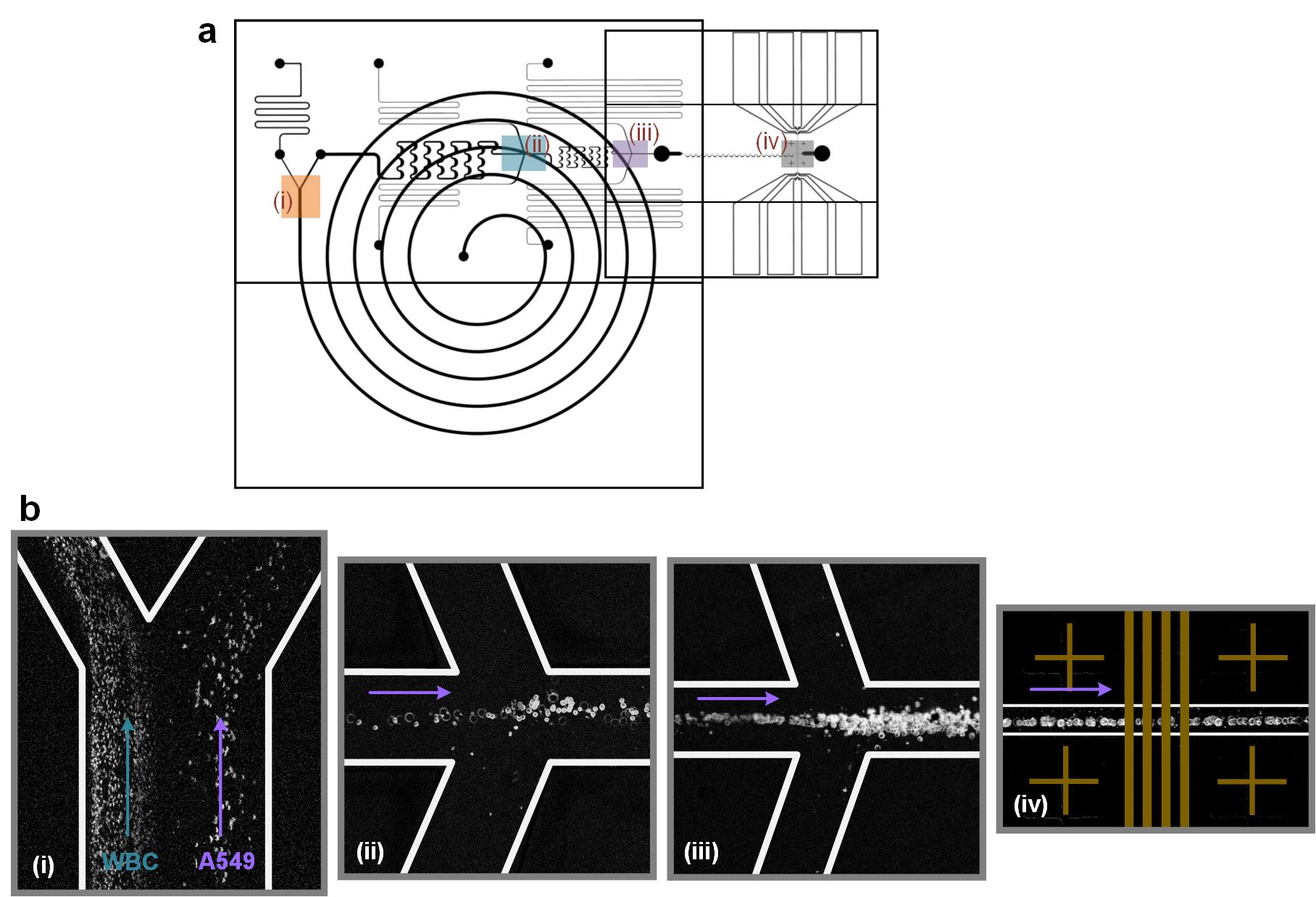


**Fig. S18:** **Characterization of the eScan-chip. a,** Schematics of the eScan-chip. Positions marked in the schematics locate at the end of the spiral (i), first stage serpentine (ii), second stage serpentine (iii), and asymmetric serpentine channel (iv), respectively. **b,** Images illustrating the cell distributions (WBC and A549) at the marked positions.


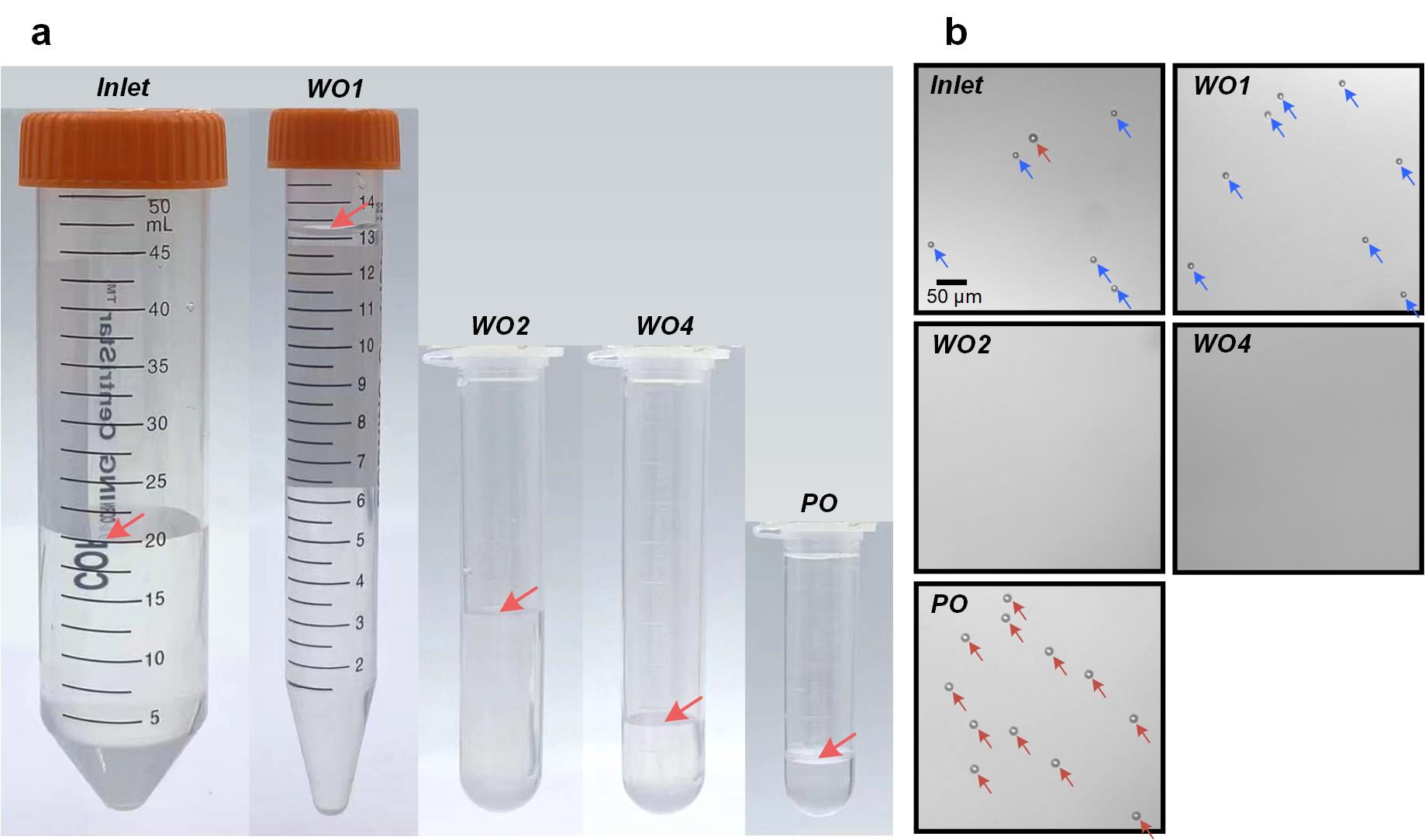


**Fig. S19:** **Characterization of the eScan-chip. a,** Photographs of the liquid collected from the *inlet*, waste outlet 1 (*WO1*), *WO2*, *WO4*, and product outlet (*PO*). **b,** Microscopic images of the liquid collected from the *inlet*, *WO1*, *WO2*, *WO4*, and *PO*. As the *WO2* and *WO3* are symmetrical, the *WO4* and *WO5* are symmetrical, relevant photographs and images are omitted to avoid repetition.


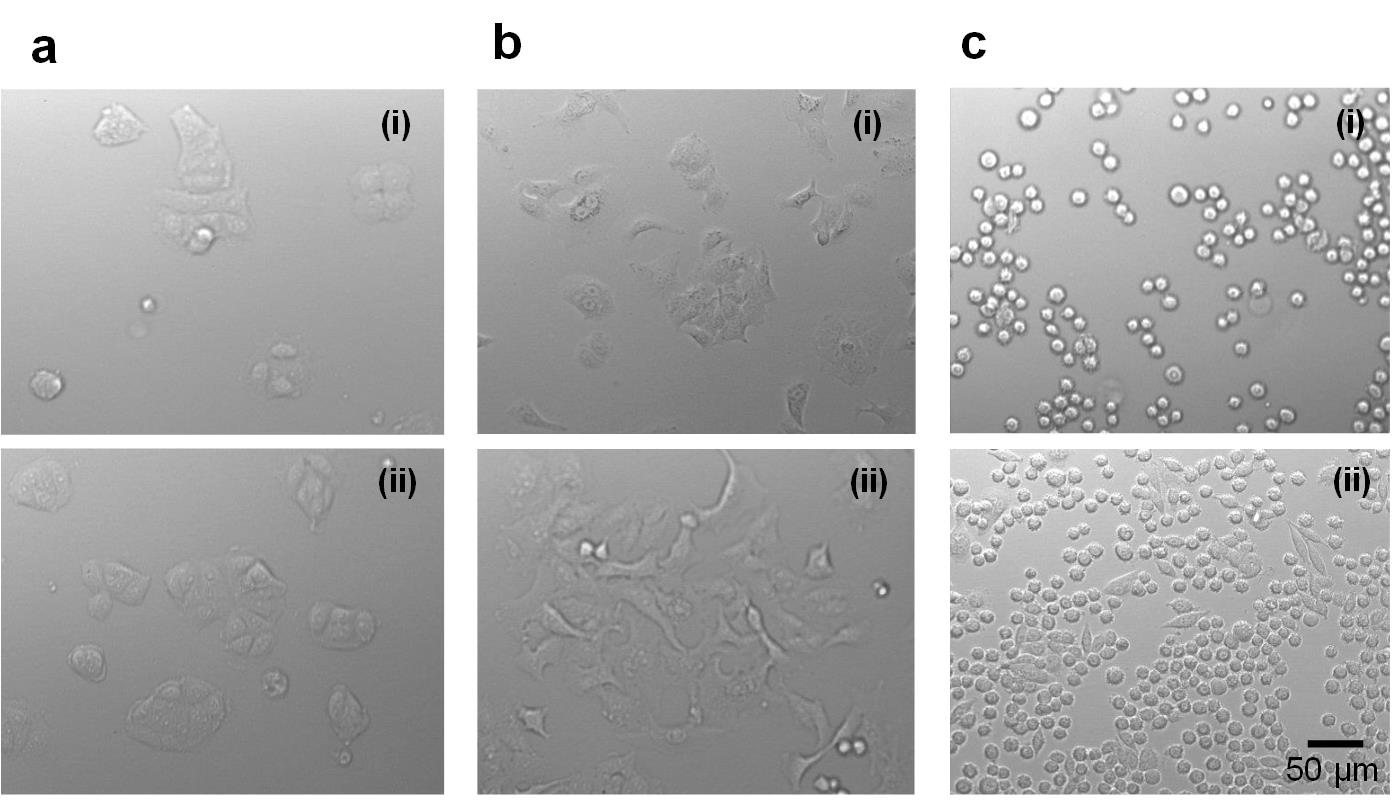


**Fig. S20: Characterization of the eScan-chip. a,** A549 cells. **b,** MCF-7 cells. **c,** SW480 cells. The tumor cells collected from the eScan-chip were re-cultured for 24 h (i), and 48 h (ii).


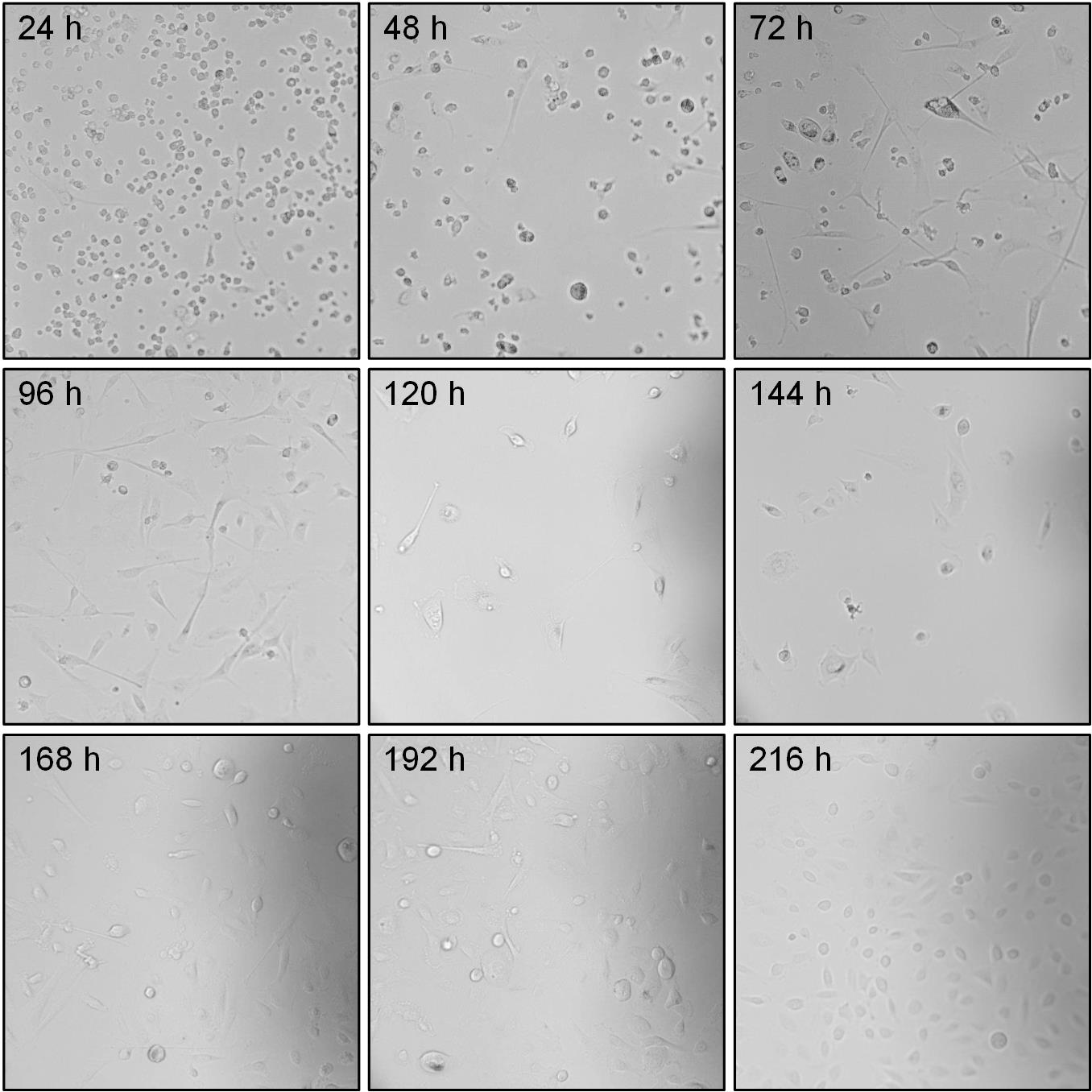


**Fig. S21: Characterization of the eScan-chip.** Re-culture of tumor cells (24 h–216 h) in malignant pleural effusion after being processed using the eScan-chip.


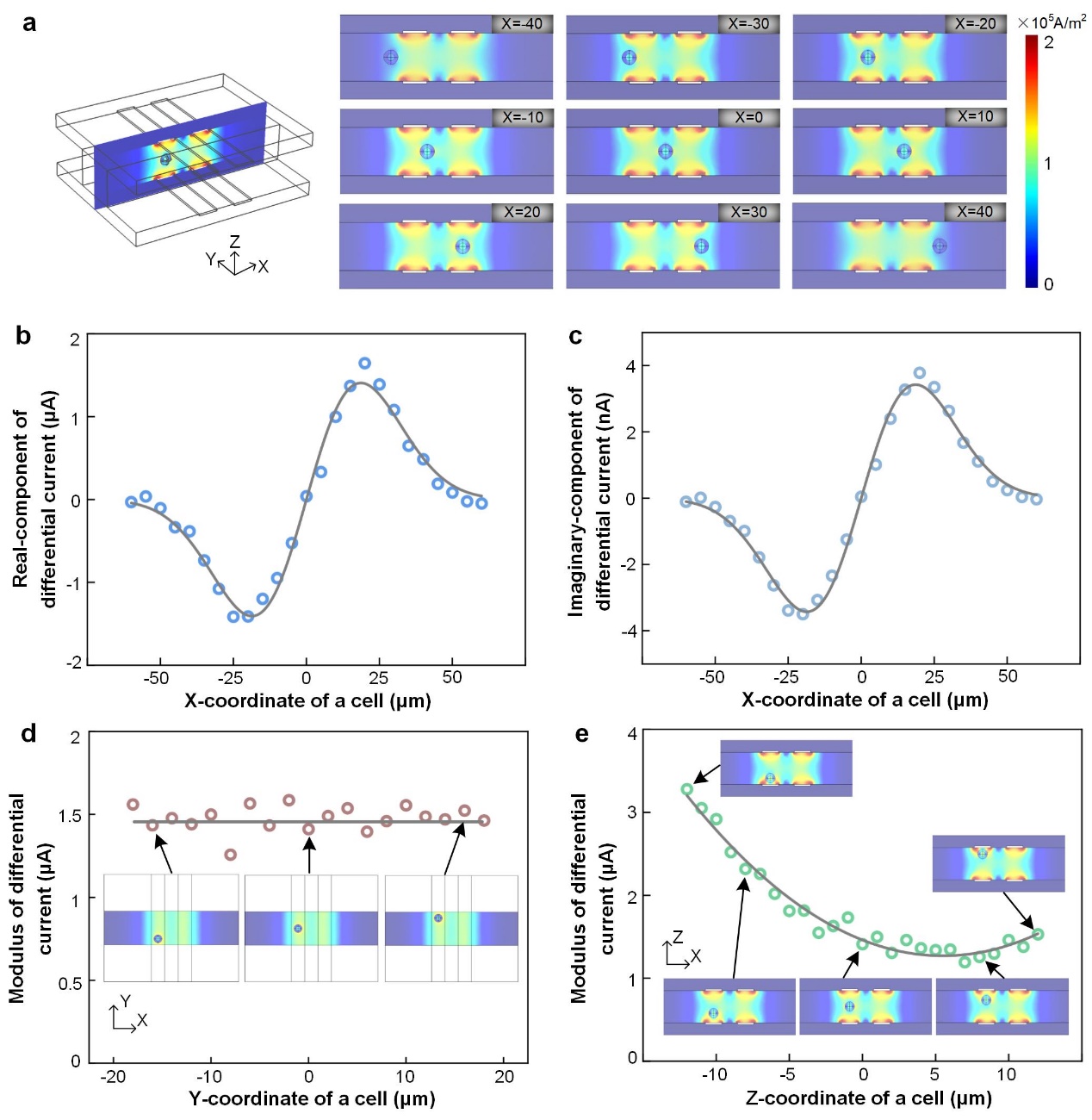


**Fig. S22: Numerical simulation of the cell sensing in impedance cytometry. a,** Changes of current density when cell passing through the detection region with two pairs of face-to-face electrodes. Two top electrodes are applied AC electric field and served as excitation electrodes, and two bottom electrodes are served as sensing electrodes. **b,c,** Real (**b**) and imaginary (**c**) component response of the differential current, where the differential current is obtained through connecting two sensing electrodes in series. Obviously, these responses all exhibit Gaussian curve shape with two central symmetric peaks. **d,** Modulus response of the differential current when cell moving from Y = -20 μm to Y = 20 μm at the XY plane. According to the fitting line of discrete points, the positional deviation of cells in the XY plane will not have a significant impact on detection signal. **e,** Modulus response of the differential current when cell moving from Z = -12 μm to Z = 12 μm at the XZ plane. The fitting cure of discrete points appears the shape of a quadratic parabola, which indicates that the detection signal increase when the cell passing through the detection region with a trajectory closer to the sensing electrode.^6, 7^


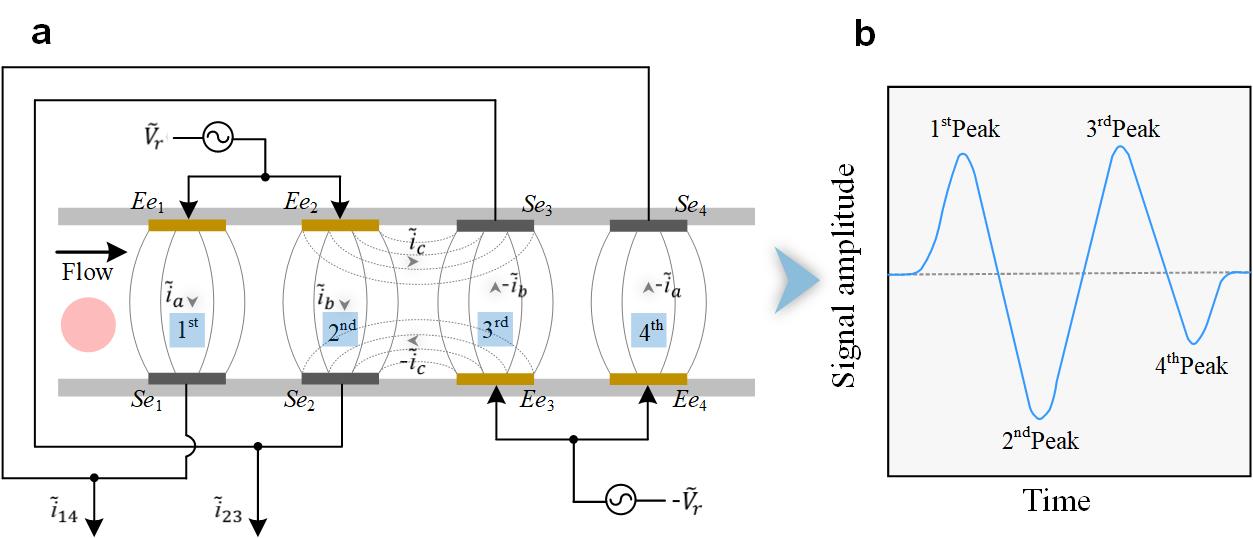


**Fig. S23: Theoretical analysis of the cell sensing in impedance cytometry. a,** Current distribution of the detection region with four pairs of face-to-face electrodes. Two signals with the same voltage but phase shifted by 180° ($\tilde{V}_{r}$ and ${-\tilde{V}}_{r}$) are applied to the corresponding exciting electrodes (*Ee*_1_, *Ee*_2_, *Ee*_3_, *Ee*_4_); the detection signals are sensed through four sensing electrodes (*Se*_1_, *Se*_2_, *Se*_3_, *Se*_4_). From the electrode configuration, the sensing current acquired on *Se*_1_ mainly composed of ${\tilde{\text{i}}}_{\text{a}}$, while the sensing current acquired on *Se*_1_ mainly composed of ${\tilde{\text{i}}}_{\text{b}}$ and $-{\tilde{\text{i}}}_{\text{c}}$. As the central symmetric of the detection region, the sensing current acquired on *Se*_3_ mainly composed of $-{\tilde{\text{i}}}_{\text{b}}$ and ${\tilde{\text{i}}}_{\text{c}}$, while the sensing current acquired on *Se*_4_ mainly composed of $-{\tilde{\text{i}}}_{\text{a}}$. **b,** Typical signal when cell passing through the lower equilibrium position of the detection region (marked 1^st^, 2^nd^, 3^rd^, 4^th^ in **a**). The sensing signal experiencing twice differential exhibit Gaussian curve shape with four alternating peaks. For the first current differential, the *Se*_1_ and *Se*_4_ are connected in series and generate ${\tilde{\text{i}}}_{\text{14}}$, and the *Se*_2_ and *Se*_3_ are connected in series and generate ${\tilde{\text{i}}}_{\text{23}}$. Then, the ${\tilde{\text{i}}}_{\text{14}}$ and ${\tilde{\text{i}}}_{\text{23}}$ are differentiated for the second current differential.

When no cell passing through the detection region, the total differential current signal of the system $\Delta{\tilde{\text{i}}}_{diff}^{0}$ can be calculated as:

$\Delta{\tilde{\text{i}}}_{diff}^{0}={\tilde{\text{i}}}_{\text{23}}\text{-}{\tilde{\text{i}}}_{\text{14}}=[({\tilde{\text{i}}}_{\text{b}}-{\tilde{\text{i}}}_{\text{c}})+({\tilde{\text{i}}}_{\text{c}}-{\tilde{\text{i}}}_{\text{b}})]-[{\tilde{\text{i}}}_{\text{a}}+(-{\tilde{\text{i}}}_{\text{a}})]=0$ (1-1)

When the cell passing through the electrode region, various degrees of attenuation of sensing currents are induced, causing the $\Delta{\tilde{\text{i}}}_{diff}\neq0$, and generating sense signals related to cellular dielectric properties. For the ${\tilde{\text{i}}}_{\text{a}}$ and ${\tilde{\text{i}}}_{\text{b}}$, assuming that the current decreases by *k* and *l* times when the cell passes through the upper and lower equilibrium trajectories, respectively. According to the numerical simulation results in **Fig. 22**, it can be concluded that *k* < *l* when the cell passing through the lower equilibrium trajectory, where *k* and *l* ∈ (0,1). For the $-{\tilde{\text{i}}}_{\text{c}}$, assuming that the current decreases by *m* and *n* times when the cell passes through the 2^nd^ and 3^rd^ positions in the lower equilibrium trajectory, where *m* > *n*, *m* and *m* ∈ (0,1). As the central symmetric of the detection region, the attenuation factors of sensing currents ($-{\tilde{\text{i}}}_{\text{a}}, -{\tilde{\text{i}}}_{\text{b}}, \mathrm{and} {\tilde{\text{i}}}_{\text{c}}$) are same with the former when cell passing through the upper equilibrium trajectory.

Therefore, when the cell moving to the 1^st^ position in the lower equilibrium trajectory, the $\Delta{\tilde{\text{i}}}_{diff}^{1}$ can be calculated as:

$\Delta{\tilde{\text{i}}}_{diff}^{1}=l{\tilde{\text{i}}}_{\text{a}}$ (1-2)

When the cell moving to the 2^nd^ position in the lower equilibrium trajectory, the $\Delta{\tilde{\text{i}}}_{diff}^{2}$ can be calculated as:

$\Delta{\tilde{\text{i}}}_{diff}^{2}=-l{\tilde{\text{i}}}_{\text{b}}+m{\tilde{\text{i}}}_{\text{c}}$ (1-3)

As the face-to-face arrangement of *Ee*_2_ and *Se*_2_, and the coplanar arrangement of *Se*_2_ and *Ee*_3_, it can be inferred that ${\tilde{\text{i}}}_{\text{b}}>{\tilde{\text{i}}}_{\text{c}}$. Moreover, as the cell passing through the middle of *Ee*_2_ and *Se*_2_, and passing through the upper side of *Se*_2_ and *Ee*_3_, the former causes greater current attenuation than the latter, indicating that *l* > *m*. Therefore, it can be calculated that $\Delta{\tilde{\text{i}}}_{diff}^{2}<0$.

Similarly, when the cell moving to the 3^rd^ position in the lower equilibrium trajectory, the $\Delta{\tilde{\text{i}}}_{diff}^{3}$ can be calculated as:

$\Delta{\tilde{\text{i}}}_{diff}^{3}=k{\tilde{\text{i}}}_{\text{b}}+n{\tilde{\text{i}}}_{\text{c}}$ (1-4)

When the cell moving to the 4^th^ position in the lower equilibrium trajectory, the $\Delta{\tilde{\text{i}}}_{diff}^{4}$ can be calculated as:

$\Delta{\tilde{\text{i}}}_{diff}^{4}=-k{\tilde{\text{i}}}_{\text{a}}$ (1-5)

According to the result that $\Delta{\tilde{\text{i}}}_{diff}^{1}$, $\Delta{\tilde{\text{i}}}_{diff}^{2}$, $\Delta{\tilde{\text{i}}}_{diff}^{3}$, and $\Delta{\tilde{\text{i}}}_{diff}^{4}$ are alternating positive and negative, indicating that when the cell passes through the lower equilibrium trajectory, its signal exhibit Gaussian curve shape with four alternating peaks, and the peaks generated at 1^st^, 2^nd^, 3^rd^, and 4^th^ positions marked in **a** correspond to 1^st^ Peak, 2^nd^ Peak, 3^rd^ Peak, and 4^th^ Peak marked in **b**. As the central symmetric of the detection region, when the same cell passes through the upper equilibrium trajectory, its signal is equal in size and centrally symmetrical to the signal generated in the lower equilibrium trajectory.

As a result, it can be concluded that all cells passing the detection region through the upper or lower equilibrium trajectory can be detected accurately. Additionally, for the convenience of subsequent statistical calculations, the position marking of the central symmetric signal peak is also central symmetric. For example, if a cell passes through the upper equilibrium trajectory of the diagram, its signal peak at *Ee*_1_ position is marked as 4^th^Peak.


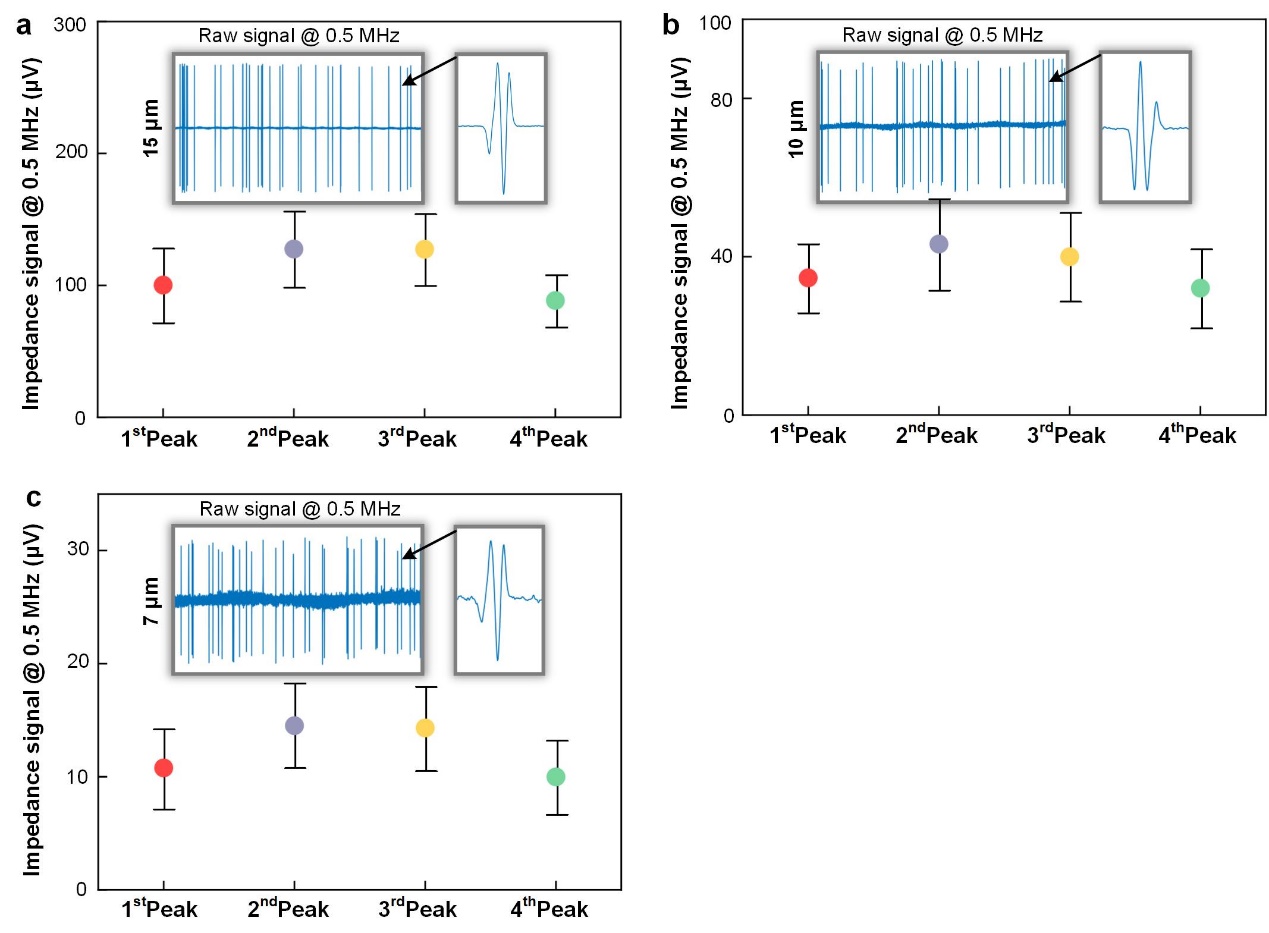


**Fig. S24: Statistics of particle raw signals. a-c,** Signal peak amplitude statistics of 15 μm (**a**), 10 μm (**b**), 7 μm (**c**) particles at 0.5 MHz frequency. The insets exhibit the raw signals of particles undergoing impedance detection.


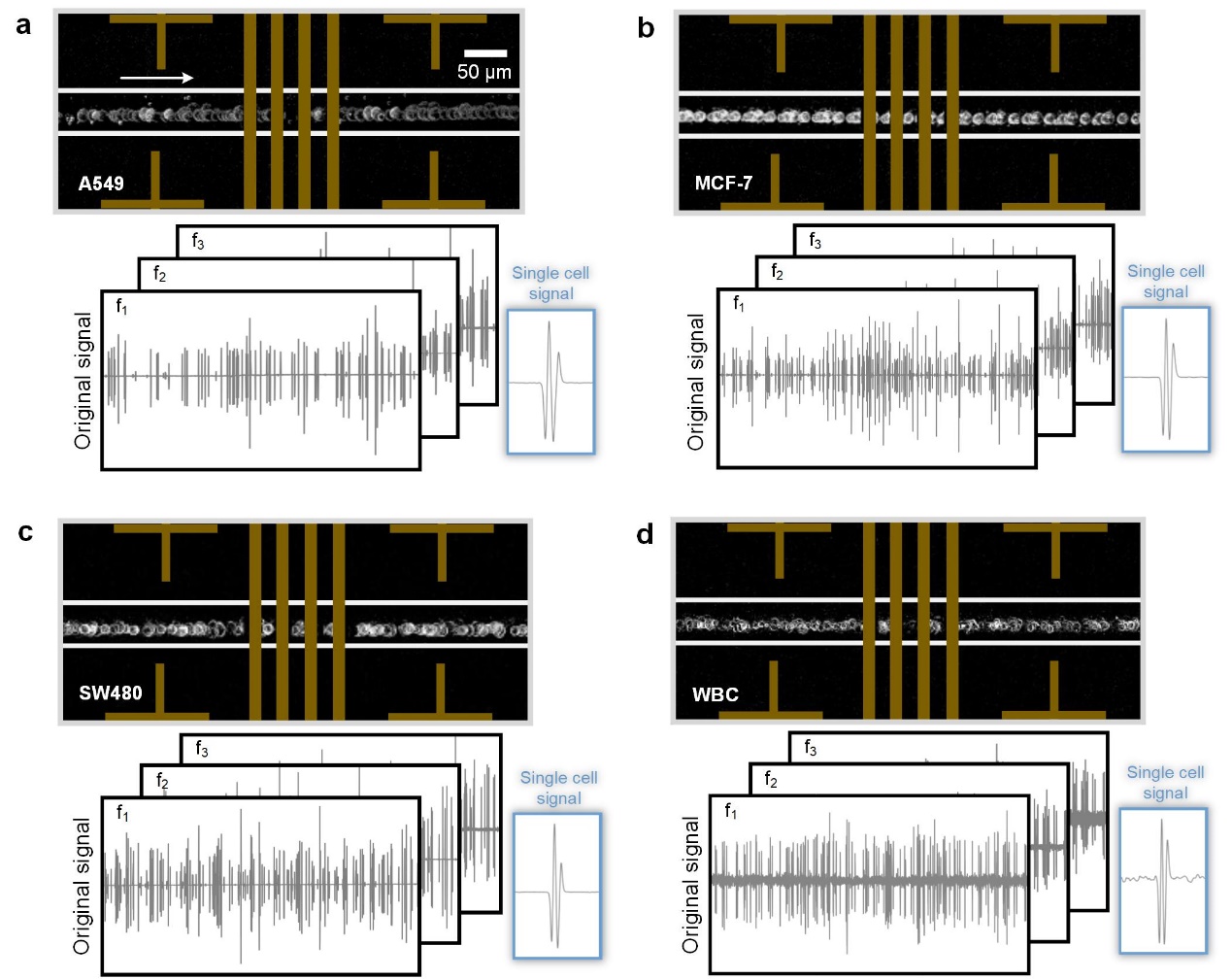


**Fig. S25: Illustrations of cell focusing behaviors and corresponding raw signals. a-d,** Raw signals and focusing behaviors of A549 (**a**), MCF-7 (**b**), SW480 (**c**), and WBC (**d**).


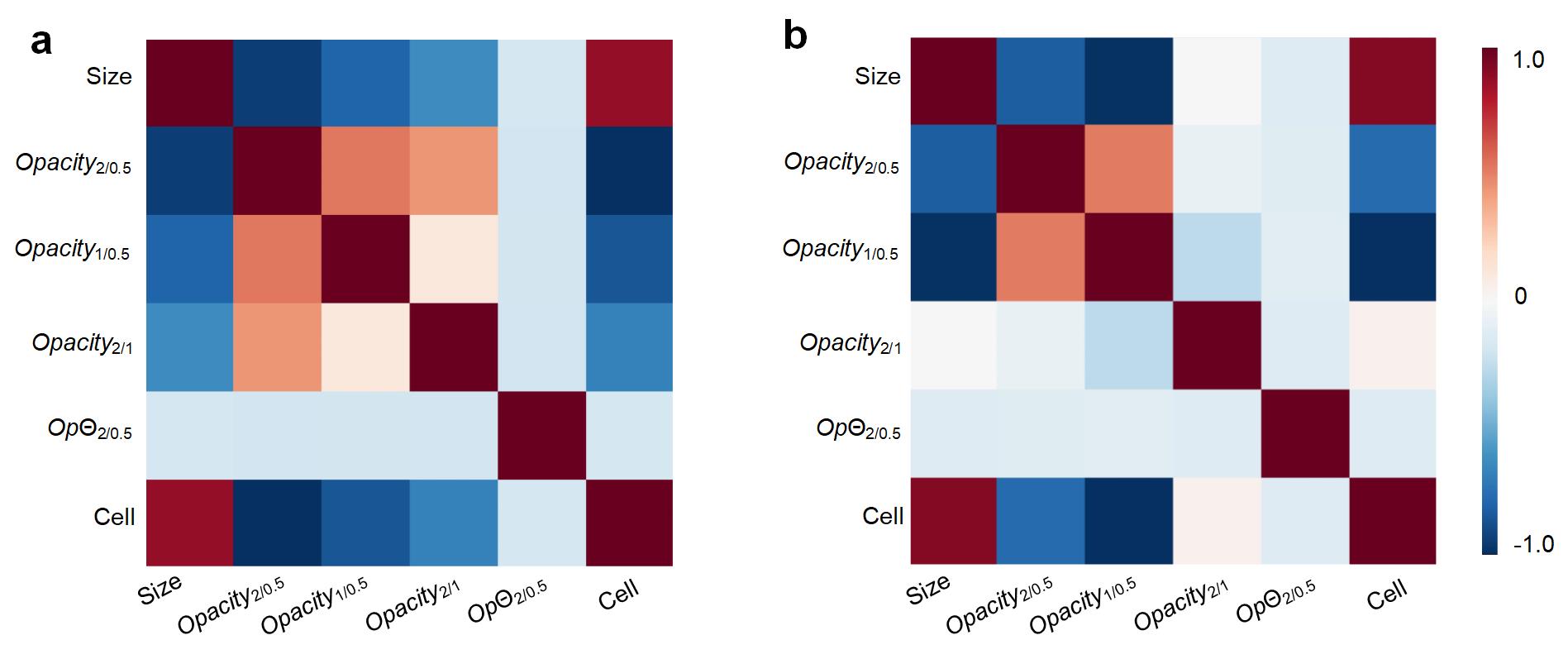


**Fig. S26: Correlation matrix of multiple electrical parameters. a,** A549 and WBC. **b,** SW480 and WBC.


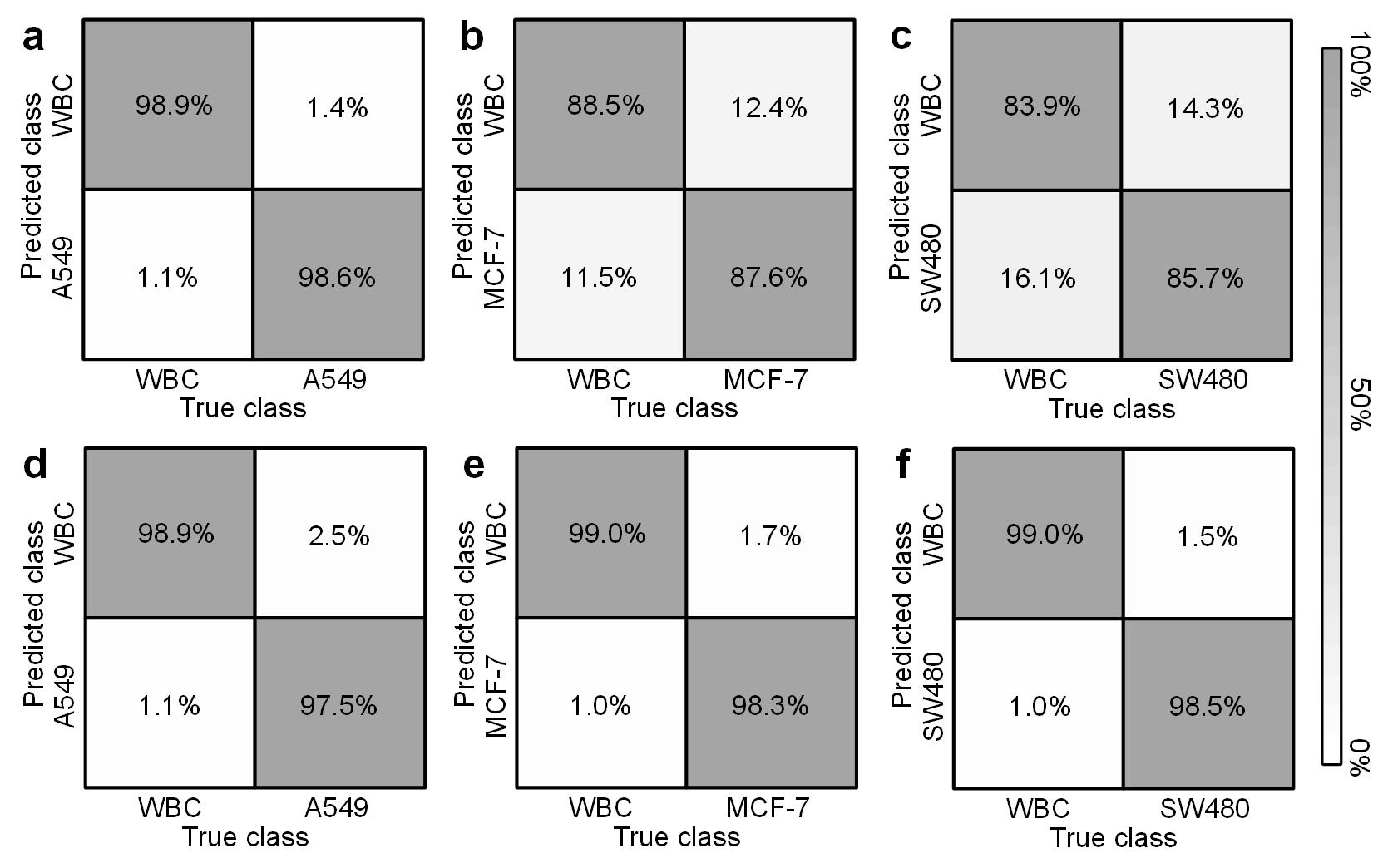


**Fig. S27: Confusion matrix of machine learning results using multiple parameters. a–c,** Classification of A549&WBC (**a**), MCF-7&WBC (**b**), SW480&WBC (**c**) using k-nearest neighbor (KNN) algorithm. **d–f,** Classification of A549&WBC (**d**), MCF-7&WBC (**e**), SW480&WBC (**f**) using logistic regression (LR) algorithm.


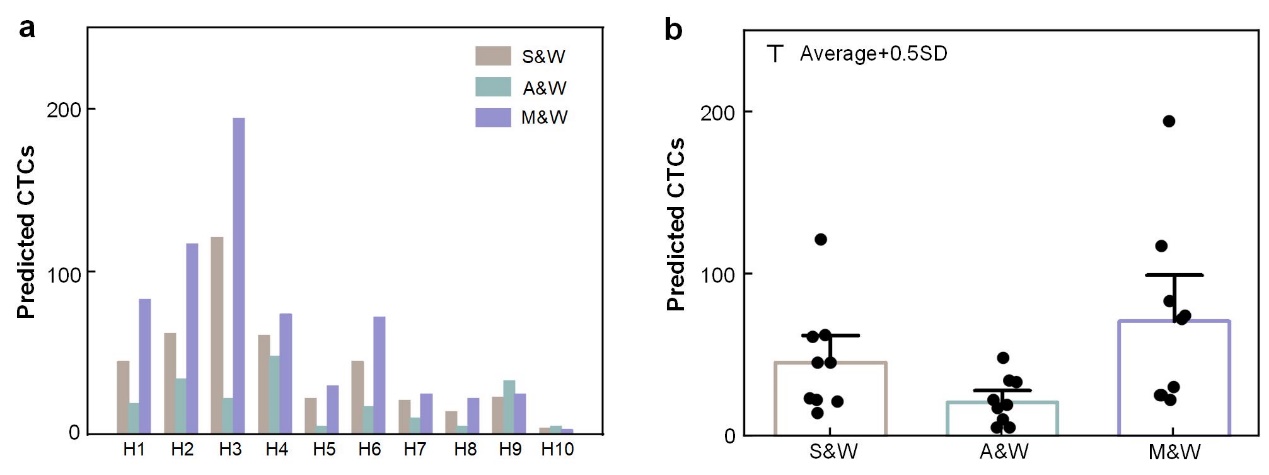


**Fig. S28: Performance of the pretrained CytoNet. a,** Predicted CTCs of each health donors using CytoNet pretrained by SW480&WBC (S&W), A549&WBC (A&W), and MCF-7&WBC (M&W) datasets. **b,** Comparison of predicted CTCs of health donors using CytoNet pretrained by these three types of datasets.


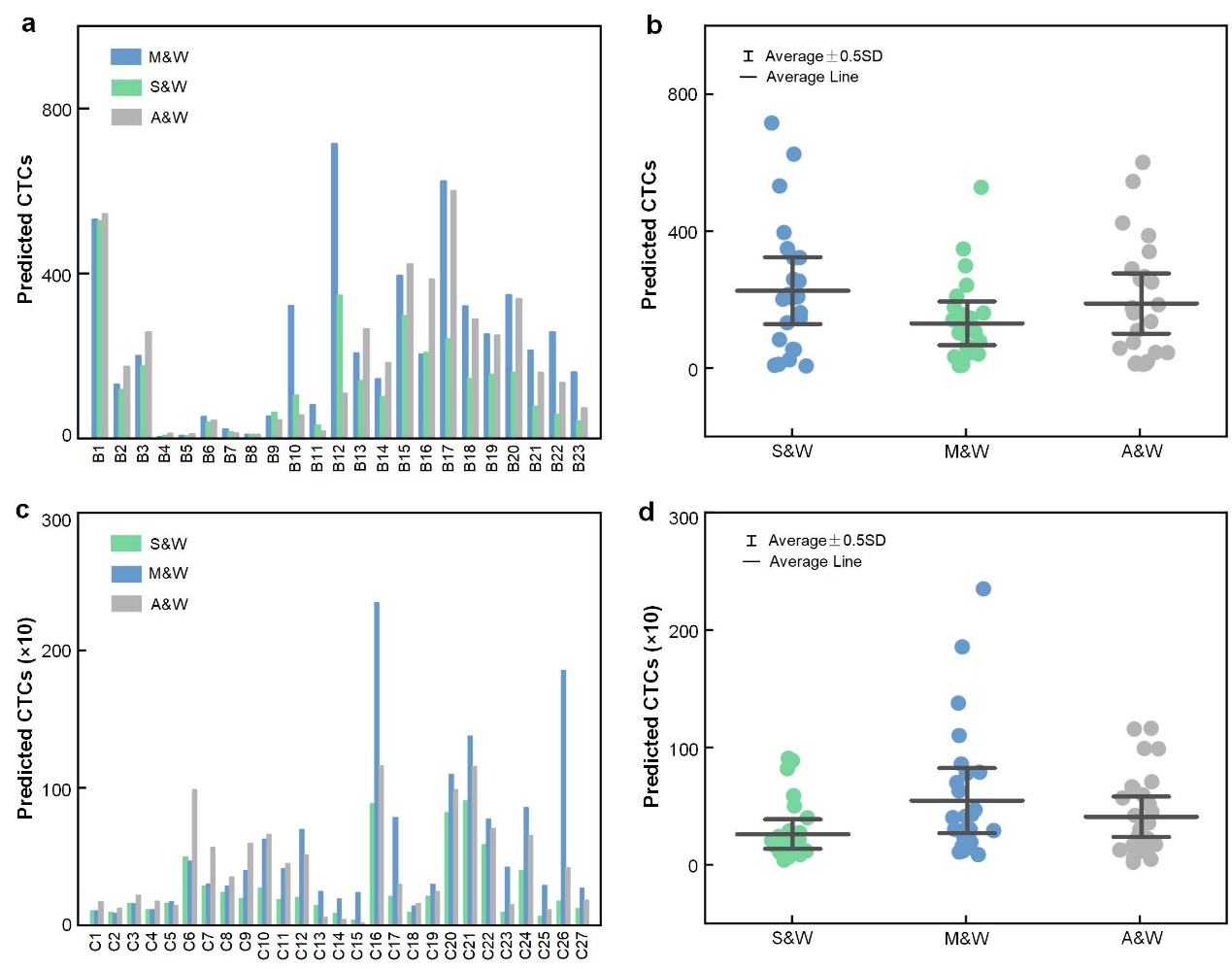


**Fig. S29: Performance of the pretrained CytoNet. a,** Predicted CTCs of each breast cancer patient using CytoNet pretrained by M&W, S&W, and A&W datasets. **b,** Comparison of predicted CTCs of breast cancer patient using CytoNet pretrained by these three types of datasets. **c,** Predicted CTCs of each colorectal cancer patient using CytoNet pretrained by S&W, M&W, and A&W datasets. **d,** Comparison of predicted CTCs of colorectal cancer patient using CytoNet pretrained by these three types of datasets.


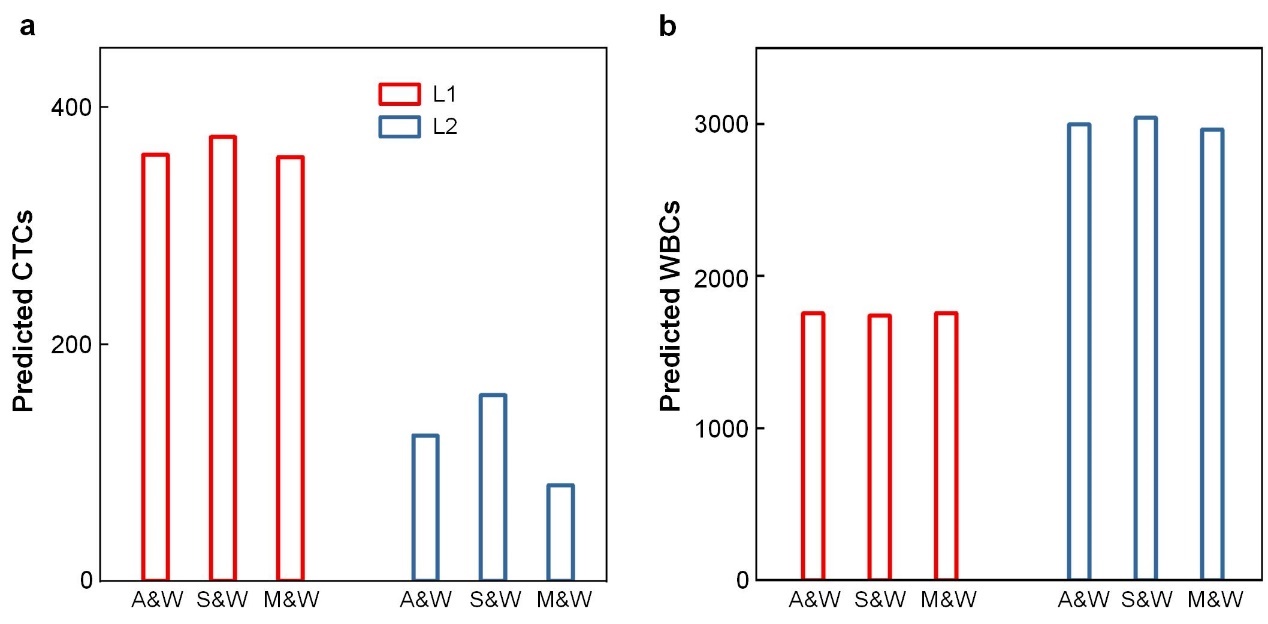


**Fig. S30: Performance of the pretrained CytoNet. a,** Predicted CTCs of each lung cancer patient using CytoNet pretrained by M&W, S&W, and A&W datasets. **b,** Predicted WBCs of each lung cancer patient using CytoNet pretrained by M&W, S&W, and A&W datasets.


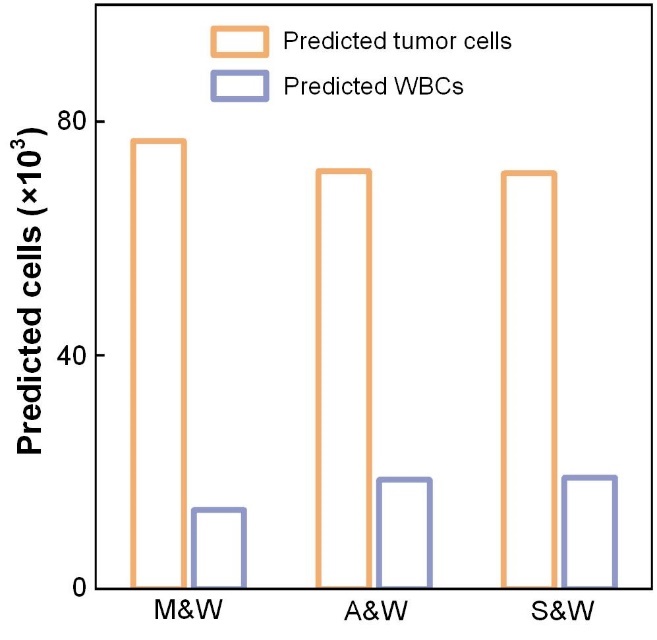


**Fig. S31: Performance of the pretrained CytoNet.** Predicted WBCs and tumor cells from 50 mL malignant pleural effusion of breast cancer patient using CytoNet pretrained by M&W, S&W, and A&W datasets.

**Table S1: Clinical and pathological data of patients.**

| No. | Age/Sex | Disease | Stage | Sample |
| --- | --- | --- | --- | --- |
| B1 | 35/W | Breast cancer | I | Peripheral blood |
| B2 | 39/W | Breast cancer | II | Peripheral blood |
| B3 | 57/W | Breast cancer | III | Peripheral blood |
| B4 | 42/W | Breast cancer | II | Peripheral blood |
| B5 | 74/W | Breast cancer | I | Peripheral blood |
| B6 | 69/W | Breast cancer | I | Peripheral blood |
| B7 | 47/W | Breast cancer | I | Peripheral blood |
| B8 | 42/W | Breast cancer | N/A | Peripheral blood |
| B9 | 36/W | Breast cancer | I | Peripheral blood |
| B10 | 55/W | Breast cancer | II | Peripheral blood |
| B11 | 68/W | Breast cancer | I | Peripheral blood |
| B12 | 56/W | Breast cancer | IV | Peripheral blood |
| B13 | 59/W | Breast cancer | I | Peripheral blood |
| B14 | 38/W | Breast cancer | N/A | Peripheral blood |
| B15 | 57/W | Breast cancer | N/A | Peripheral blood |
| B16 | 33/W | Breast cancer | I | Peripheral blood |
| B17 | 33/W | Breast cancer | N/A | Peripheral blood |
| B18 | 60/W | Breast cancer | N/A | Peripheral blood |
| B19 | 48/W | Breast cancer | N/A | Peripheral blood |
| B20 | 49/W | Breast cancer | I | Peripheral blood |
| B21 | 57/W | Breast cancer | III | Peripheral blood |
| B22 | 57/W | Breast cancer | N/A | Peripheral blood |
| B23 | 56/W | Breast cancer | N/A | Peripheral blood |
| B24 | 56/W | Breast cancer | IV | Pleural effusion |
| C1 | 75/M | Colorectal cancer | I | Peripheral blood |
| C2 | 66/M | Colorectal cancer | III | Peripheral blood |
| C3 | 77/M | Colorectal cancer | I | Peripheral blood |
| C4 | 55/M | Colorectal cancer | I | Peripheral blood |
| C5 | 48/W | Colorectal cancer | III | Peripheral blood |
| C6 | 74/M | Colorectal cancer | II | Peripheral blood |
| C7 | 59/M | Colorectal cancer | II | Peripheral blood |
| C8 | 59/M | Colorectal cancer | II | Peripheral blood |
| C9 | 68/W | Colorectal cancer | II | Peripheral blood |
| C10 | 78/M | Colorectal cancer | N/A | Peripheral blood |
| C11 | 58/M | Colorectal cancer | II | Peripheral blood |
| C12 | 72/M | Colorectal cancer | I | Peripheral blood |
| C13 | 59/M | Colorectal cancer | II | Peripheral blood |
| C14 | 72/M | Colorectal cancer | I | Peripheral blood |
| C15 | 78/M | Colorectal cancer | N/A | Peripheral blood |
| C16 | 68/M | Colorectal cancer | II | Peripheral blood |
| C17 | 48/M | Colorectal cancer | II | Peripheral blood |
| C18 | 33/W | Colorectal cancer | II | Peripheral blood |
| C19 | 43/M | Colorectal cancer | III | Peripheral blood |
| C20 | 48/W | Colorectal cancer | II | Peripheral blood |
| C21 | 65/M | Colorectal cancer | I | Peripheral blood |
| C22 | 71/M | Colorectal cancer | II | Peripheral blood |
| C23 | 68/M | Colorectal cancer | II | Peripheral blood |
| C24 | 67/M | Colorectal cancer | II | Peripheral blood |
| C25 | 74/M | Colorectal cancer | II | Peripheral blood |
| C26 | 72/M | Colorectal cancer | III | Peripheral blood |
| C27 | 77/M | Colorectal cancer | II | Peripheral blood |
| L1 | 60/M | Lung cancer | IV | Peripheral blood |
| L2 | 60/M | Lung cancer | IV | Peripheral blood |

**Table S2: Comparison between the CytoExam and the gold standard technique for CTC detection.**

| Instrument | CytoExam | CellSearch^8^ |
| --- | --- | --- |
| Technique | Inertial microfluidics & impedance cytometry | Immune-affinity |
| Consumables | eScan-chip | Antibody reagents, staining reagents, and magnetic beads, etc. |
| Cell viability | High cell viability (~98%) | Dead cells |
| Material cost | ~10 $ for each sample | ~400 $ for each sample |
| Classification method | Impedance detection; automatic classification using algorithms | Fluorescence enumeration;  classification according to image data; relying on human experience for judgment |
| Time-consuming | < 15 min for sample processing, and ~30 min for data analysis | ~48 h for result acquisition |


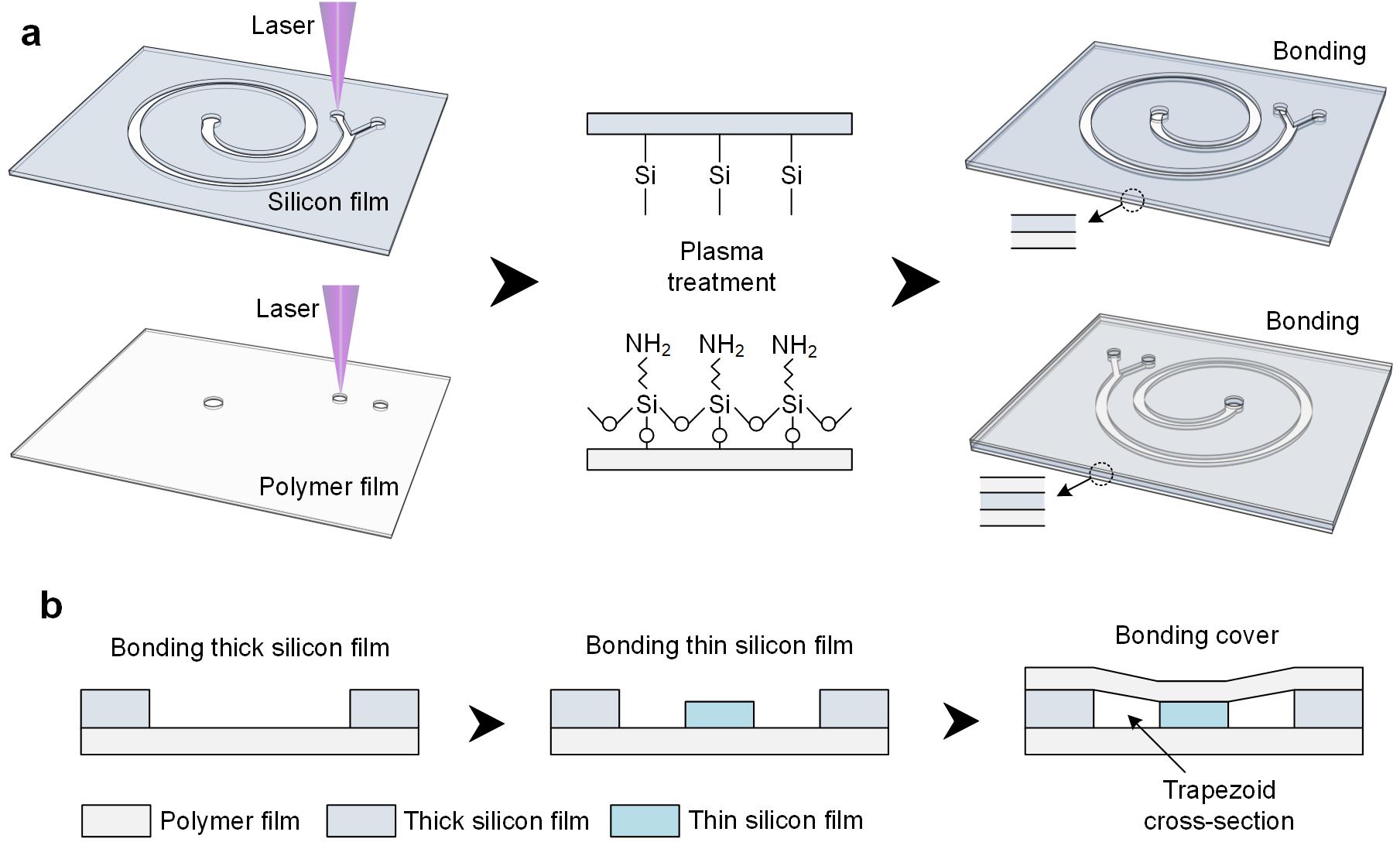


**Fig. S32: Schematics of film-chip manufacturing technology. a,** Fabrication of curved channel featured with rectangle cross-section. **b,** Fabrication of curved channel featured with trapezoidal cross-section.


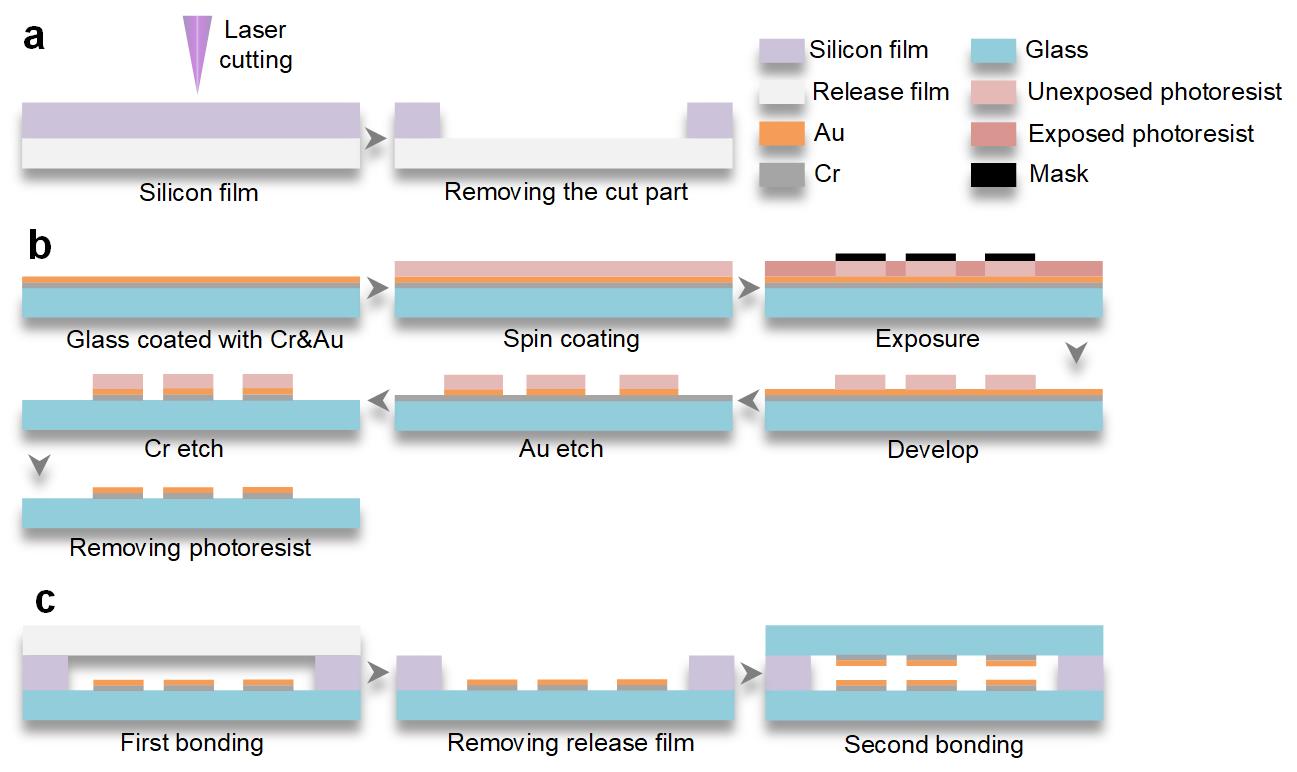


**Fig. S33: Schematics for fabricating impedance cytometry with face-to-face electrodes. a,** Channel fabrication using laser cutting technology. **b,** Electrode fabrication using photolithography technology. **c,** Assemble of the impedance cytometry by bonding channel and another electrode plate precisely.


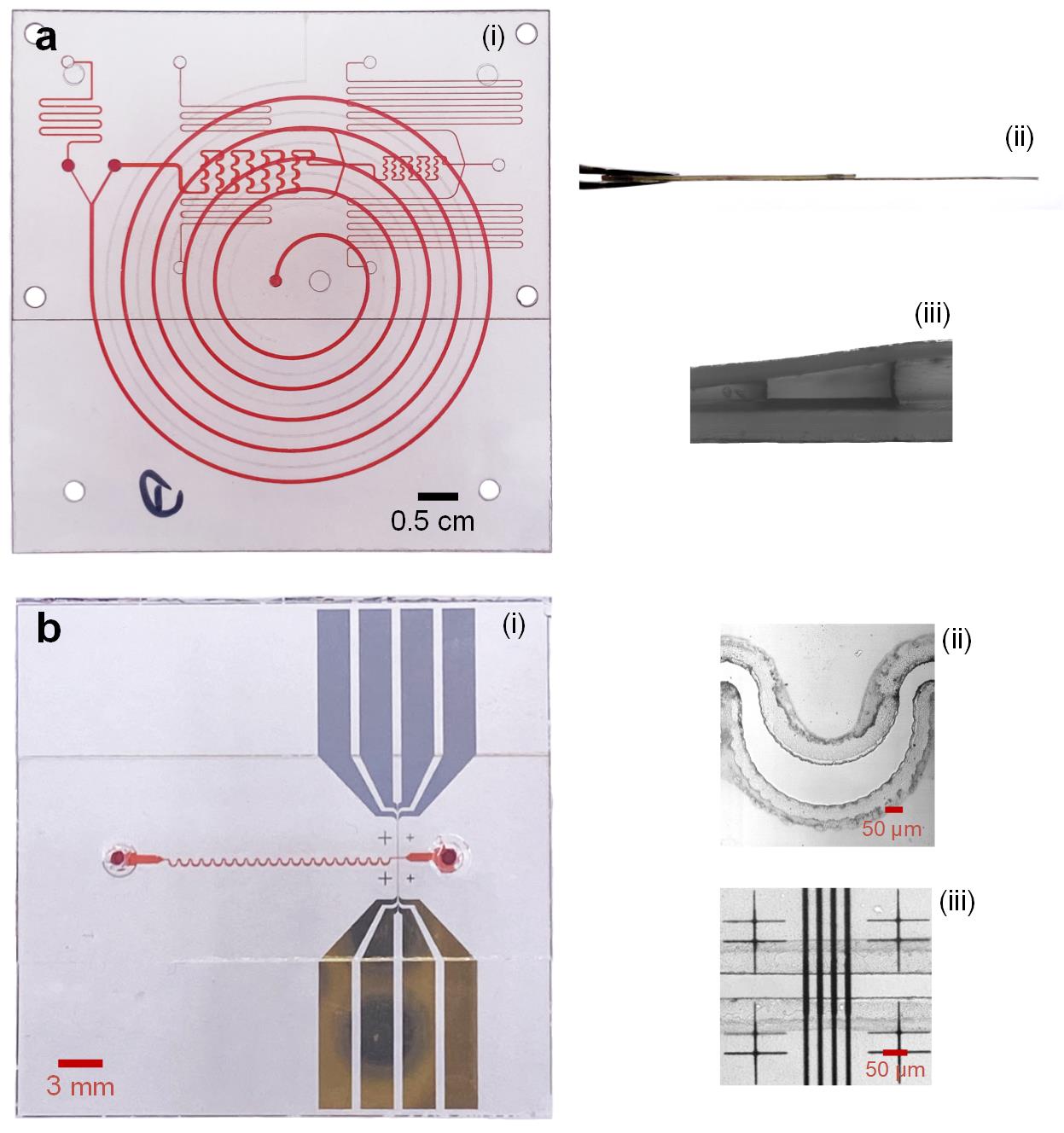


**Fig. S34: Images of each fabricated module. a,** Front view (i) and side view (ii) of the separation and concentration modules, microscopic image of the trapezoidal cross-section (iii) in separation module. **b,** Front view (i) of the detection module, microscopic images of the asymmetric serpentine channel (ii), and four pairs of face-to-face electrodes (iii). Each module is filled with red ink for clear visualization.


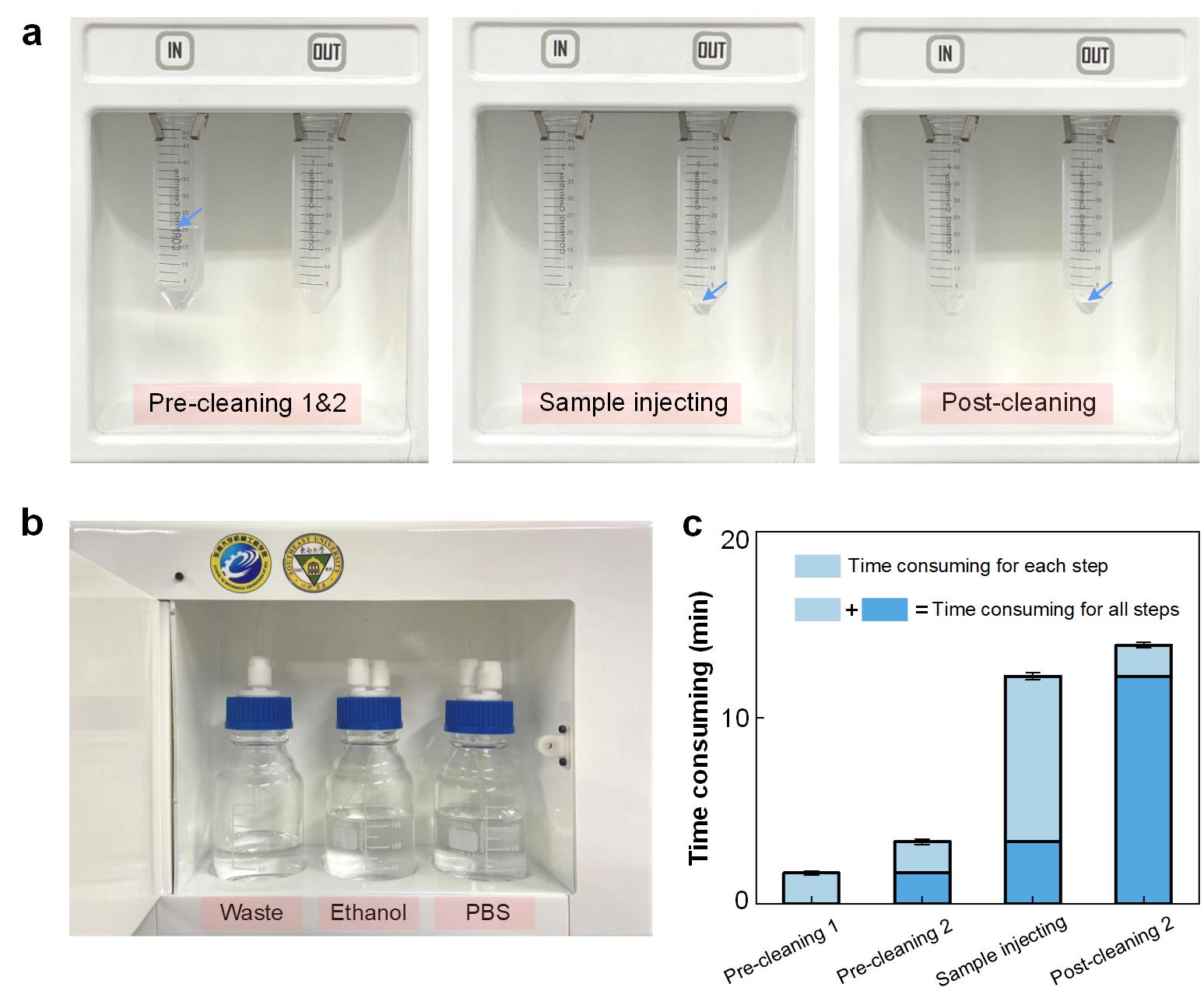


**Fig. S35: Testing of the instrument. a,** Changes of liquid level in centrifuge tubes when the instrument performing pre-cleaning 1&2, sample injecting, and post-cleaning steps. **b,** Storage locations of the waste, ethanol, and PBS. **b,** Time consuming when the instrument performing each step.

**Reference**

1. Zhu, Z. X.; Wu, D.; Li, S., et al., A polymer-film inertial microfluidic sorter fabricated by jigsaw puzzle method for precise size-based cell separation. *Analytica Chimica Acta* **2021,** *1143*, 306-314.

2. Murlidhar, V.; Rivera-Baez, L.; Nagrath, S., Affinity Versus Label-Free Isolation of Circulating Tumor Cells: Who Wins? *Small* **2016,** *12* (33), 4450-4463.

3. Jack, R. M.; Grafton, M. M.; Rodrigues, D., et al., Ultra-Specific Isolation of Circulating Tumor Cells Enables Rare-Cell RNA Profiling. *Advanced Science* **2016,** *3* (9), 1600063.

4. Leung, C. H.; Wu, K. J.; Li, G., et al., Application of label-free techniques in microfluidic for biomolecules detection and circulating tumor cells analysis. *TrAC Trends in Analytical Chemistry* **2019,** *117*, 78-83.

5. Fang, Y. H.; Zhu, S.; Cheng, W. Q., et al., Efficient bioparticle extraction using a miniaturized inertial microfluidic centrifuge. *Lab on a Chip* **2022,** *22* (18), 3545-3554.

6. Caselli, F.; De Ninno, A.; Reale, R., et al., A novel wiring scheme for standard chips enabling high-accuracy impedance cytometry. *Sensors and Actuators B: Chemical* **2018,** *256*, 580-589.

7. Spencer, D. C.; Paton, T. F.; Mulroney, K. T., et al., A fast impedance-based antimicrobial susceptibility test. *Nature Communications* **2020,** *11* (1), 5328.

8. Almici, C.; Neva, A.; Skert, C., et al., Counting circulating endothelial cells in allo-HSCT: an ad hoc designed polychromatic flowcytometry-based panel versus the CellSearch System. *Scientific Reports* **2019,** *9*, 87.
