# Supplementary figures and images for: Liquid Biopsy Instrument for Ultra-Fast and Label-Free Detection of Circulating Tumor Cells
Source: Research (Wash D C). 2024 Jul 24;7:0431. doi: 10.34133/research.0431 (PMC11266806; doi:10.34133/research.0431)

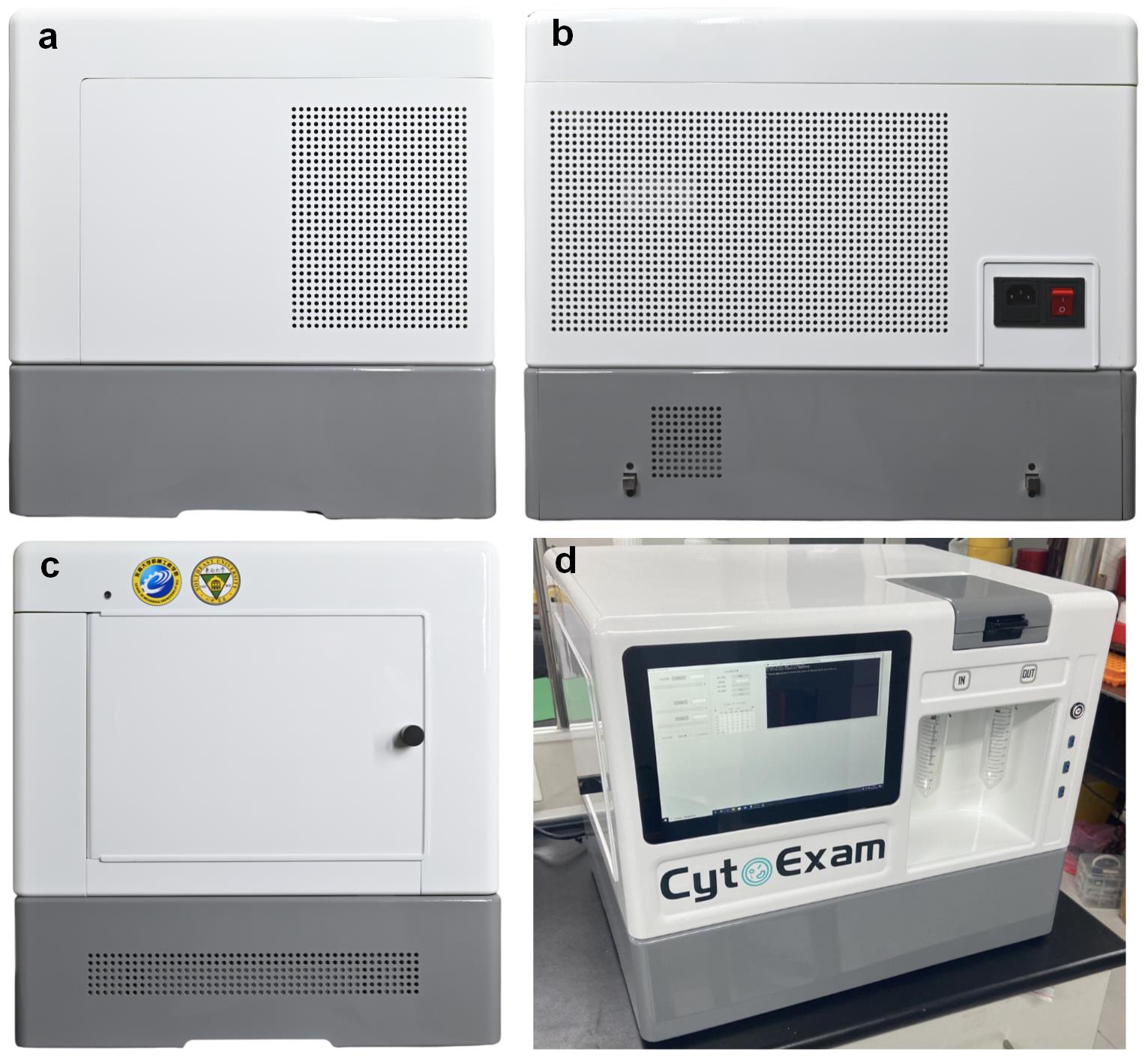

Supplement: Supplementary 1 — Figs. S1 to S35 Table S1 and S2 [file research.0431.f1.zip › Fig.S1.jpg]

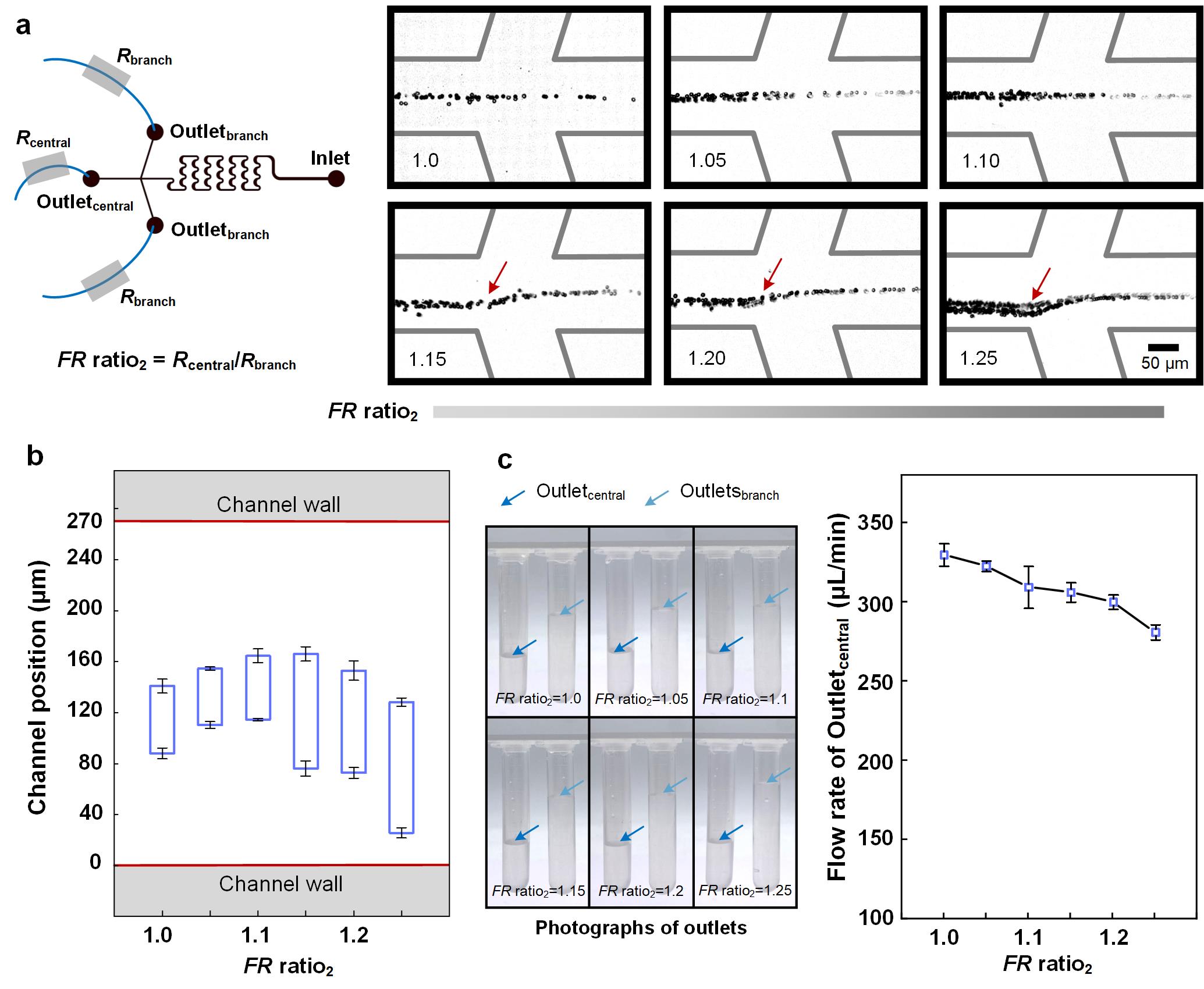

Supplement: Supplementary 1 — Figs. S1 to S35 Table S1 and S2 [file research.0431.f1.zip › Fig.S10.jpg]

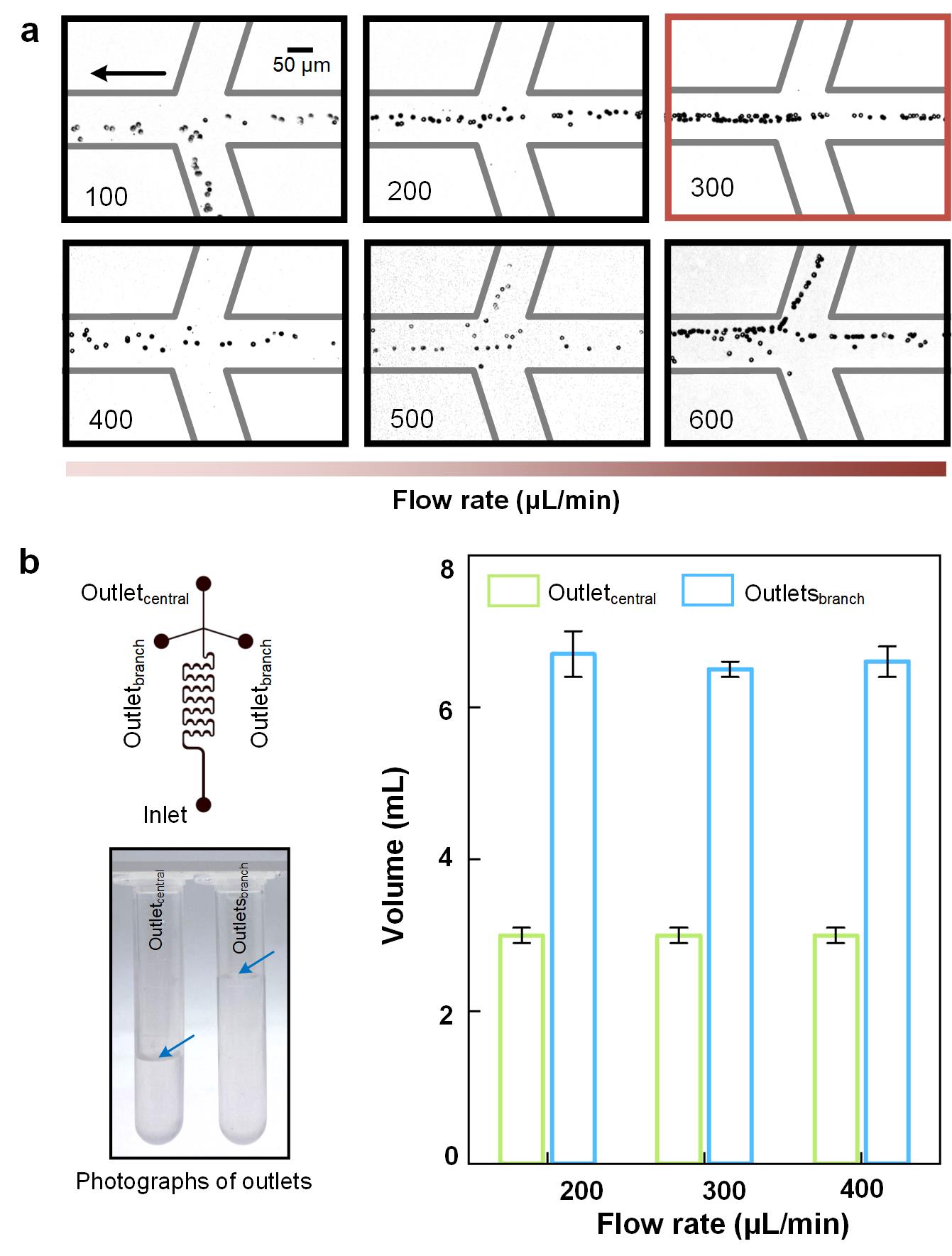

Supplement: Supplementary 1 — Figs. S1 to S35 Table S1 and S2 [file research.0431.f1.zip › Fig.S11.jpg]

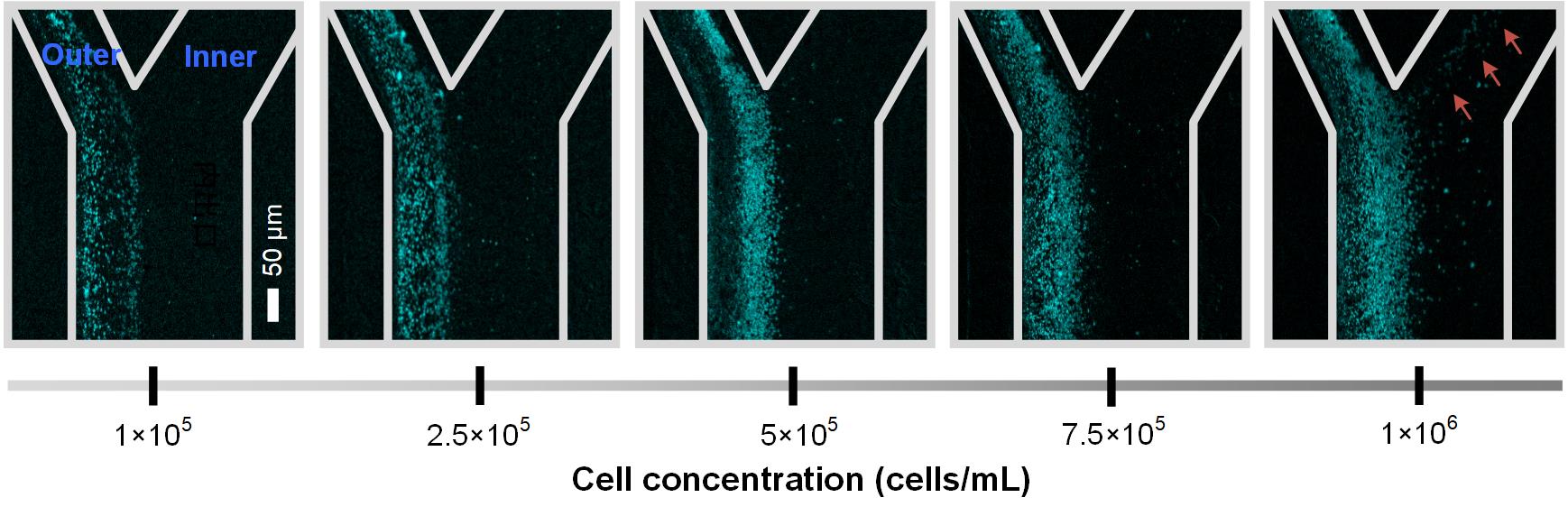

Supplement: Supplementary 1 — Figs. S1 to S35 Table S1 and S2 [file research.0431.f1.zip › Fig.S12.jpg]

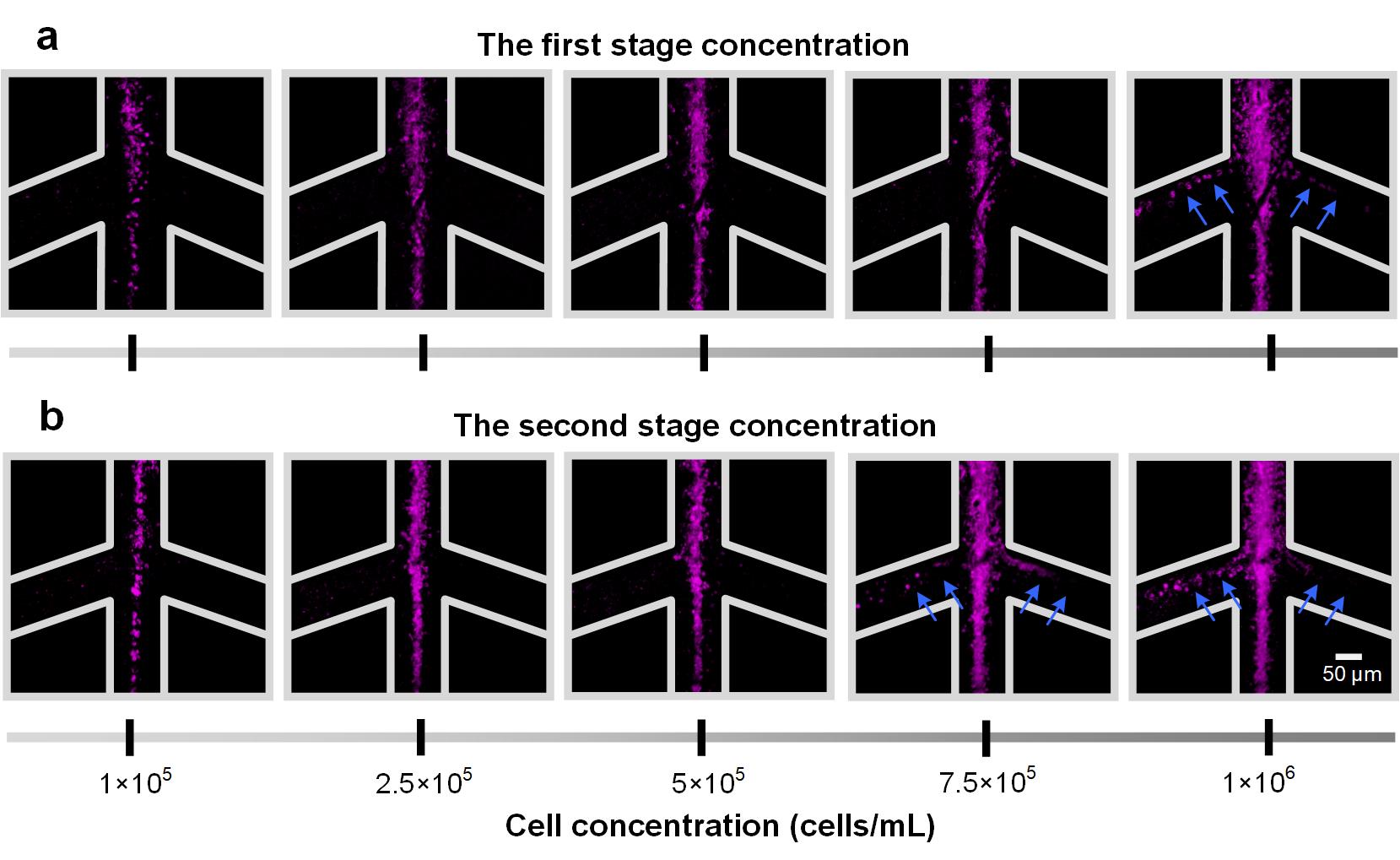

Supplement: Supplementary 1 — Figs. S1 to S35 Table S1 and S2 [file research.0431.f1.zip › Fig.S13.jpg]

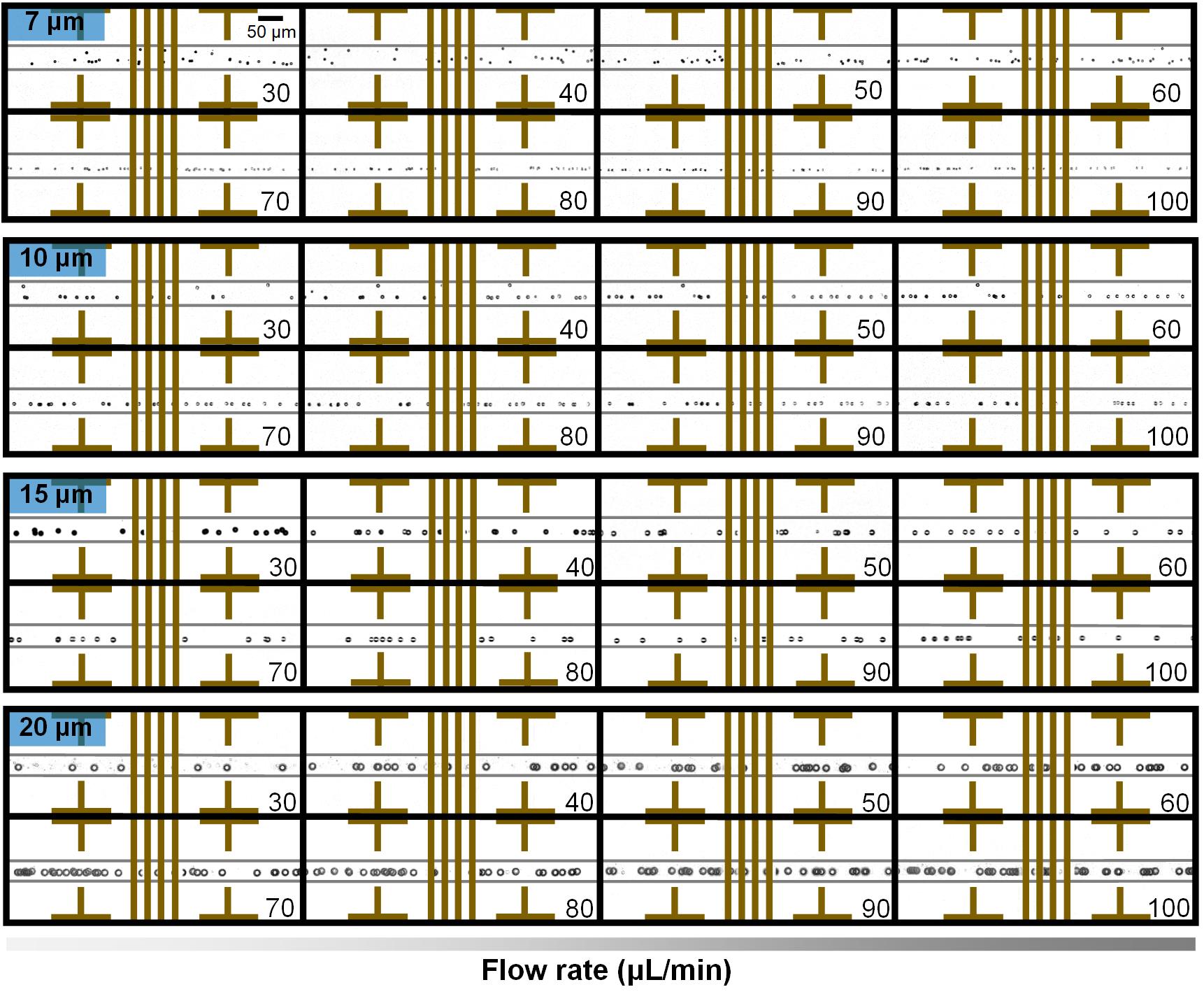

Supplement: Supplementary 1 — Figs. S1 to S35 Table S1 and S2 [file research.0431.f1.zip › Fig.S14.jpg]

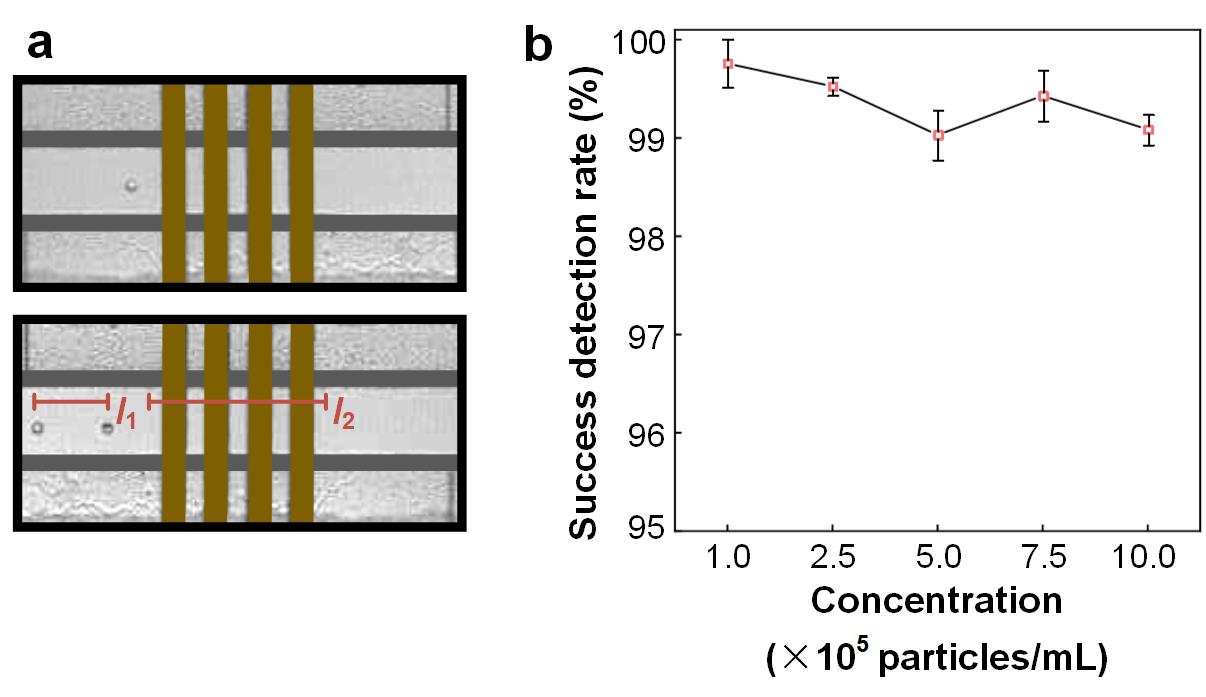

Supplement: Supplementary 1 — Figs. S1 to S35 Table S1 and S2 [file research.0431.f1.zip › Fig.S15.jpg]

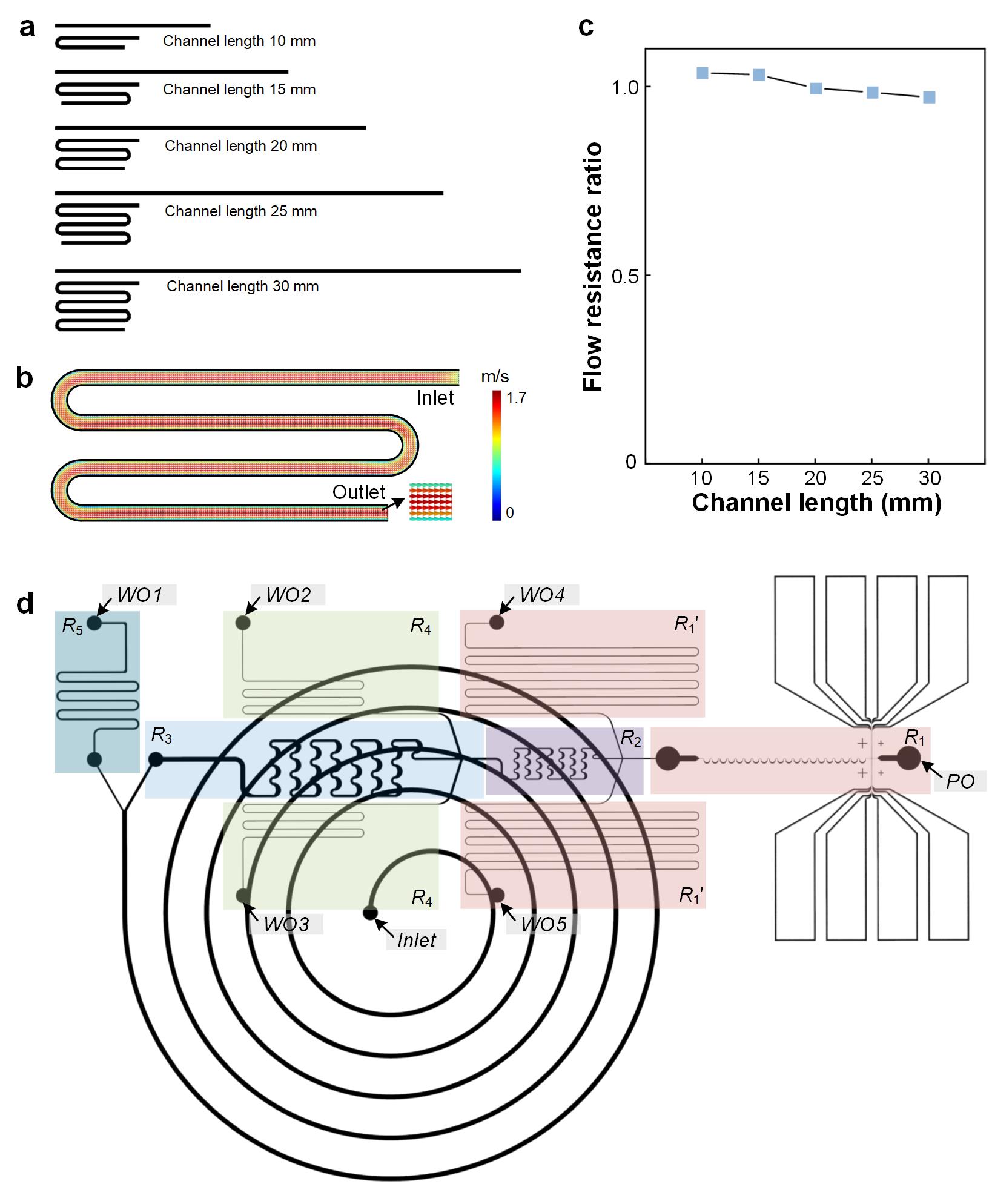

Supplement: Supplementary 1 — Figs. S1 to S35 Table S1 and S2 [file research.0431.f1.zip › Fig.S16.jpg]

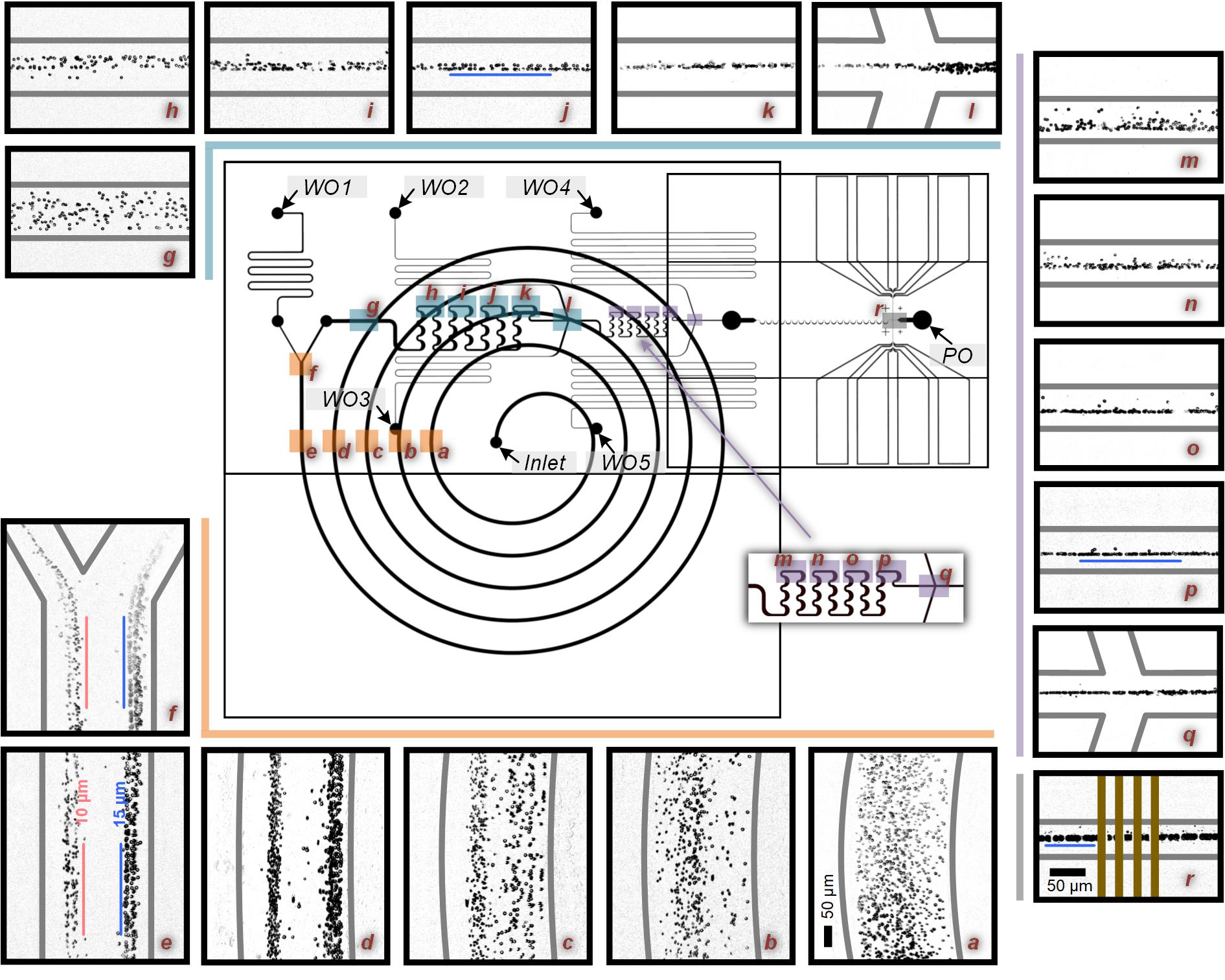

Supplement: Supplementary 1 — Figs. S1 to S35 Table S1 and S2 [file research.0431.f1.zip › Fig.S17.jpg]

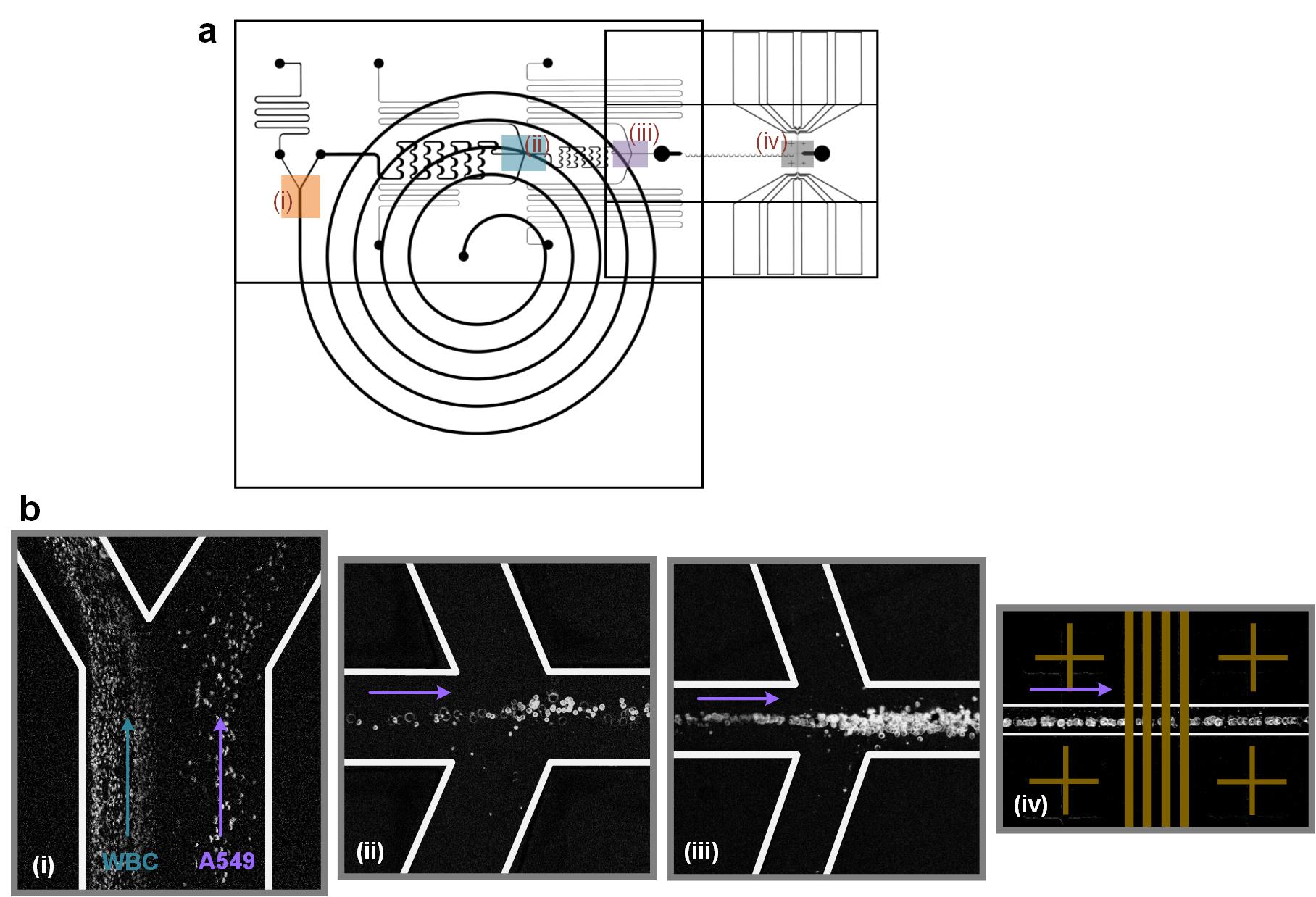

Supplement: Supplementary 1 — Figs. S1 to S35 Table S1 and S2 [file research.0431.f1.zip › Fig.S18.jpg]

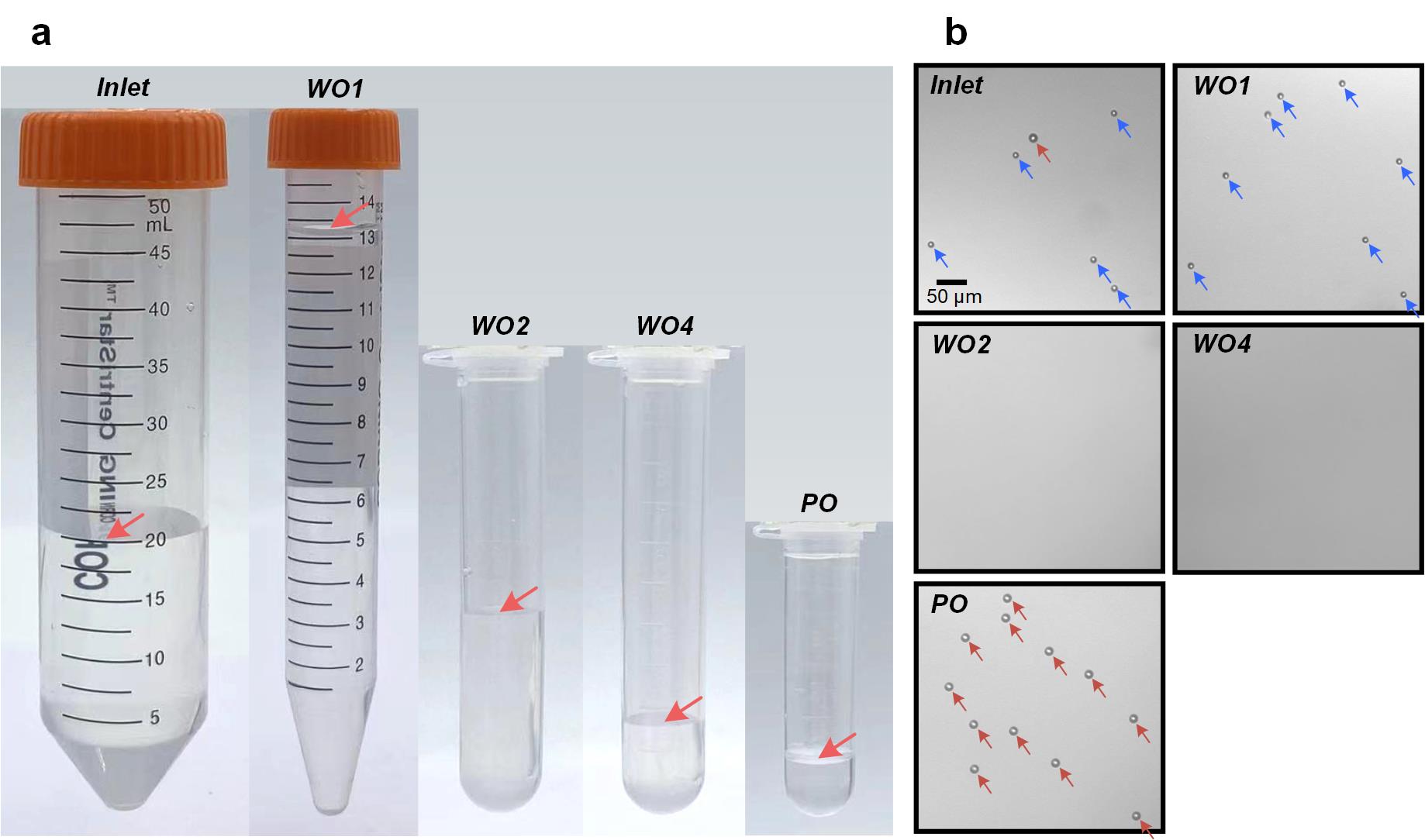

Supplement: Supplementary 1 — Figs. S1 to S35 Table S1 and S2 [file research.0431.f1.zip › Fig.S19.jpg]

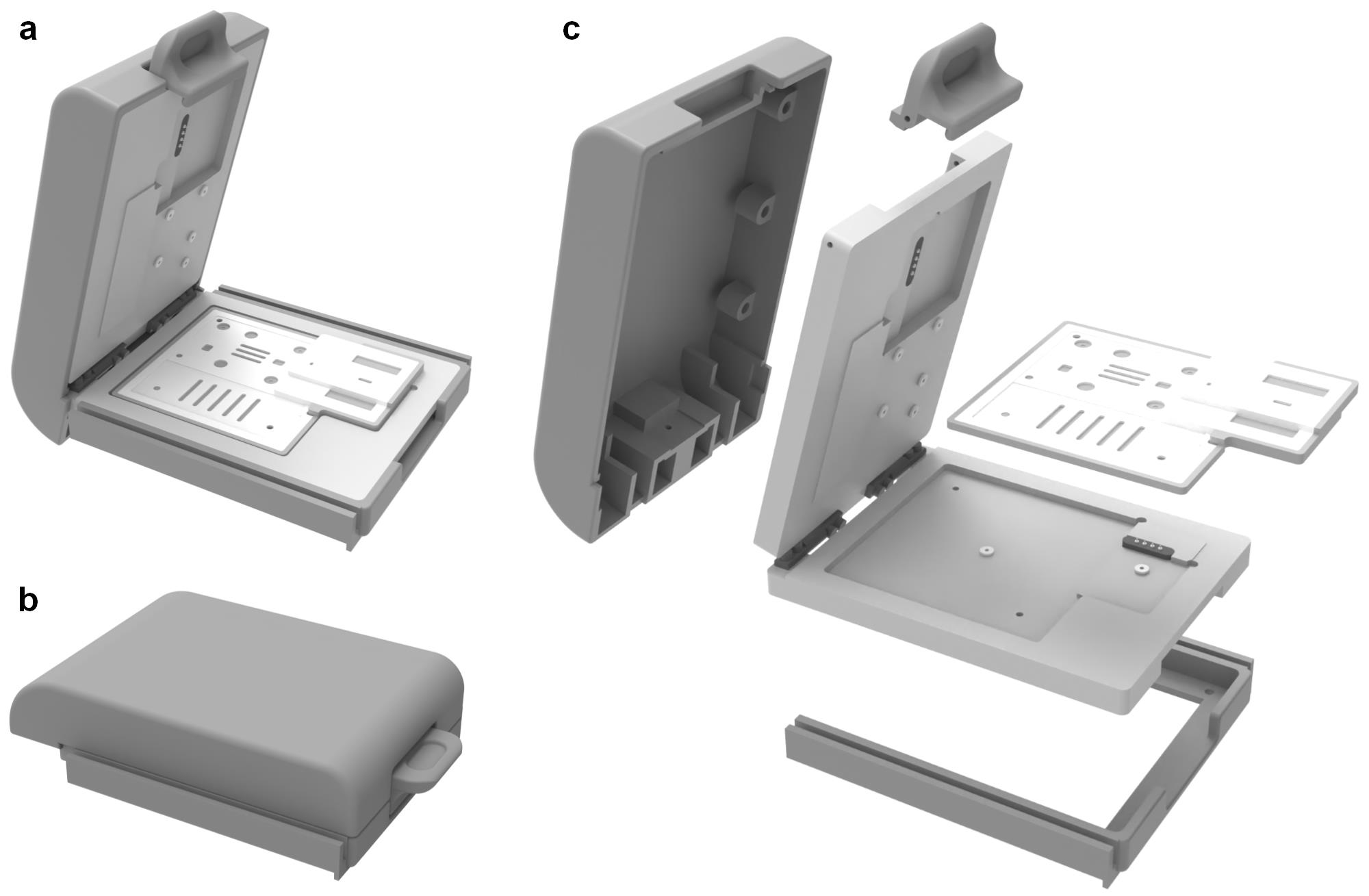

Supplement: Supplementary 1 — Figs. S1 to S35 Table S1 and S2 [file research.0431.f1.zip › Fig.S2.jpg]

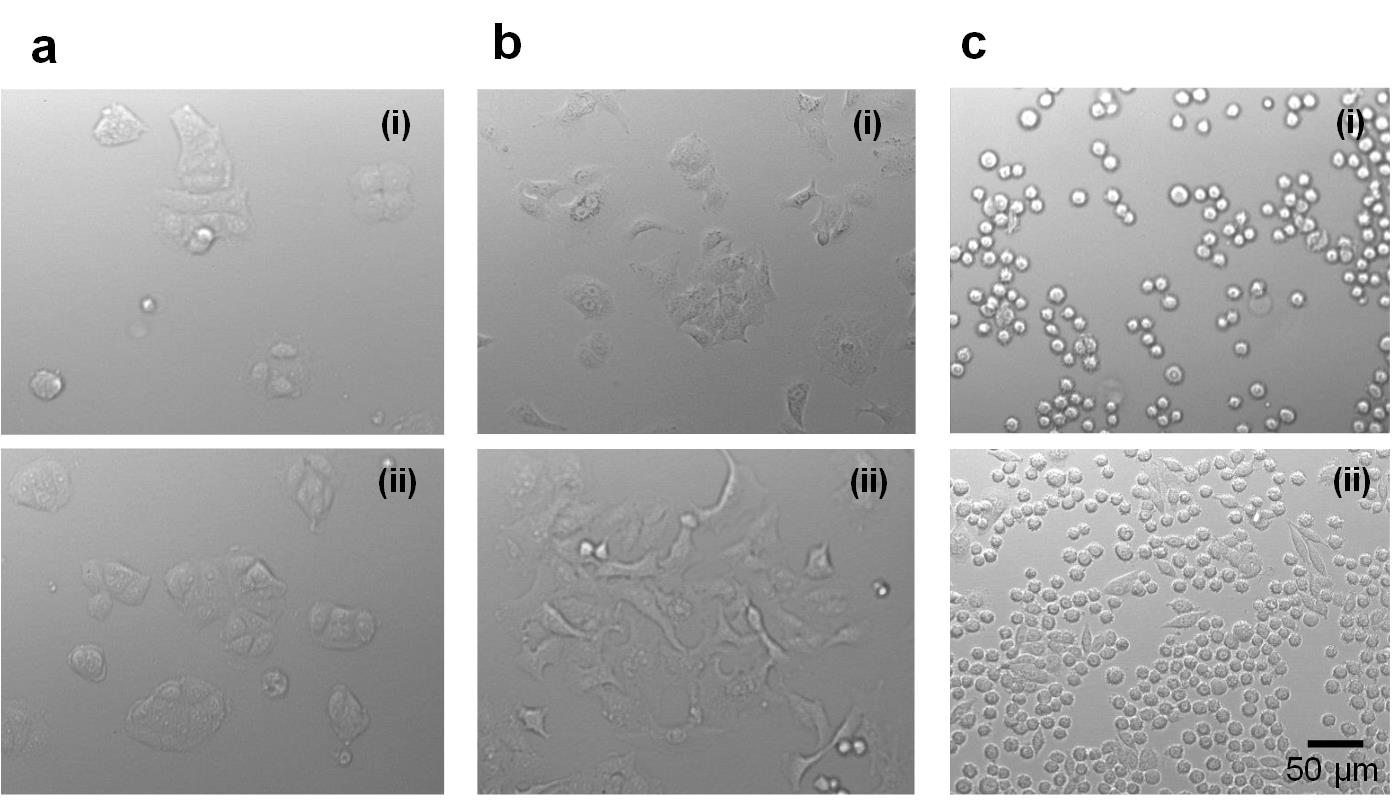

Supplement: Supplementary 1 — Figs. S1 to S35 Table S1 and S2 [file research.0431.f1.zip › Fig.S20.jpg]

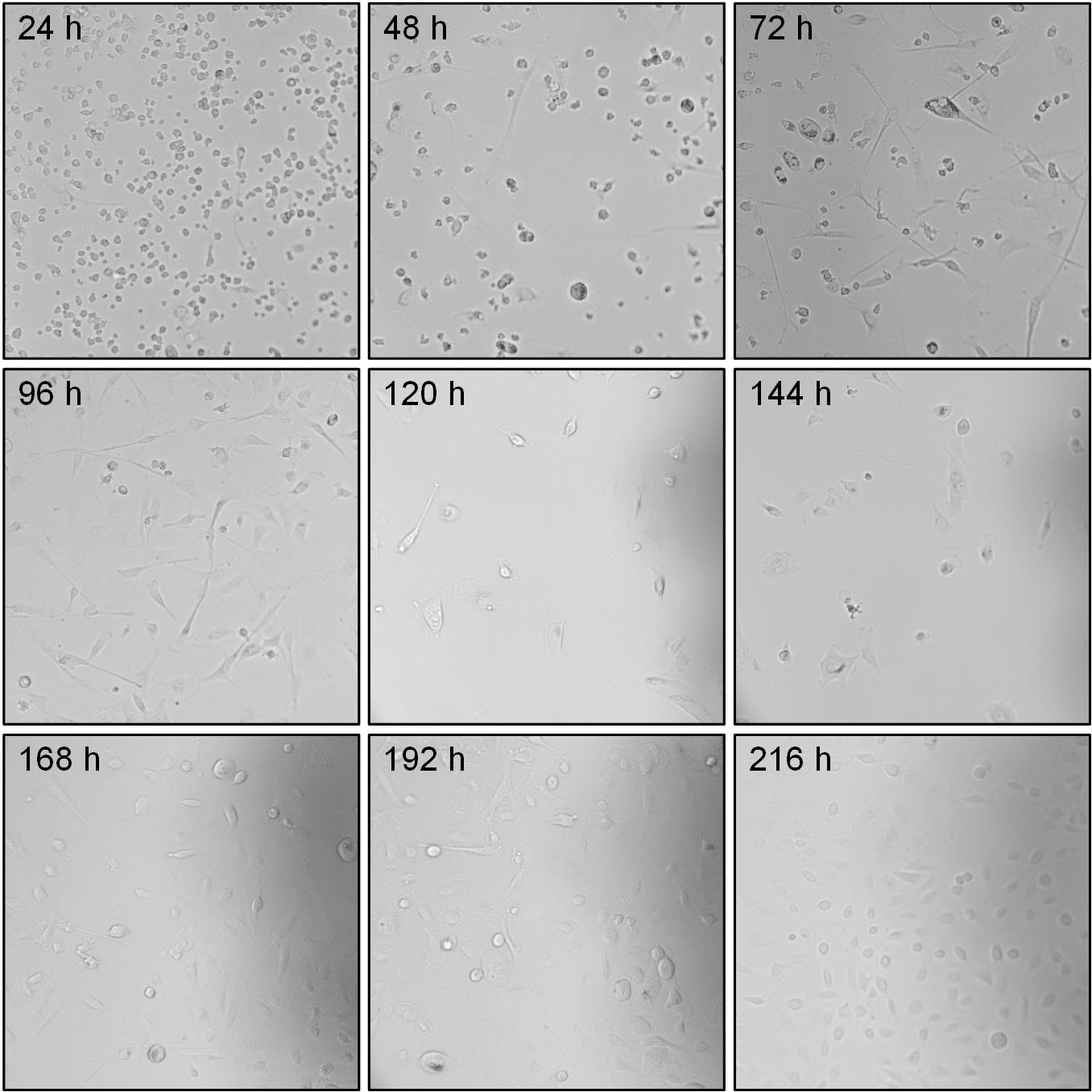

Supplement: Supplementary 1 — Figs. S1 to S35 Table S1 and S2 [file research.0431.f1.zip › Fig.S21.jpg]

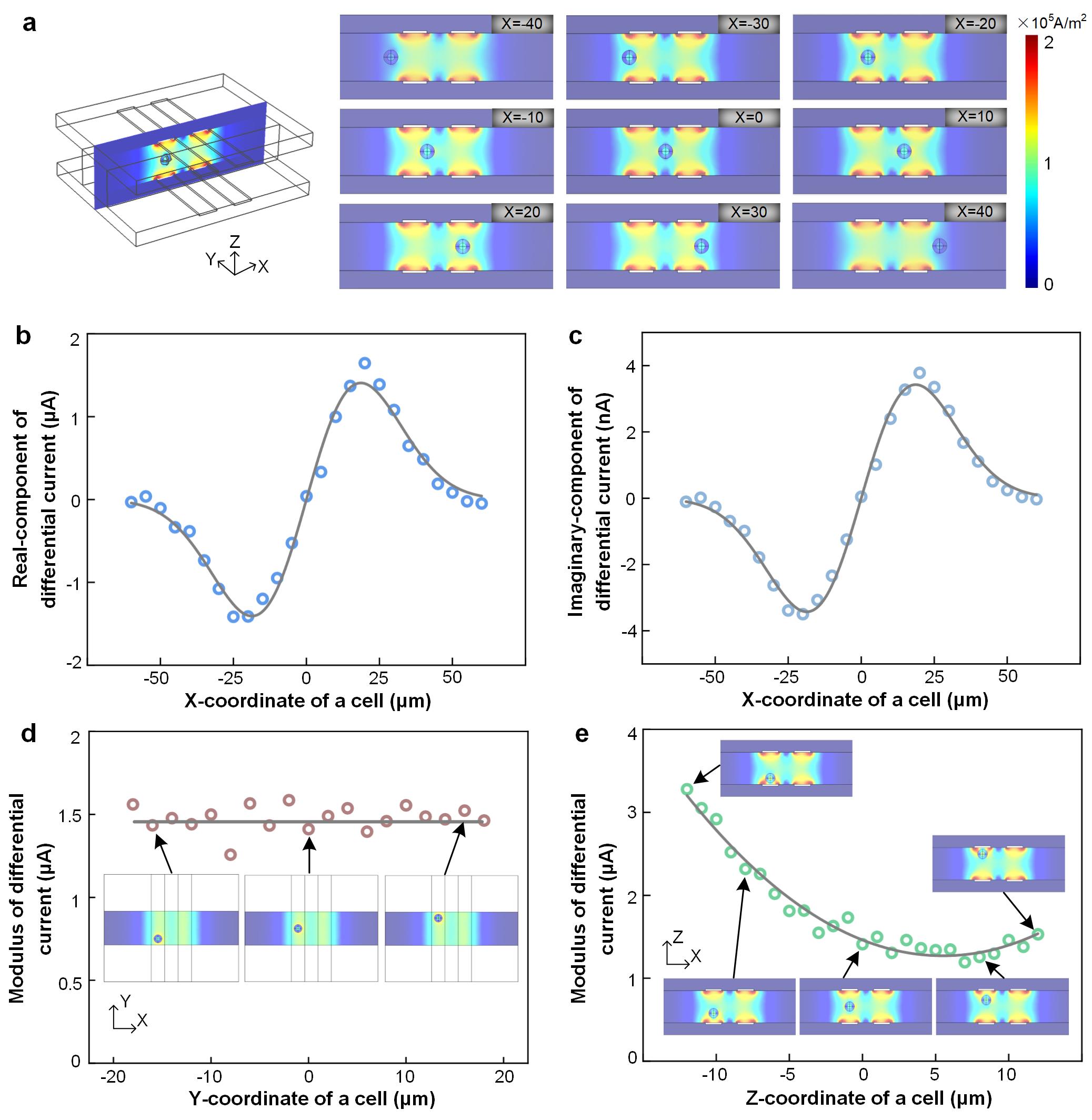

Supplement: Supplementary 1 — Figs. S1 to S35 Table S1 and S2 [file research.0431.f1.zip › Fig.S22.jpg]

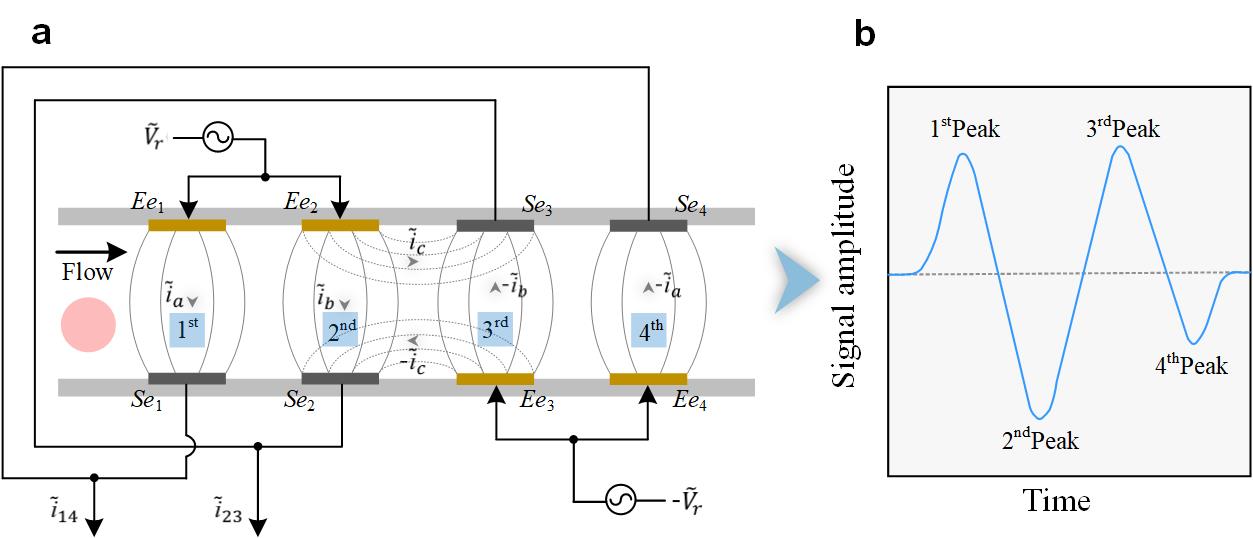

Supplement: Supplementary 1 — Figs. S1 to S35 Table S1 and S2 [file research.0431.f1.zip › Fig.S23.jpg]

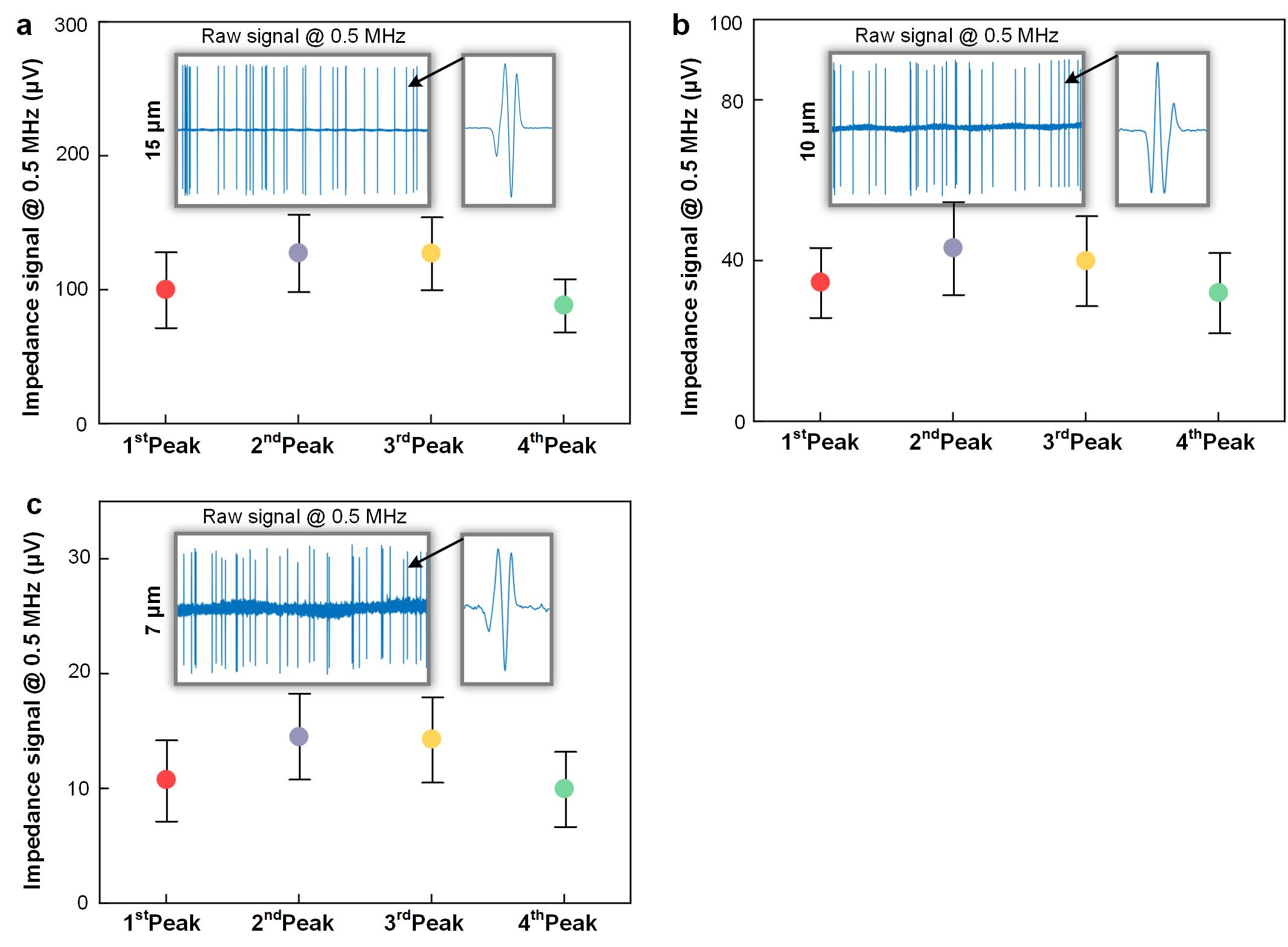

Supplement: Supplementary 1 — Figs. S1 to S35 Table S1 and S2 [file research.0431.f1.zip › Fig.S24.jpg]

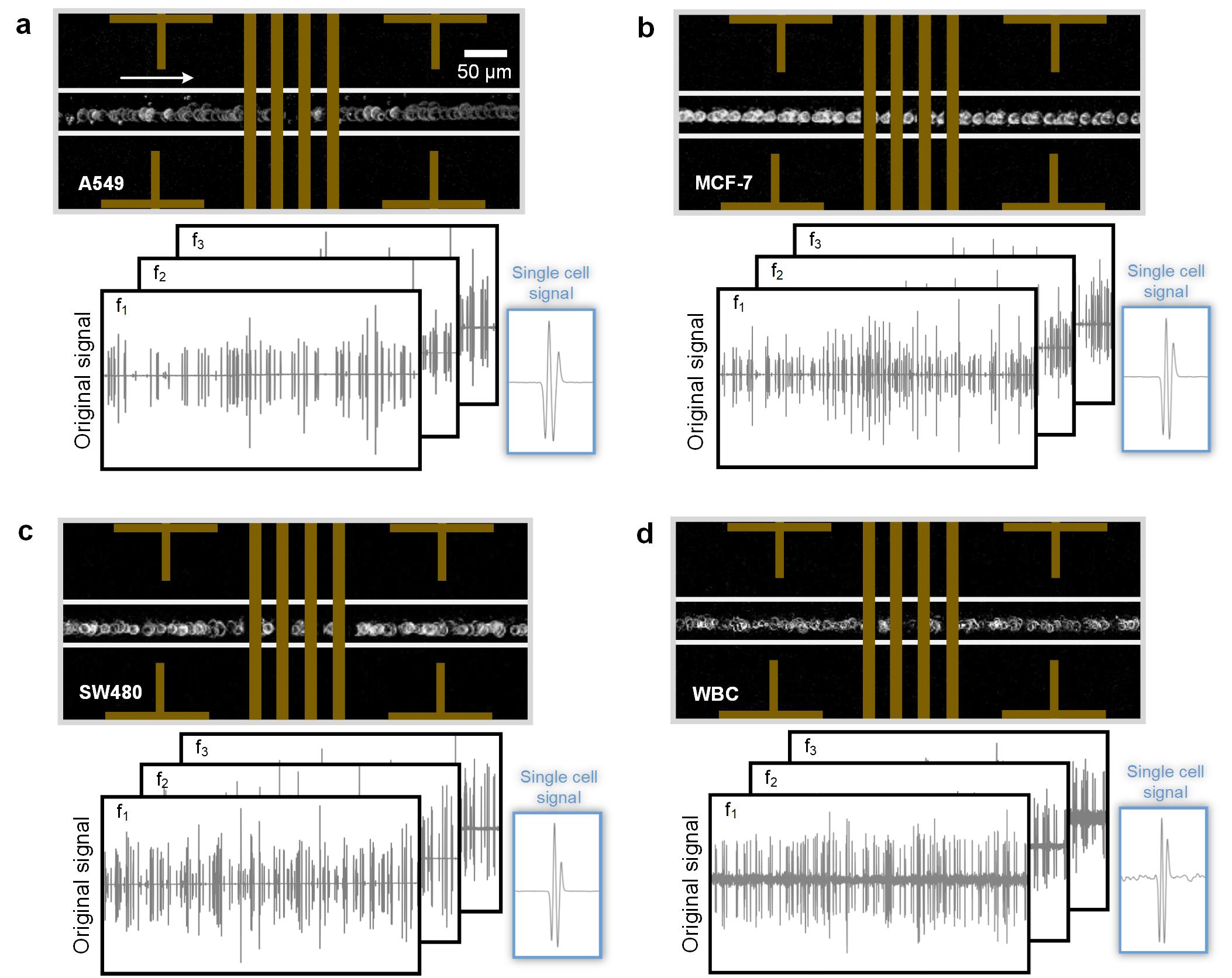

Supplement: Supplementary 1 — Figs. S1 to S35 Table S1 and S2 [file research.0431.f1.zip › Fig.S25.jpg]

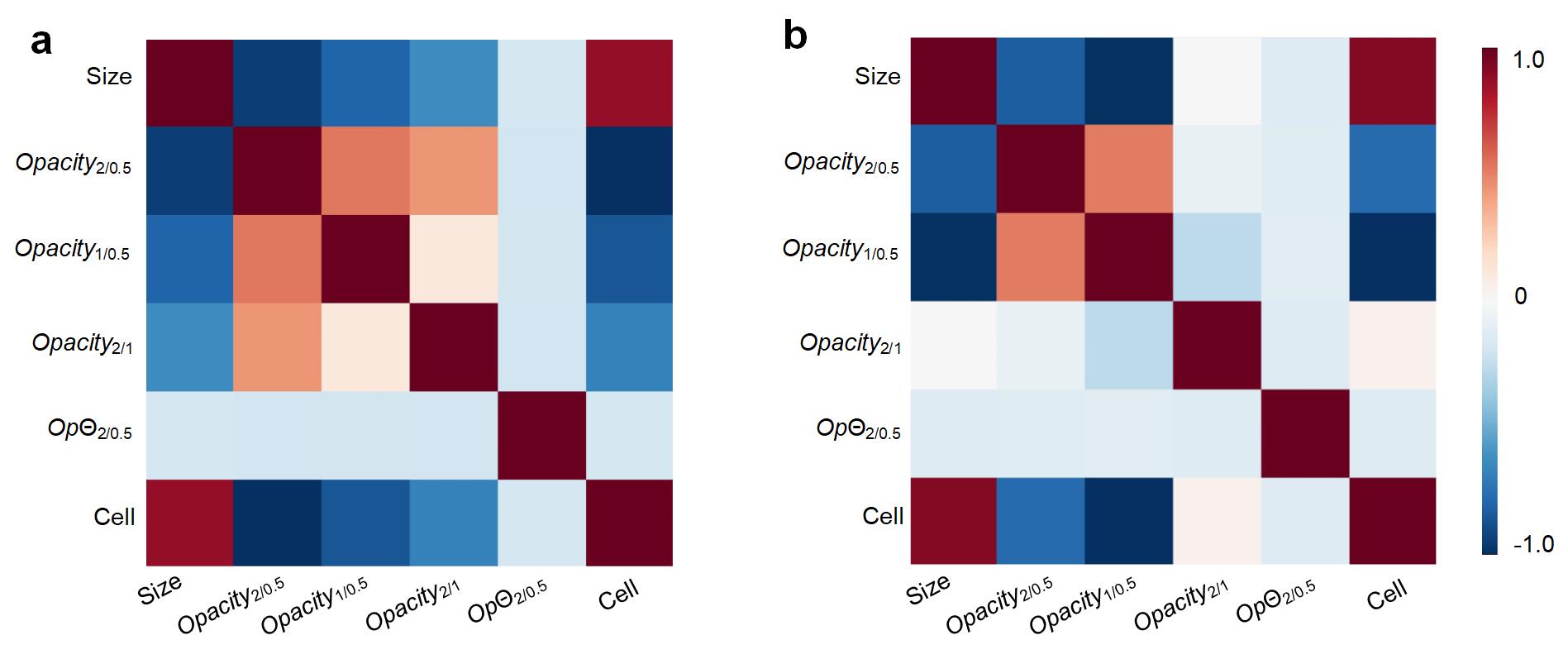

Supplement: Supplementary 1 — Figs. S1 to S35 Table S1 and S2 [file research.0431.f1.zip › Fig.S26.jpg]

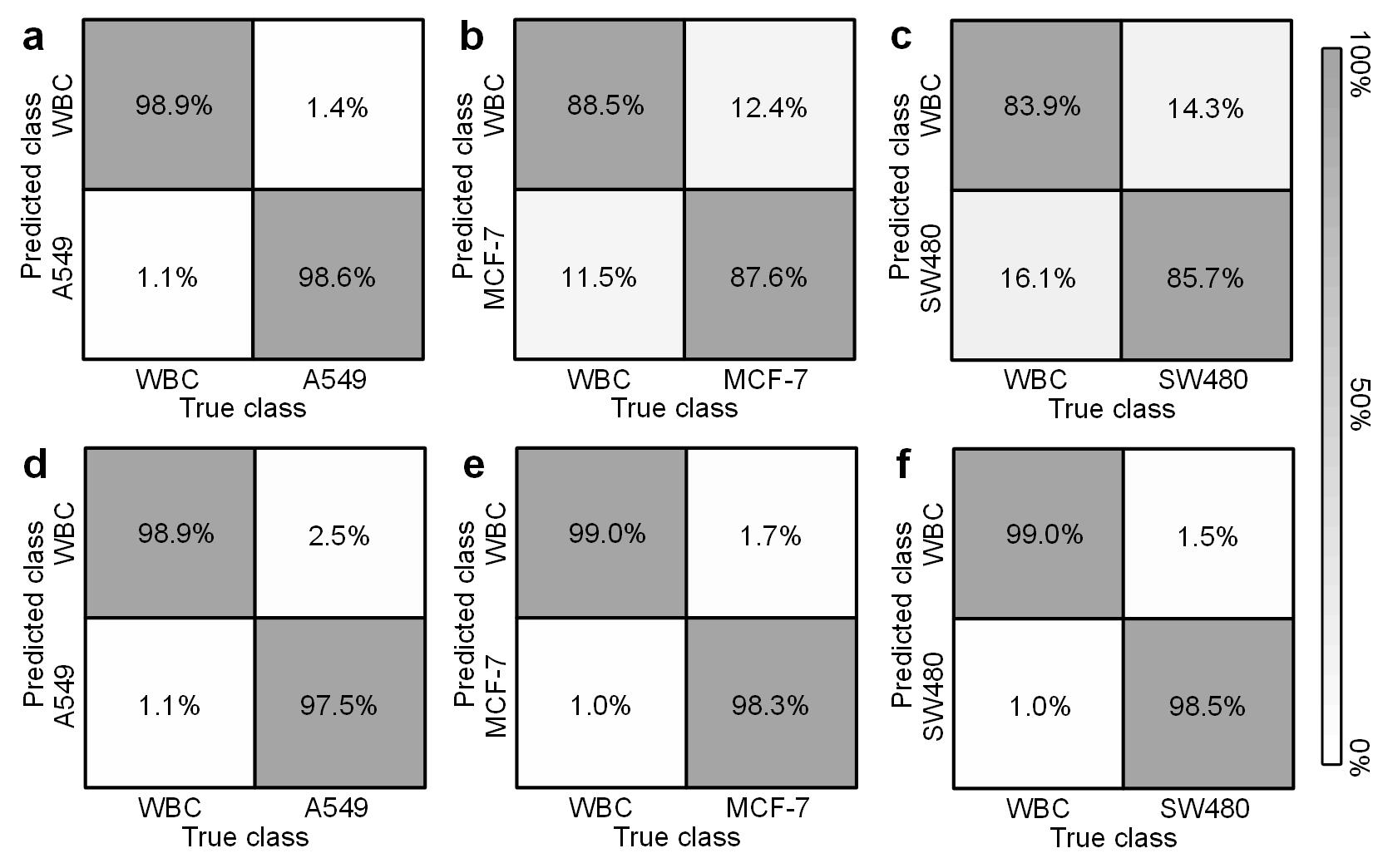

Supplement: Supplementary 1 — Figs. S1 to S35 Table S1 and S2 [file research.0431.f1.zip › Fig.S27.jpg]

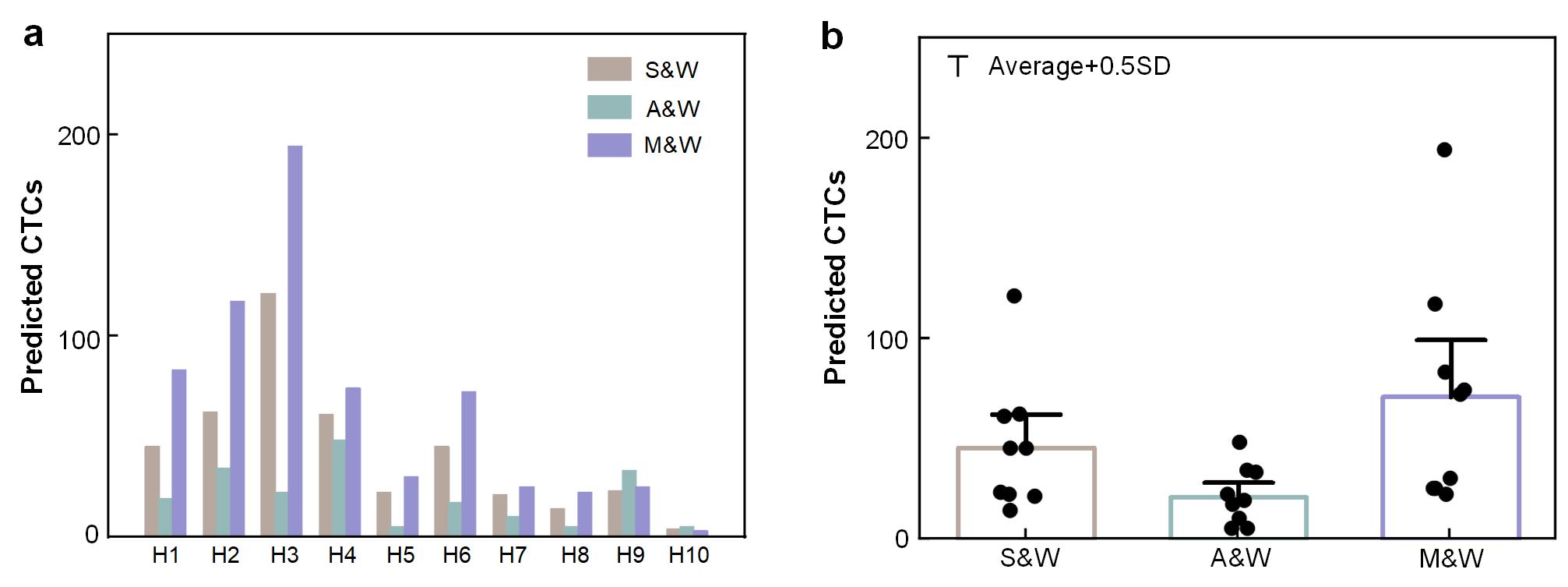

Supplement: Supplementary 1 — Figs. S1 to S35 Table S1 and S2 [file research.0431.f1.zip › Fig.S28.jpg]

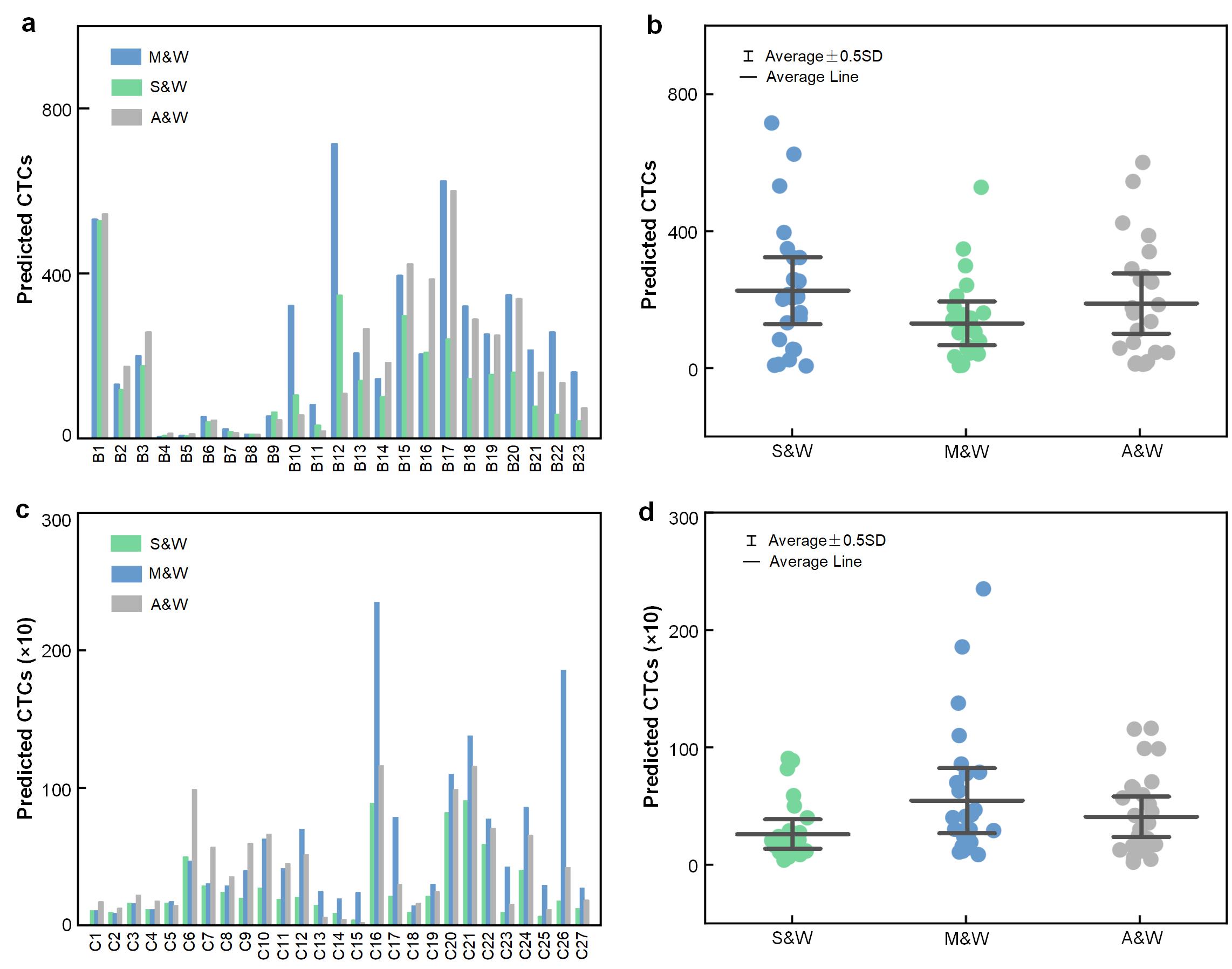

Supplement: Supplementary 1 — Figs. S1 to S35 Table S1 and S2 [file research.0431.f1.zip › Fig.S29.jpg]

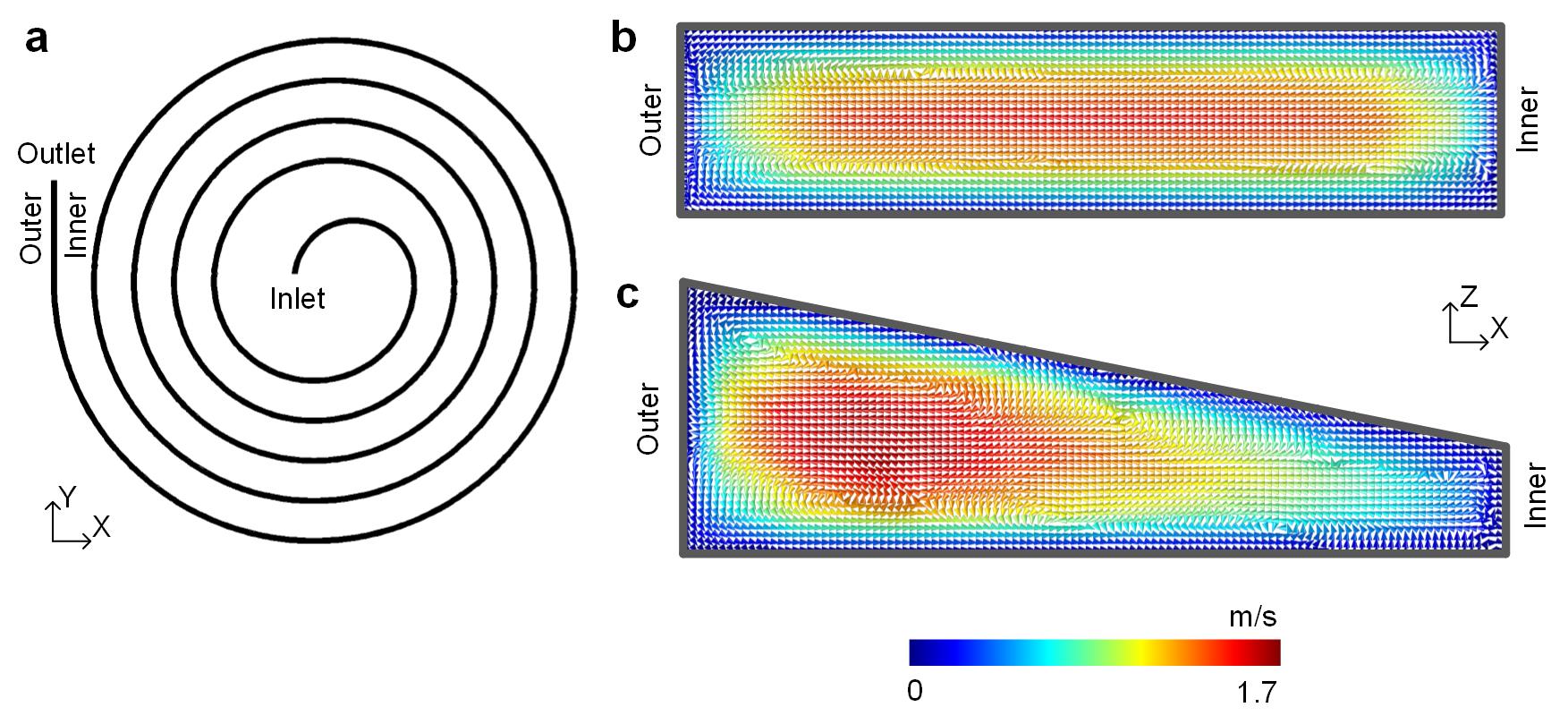

Supplement: Supplementary 1 — Figs. S1 to S35 Table S1 and S2 [file research.0431.f1.zip › Fig.S3.jpg]

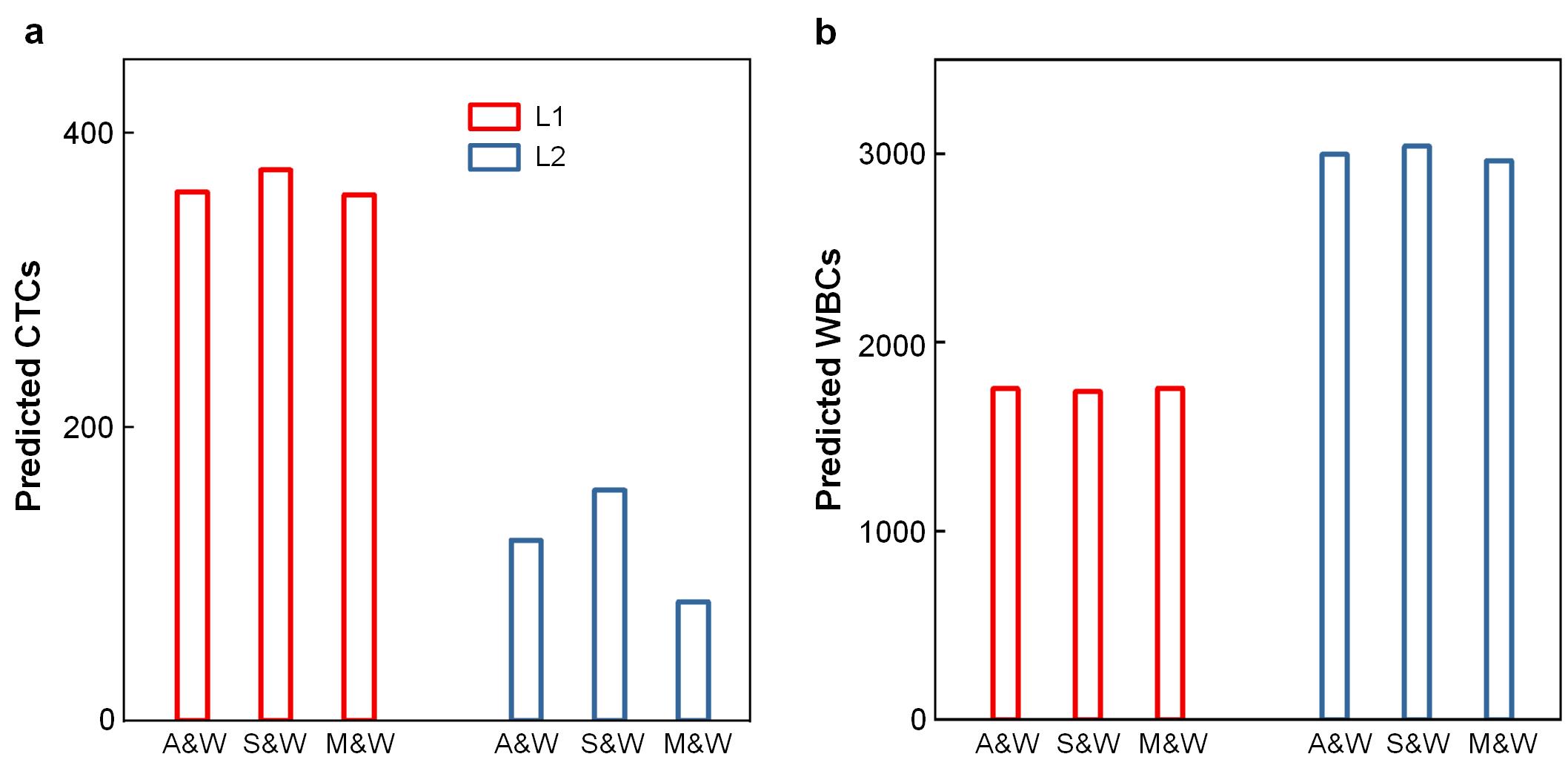

Supplement: Supplementary 1 — Figs. S1 to S35 Table S1 and S2 [file research.0431.f1.zip › Fig.S30.jpg]

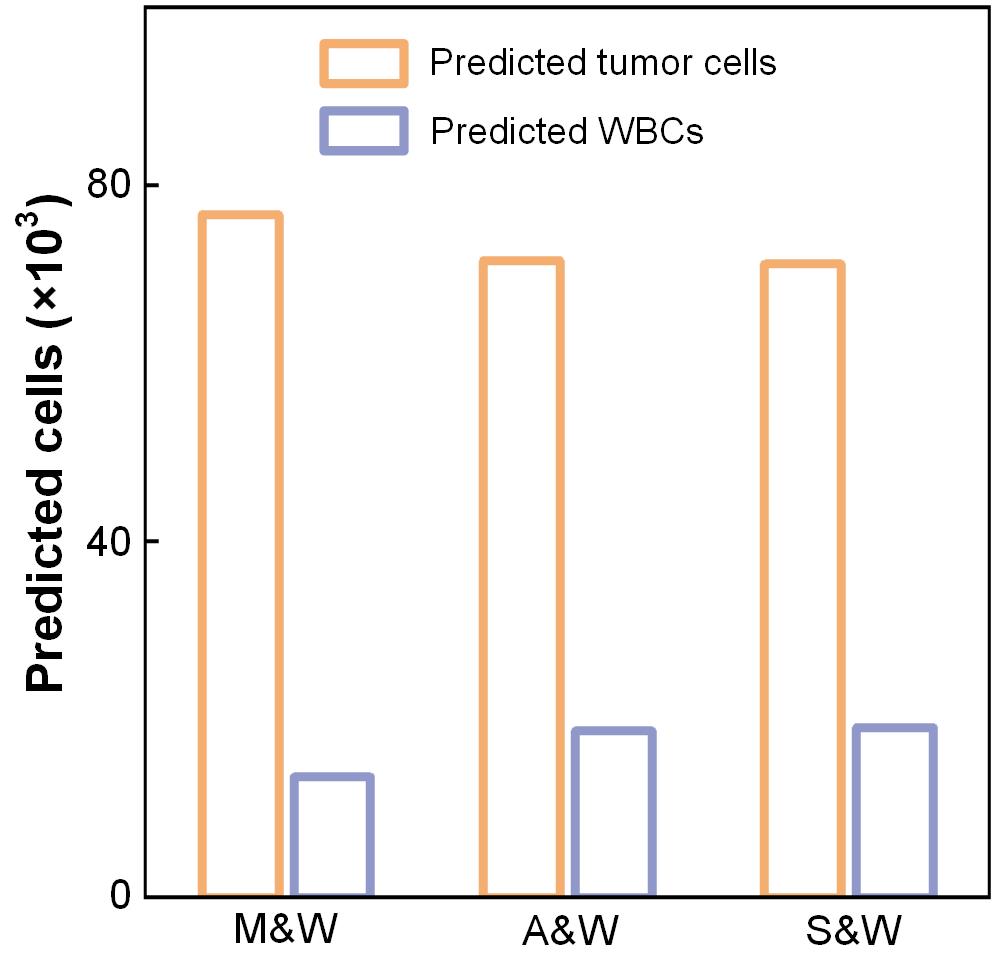

Supplement: Supplementary 1 — Figs. S1 to S35 Table S1 and S2 [file research.0431.f1.zip › Fig.S31.jpg]

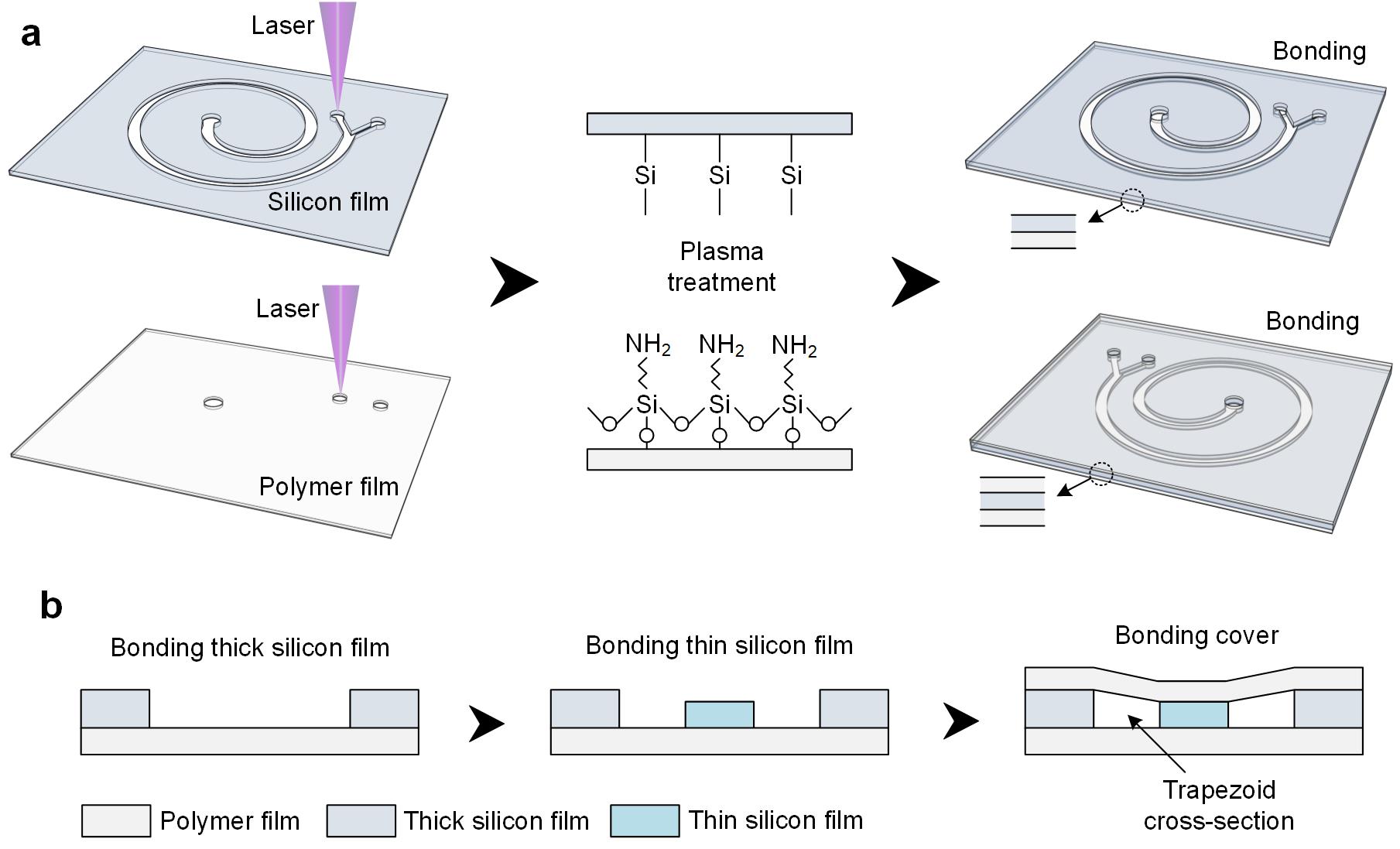

Supplement: Supplementary 1 — Figs. S1 to S35 Table S1 and S2 [file research.0431.f1.zip › Fig.S32.jpg]

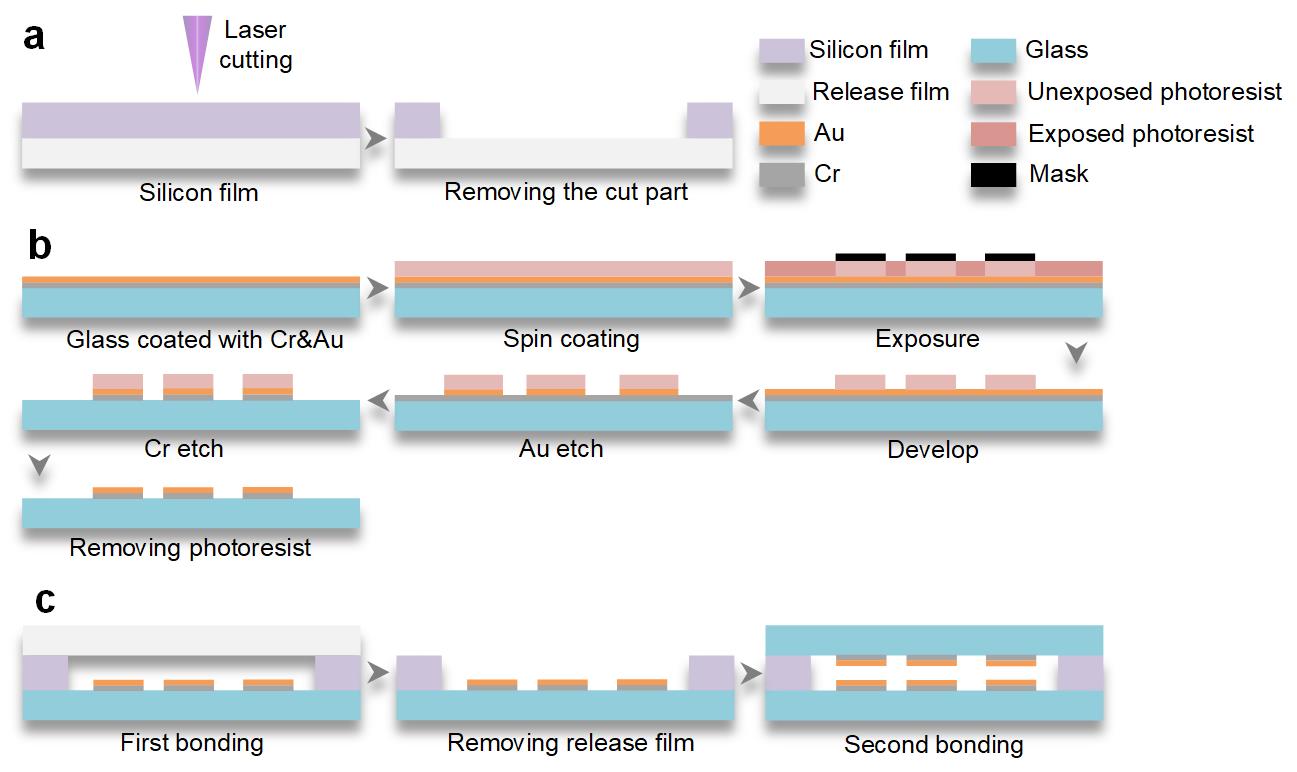

Supplement: Supplementary 1 — Figs. S1 to S35 Table S1 and S2 [file research.0431.f1.zip › Fig.S33.jpg]

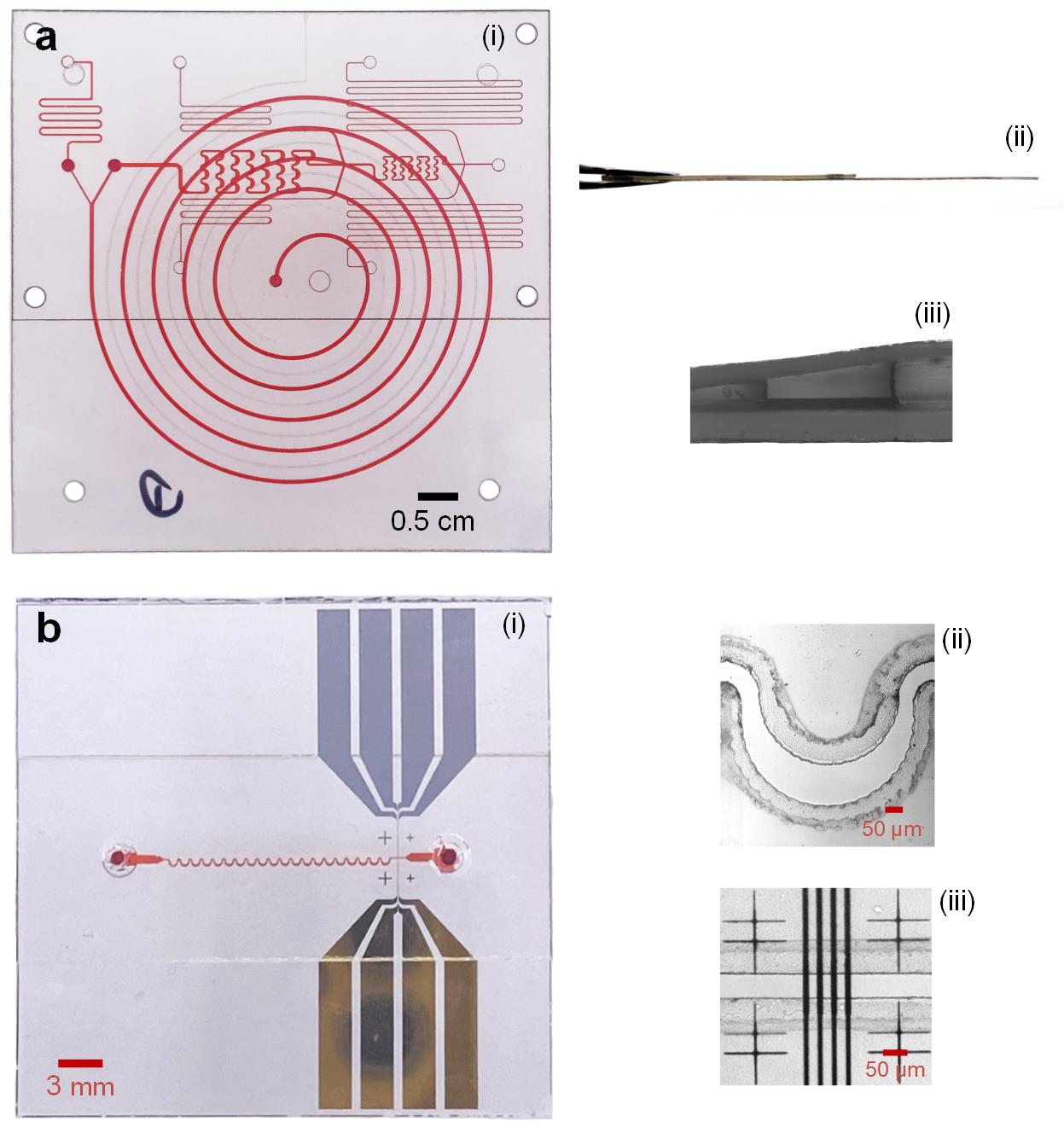

Supplement: Supplementary 1 — Figs. S1 to S35 Table S1 and S2 [file research.0431.f1.zip › Fig.S34.jpg]

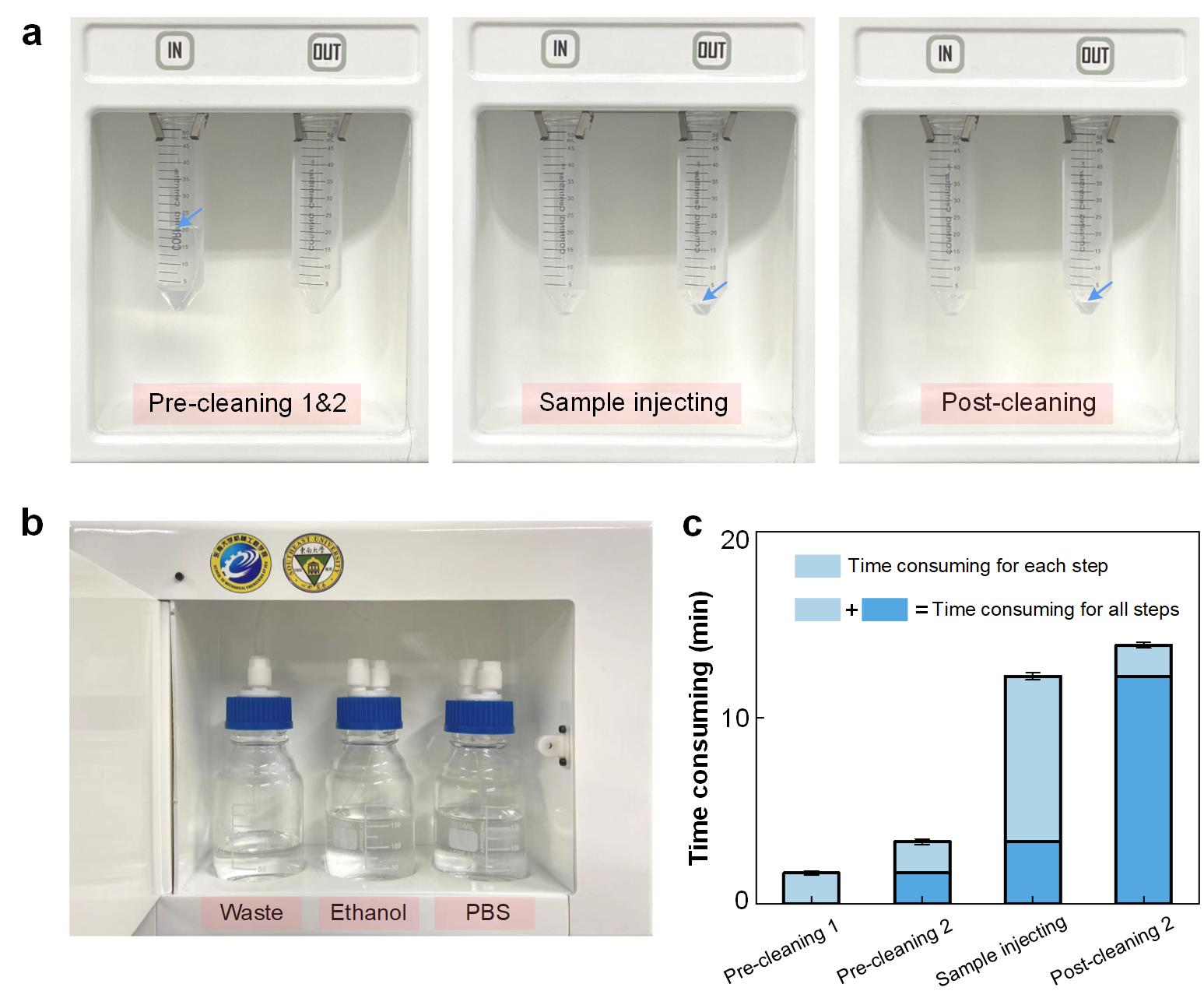

Supplement: Supplementary 1 — Figs. S1 to S35 Table S1 and S2 [file research.0431.f1.zip › Fig.S35.jpg]

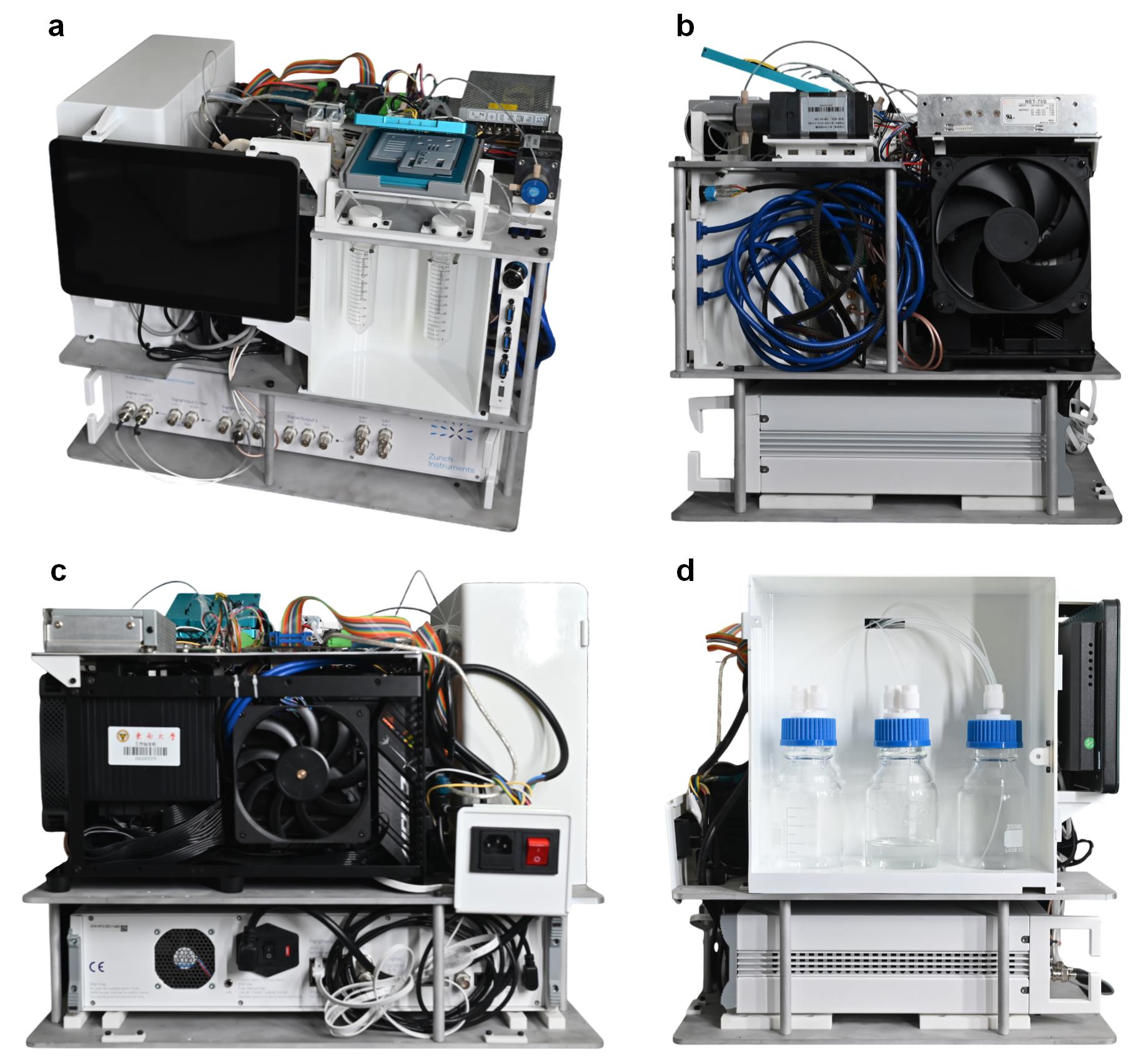

Supplement: Supplementary 1 — Figs. S1 to S35 Table S1 and S2 [file research.0431.f1.zip › Fig.S4.jpg]

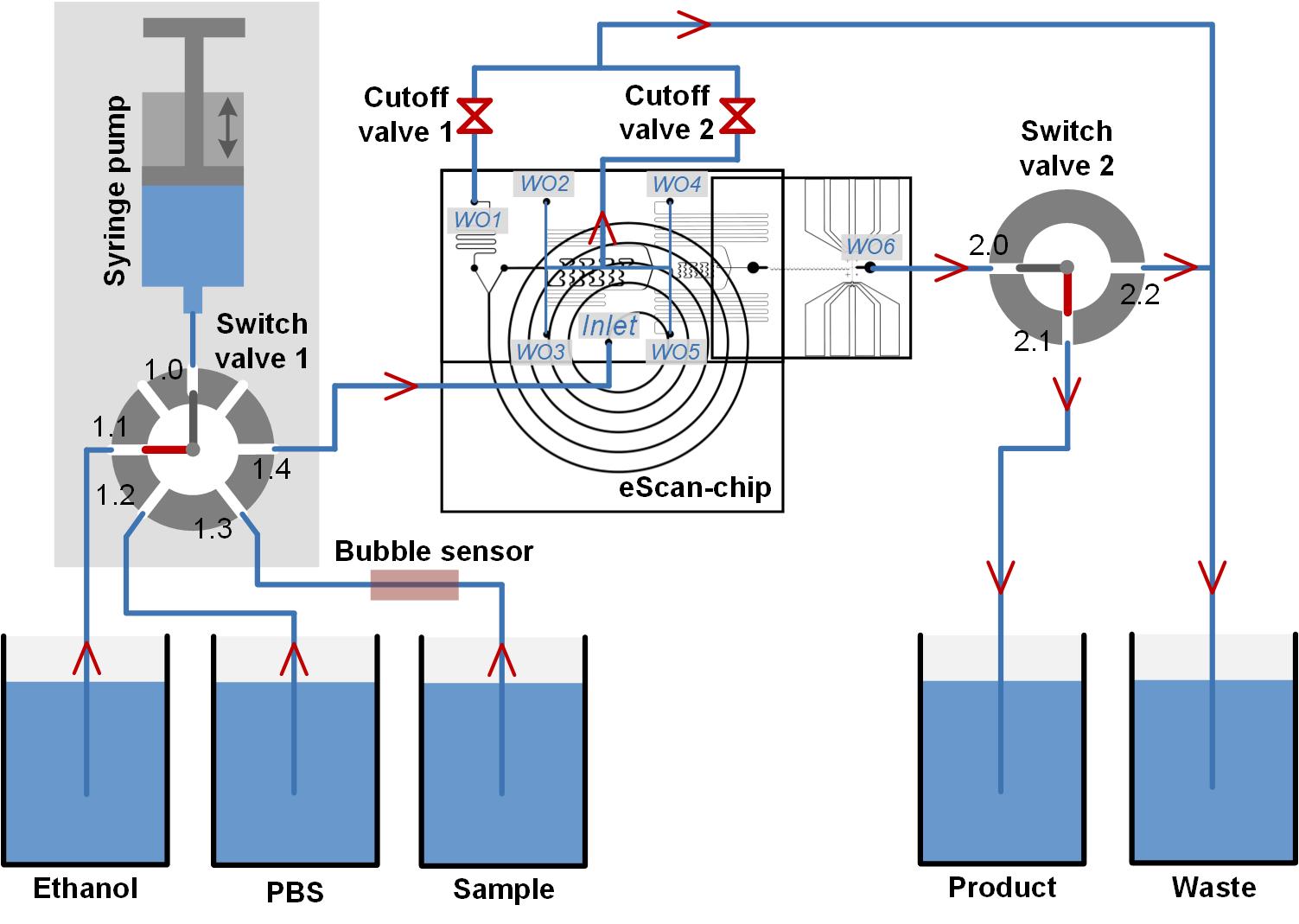

Supplement: Supplementary 1 — Figs. S1 to S35 Table S1 and S2 [file research.0431.f1.zip › Fig.S5.jpg]

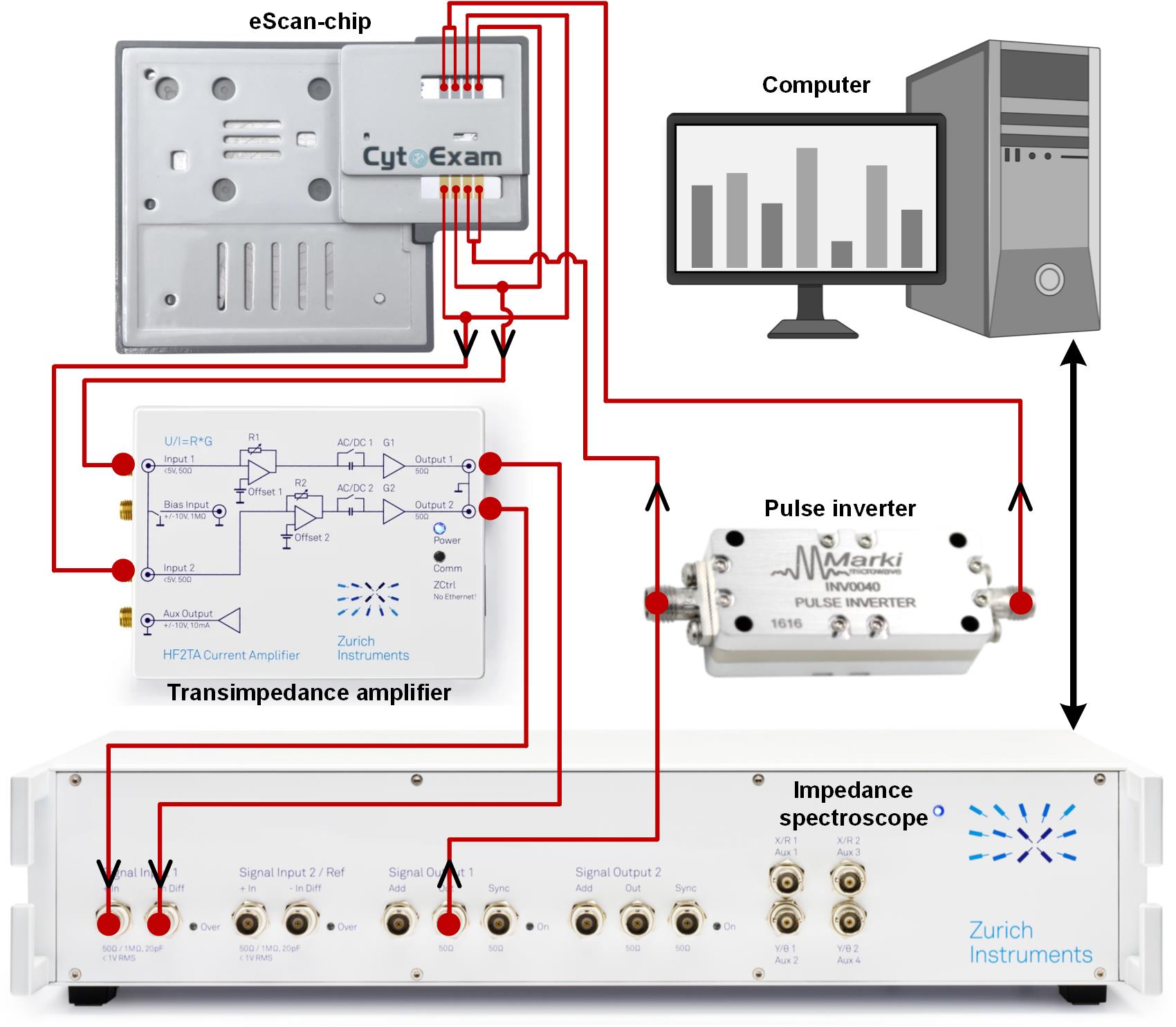

Supplement: Supplementary 1 — Figs. S1 to S35 Table S1 and S2 [file research.0431.f1.zip › Fig.S6.jpg]

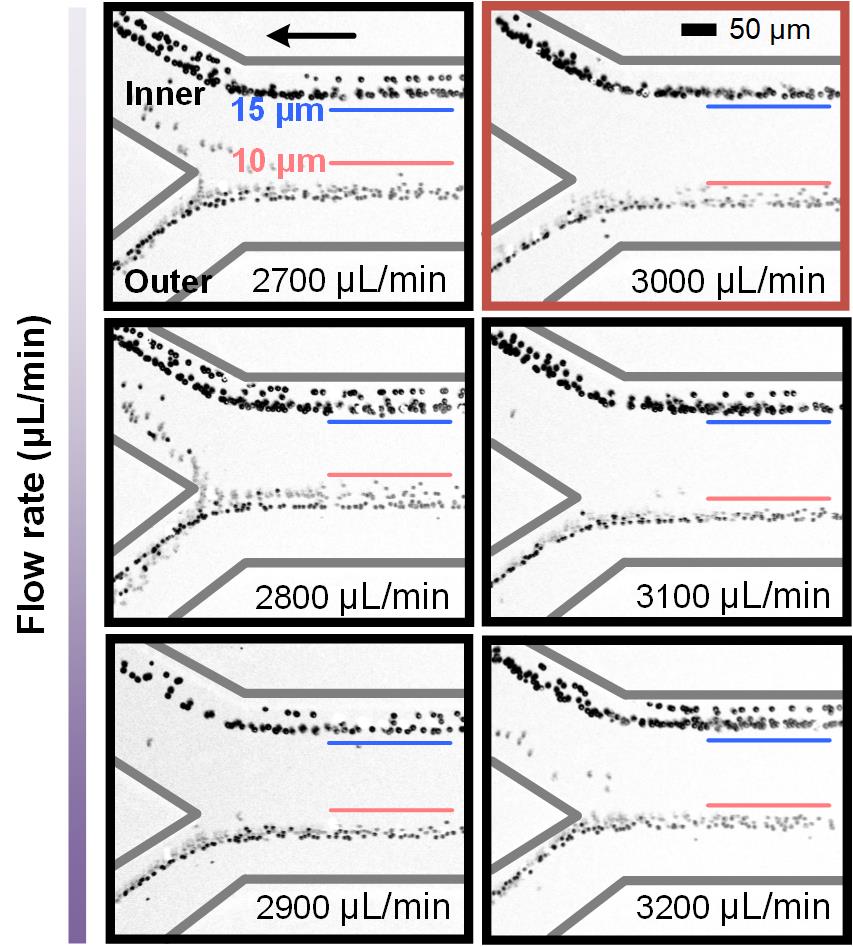

Supplement: Supplementary 1 — Figs. S1 to S35 Table S1 and S2 [file research.0431.f1.zip › Fig.S7.jpg]

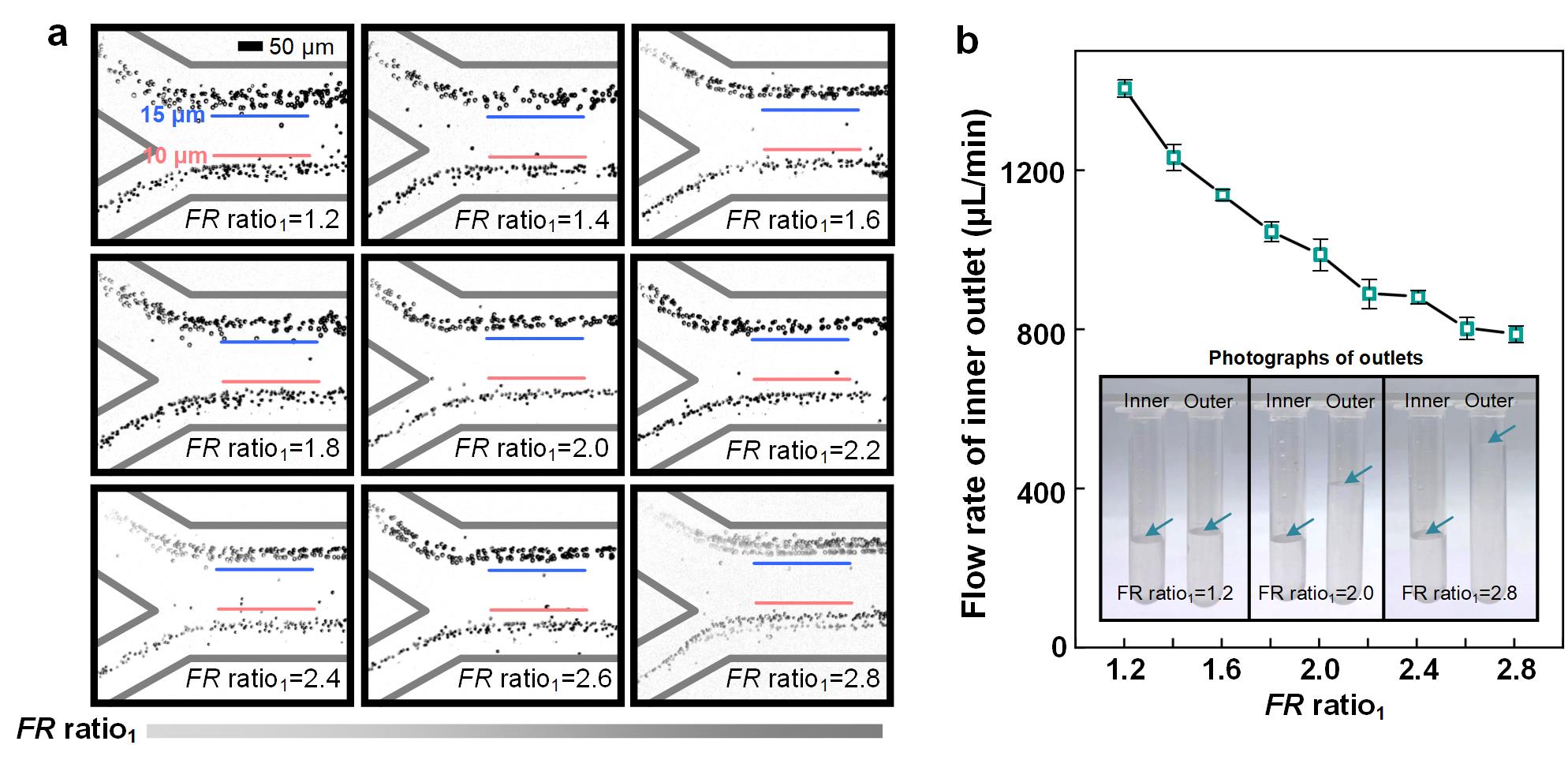

Supplement: Supplementary 1 — Figs. S1 to S35 Table S1 and S2 [file research.0431.f1.zip › Fig.S8.jpg]

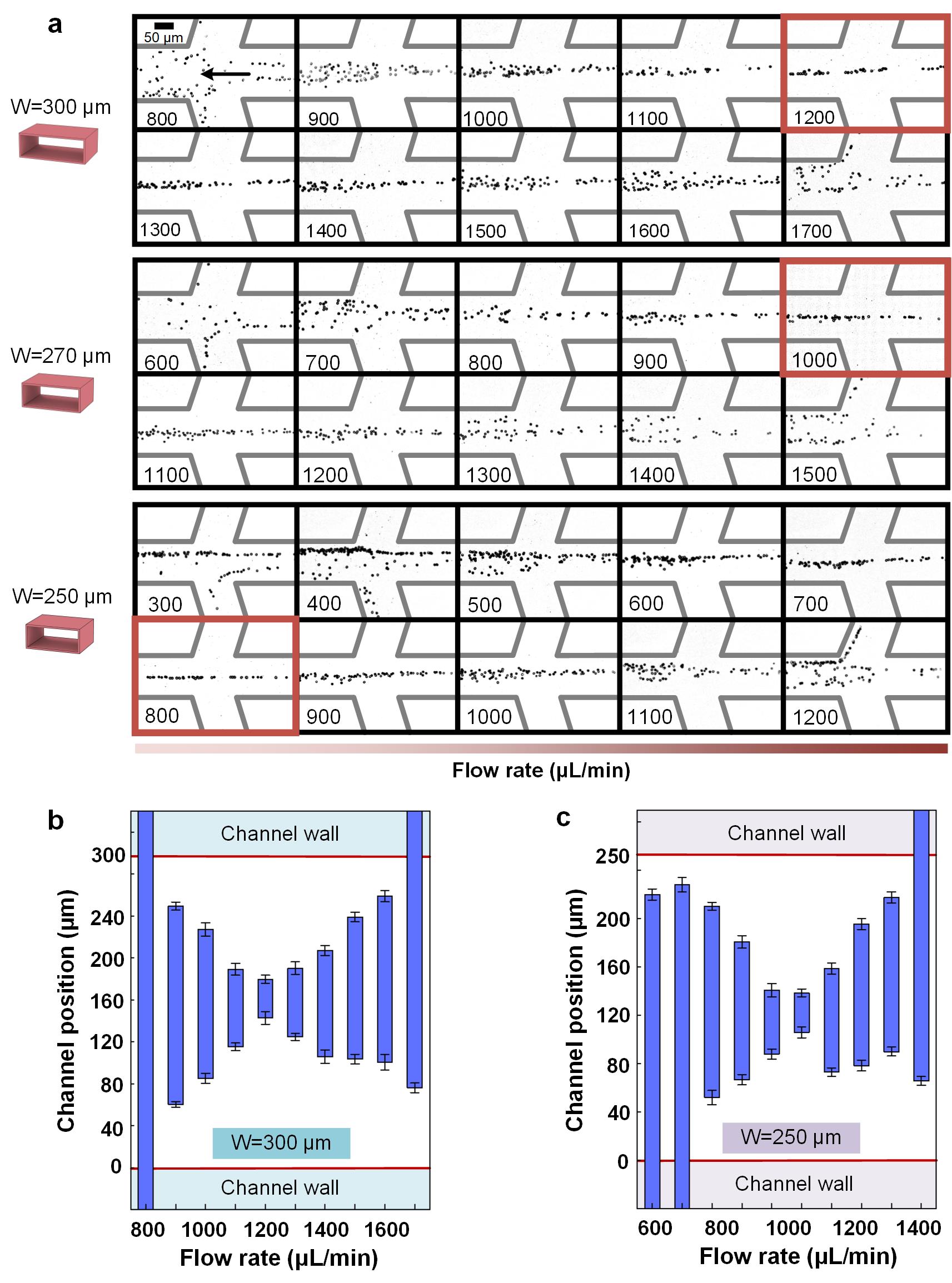

Supplement: Supplementary 1 — Figs. S1 to S35 Table S1 and S2 [file research.0431.f1.zip › Fig.S9.jpg]
